# Trajectory of health-related quality of life during and after hospitalisation due to worsening of heart failure

**DOI:** 10.1007/s11136-024-03818-6

**Published:** 2024-10-30

**Authors:** Wai Chee Kuan, Ka Keat Lim, Kok Han Chee, Sazzli Kasim, Juman Abdulelah Dujaili, Kenneth Kwing-Chin Lee, Siew Li Teoh

**Affiliations:** 1 School of Pharmacy, Monash University Malaysia, Jalan Lagoon Selatan, Bandar Sunway, 47500 Selangor Malaysia; 2 Department of Population Health Sciences, School of Life Course & Population Sciences, King’s College London, London, SE1 1UL United Kingdom; 3 Centre for Evaluation & Methods, Wolfson Institute of Population Health, Faculty of Medicine & Dentistry, Queen Mary University of London, London, E1 2AB United Kingdom; 4 Department of Medicine, Faculty of Medicine, University of Malaya, 50603 Kuala Lumpur, Malaysia; 5 Department of Internal Medicine (Cardiology), Faculty of Medicine, Universiti Teknologi MARA (UITM), Jalan Hospital, Sungai Buloh, 47000 Selangor Malaysia; 6 Cardiac Vascular and Lung Research Institute (CaVaLRI), Universiti Teknologi MARA (UITM), Jalan Hospital, Sungai Buloh, 47000 Selangor Malaysia; 7 Swansea University Medical School, Singleton Park, Swansea University, Wales, SA2 8PP United Kingdom; 8 School of Medicine, Monash University Malaysia, Jalan Lagoon Selatan, Bandar Sunway, 47500 Selangor Malaysia; 9 School of Pharmacy, Taylor’s University Malaysia, No.1, Jalan Taylor’s, Subang Jaya, 47500 Selangor Malaysia

**Keywords:** Heart failure, Health-related quality of life, EQ-5D, Utility, Visual analogue score, Hospitalisation

## Abstract

**Purpose:**

This study aimed to examine the trajectory in health-related quality of life (HRQoL) during and after hospitalisation for worsening of heart failure (HF) in Malaysia.

**Methods:**

200 patients with heart failure and reduced ejection fraction (HFrEF) admitted into two hospitals in Malaysia due to worsening of HF were surveyed using the EQ-5D-5 L questionnaire. The primary outcomes were utility values at admission, discharge and 1-month post-discharge (1MPD). Secondary outcomes included the visual analogue scores (VAS) and the proportion of patients reporting each EQ-5D-5 L dimension levels. Missing data were imputed using multiple imputation, and generalised linear mixed models were fitted.

**Results:**

At admission, the unadjusted mean utility values and VAS scores for HFrEF patients in Malaysia were as low as 0.150 ± 0.393 and 38.2 ± 20.8, respectively. After a median hospital stay of 4 days, there was a significant improvement in utility values and VAS scores by 0.510 (95% CI: 0.455–0.564) and 28.8 (95% CI: 25.5–32.1), respectively. The utility value and VAS score at 1-month post-discharge were not significantly different from discharge. The proportion of HFrEF patients reporting problems and severe problems in mobility, self-care, usual activities, and anxiety/depression, pain/discomfort reduced at varying degree from admission to discharge and 1MPD.

**Conclusion:**

HF is a progressive condition with substantial variation in HRQoL during the disease trajectory. During hospitalisation due to worsening of HF, HFrEF population has unfavourable HRQoL. Rapid and significant HRQoL improvement was observed at discharge, which sustained over one month. The study findings can inform future cost-effectiveness analyses and policies.

**Supplementary Information:**

The online version contains supplementary material available at 10.1007/s11136-024-03818-6.

## Abstract (plain language summary)

Heart failure is a progressive disease. Health-related quality of life of heart failure patients dynamically changes over time. Upon worsening of symptoms requiring hospital admission, the average level of health-related quality of life among people living with heart failure was low, approximately one-third of their perfect health. From the perspective of general public, this is equivalent to a health state that is near to being dead. Majority of patients had problems in mobility, self-care, usual activities, anxiety/depression and pain/discomfort. After receiving treatments in hospitals, most patients reported less problem in mobility, are able to take care of themselves and perform usual activities, feeling less pain/discomfort, and feeling less anxious/depressed. At discharge, their health state improved significantly by a large amount (equivalent to gaining half a year in perfect health), and this gain was sustained at 1-month post-discharge. This study provides policy makers insights on heart failure patients’ disease experiences and quantifies their health-related quality of life during and after hospital admissions based on patients’ disease experiences and general public’s preference. In this way, both patients and public were involved in the policy-making process.

**Supplementary Information:**

The online version contains supplementary material available at 10.1007/s11136-024-03818-6.

## Highlights

HF is a progressive condition with substantial variation in HRQoL during the disease trajectory.

During hospitalisation due to worsening of heart failure, people living with heart failure and reduced ejection fraction have unfavourable health-related quality of life.

After receiving treatments in hospitals, there was a rapid and significant improvement in health-related quality of life and the improvement sustained at 1-month after discharge.

**Supplementary Information:**

The online version contains supplementary material available at 10.1007/s11136-024-03818-6.

## Introduction

Heart failure (HF) is a progressive condition with substantial variation in the disease trajectory [1]. People living with HF are categorised into distinct subtypes according to left ventricular ejection fraction (LVEF). HF with reduced ejection fraction (HFrEF) is when LVEF ≤ 40%, HF with mildly reduced ejection fraction (HFmrEF) is when LVEF between 41% and 49%, and HF with preserved ejection fraction (HFpEF) is when LVEF ≥ 50%. Regardless the subtypes, the clinical course of HF is non-linear. While some patients remain symptomatically stable, 27% will develop a worsening HF event (also referred to as acute decompensation of HF) and require HF hospitalisation to reduce congestions and relieve symptoms [1]. Hospitalisation due to worsening of HF is an important event in the disease trajectory of HF. Apart from imposing huge economic burden [1–4], it is a strong and independent predictor for repeat hospitalisation and mortality [5–7] and it negatively affects patient’s health-related quality of life (HRQoL) [8–11].

HRQoL is defined as the aspects of quality of life that are directly or indirectly related to health, encompassing three main domains (i.e., physical, psychological, and social well-being) [12]. Previous studies demonstrated that the HRQoL among patients with stable HF was worse than patients with other chronic conditions, such as diabetes mellitus, cancer, or Alzheimer’s disease [13, 14]. Another study revealed that HF patients valued HRQoL over longevity and were willing to trade longer life expectancy for better HRQoL [15]. Hence, improving HRQoL has been an important treatment goal in the management of HF and quality-adjusted life years (QALY) has become an instrumental metric for direct comparison of the trade-off risk-benefits of different health technologies before country-wide adoption and implementation [16].

QALY is derived from years of life lived multiplied by a quality adjustment weight (i.e. utility value), that is used to reflect the HRQoL of a person. It ranges from “<0” (represents a health state worse than being dead) to “0” (represents death) and “1” (represents perfect health) [16–19]. It is commonly elicited through a two-step valuation method, where patients describe their health states using the generic HRQoL instruments (i.e. EQ-5D) and the general populations provide valuations for each health state. Utility value is an important model driver in cost-effectiveness analysis (CEA) [20, 21]. While HRQoL rapidly fluctuates over time [16, 20–22], understanding the HRQoL at specific points in the life of the patients, particularly important clinical events, is essential to capture the utility values relevant to the decision problems and inform CEAs.

There have been studies quantifying the changes in HRQoL of HF population during and after hospitalisation [8–11]. However, these utility data might not be generalised to the local population in Malaysia due to regional, cultural and socioeconomic heterogeneity in HRQoL. This study aimed to examine the changes in HRQoL (measured in utility values, VAS scores, and proportions reporting the levels of EQ-5D-5 L dimensions) during and after hospitalisations due to worsening of HF events in Malaysia, a multi-ethnics and multi-cultural upper-middle income country.

## Methods

All consecutive patients who were admitted from June 2022 to April 2023 to the cardiac ward in two teaching hospitals in Malaysia and with a provisional diagnosis of HFrEF were screened for eligibility to be included in this prospective cohort study. Key inclusion criteria were (1) patients aged ≥ 18 years old, (2) confirmed diagnosis of HFrEF with evidence of LVEF ≤ 40%, and (3) hospitalised due to worsening of HF. Exclusion criteria were described in Online Resource 1. This study was conducted following STROBE guidance (Online Resource 2) [23].

### Sample size calculation

Using the minimal clinically important difference for utility values of 0.2 [24], a minimum sample of 199 is required to achieve a power of 80% and a level of significance of 5%. Considering 50 predictor variables to be included in the regression model and 2 subjects per variable [25] for adequate estimation of regression coefficients, standard errors, and confidence intervals, the calculated sample size were 100. To guide the final recruitment, this study used the larger estimate, which was 199 sample size.

### Data collection

Convenience sampling was used to recruit participants. While convenience sampling may not be representative of the population at large, it allows exploration of sociocultural and clinical factors that could potentially affect the HRQoL efficiently [26]. Eligible patients were surveyed using the EQ-5D-5 L questionnaire, which has been validated for use to measure HRQoL in HFrEF population [24]. EQ-5D-5L questionnaire comprises 5 dimensions (i.e., mobility, self-care, usual activities, pain/discomfort, and anxiety/depression) and each dimension was assessed by a question with five levels of response (Level 1: no problem; Level 2: mild problem; Level 3: moderate problem; Level 4: severe problem; Level 5: extreme problem). In the second part of EQ-5D-5 L, the health status of patients was evaluated using a visual analogue scale (VAS), ranging from 0 (worst imaginable health status) to 100 (best imaginable health status). Admission and discharge EQ-5D-5L were collected via face-to-face interview while EQ-5D-5 L at 1-month post-discharge (1MPD) were obtained via phone interview. Other details of data collection were described previously [24].

Baseline demographics, disease information, comorbidities, past and current medications, and laboratory findings were obtained from the electronic medical records. NYHA was assessed at 1MPD. Informed consent was obtained from participants prior to data collection. All data were de-identified to protect patient confidentiality. Ethics approval has been granted by ethics committees of participating centres (202234 − 11050; REC/07/2022 (OT/MR/3); 32518).

### Study outcomes

The primary outcome was the utility values at admission, discharge and 1MPD. The utility values were derived by mapping the EQ-5D-5 L dimensional scores collected from HFrEF patients onto the Malaysia value sets obtained from general population through a hybrid model with time trade-off method and discrete choice experiment method) [27]. Utility values ranged from − 0.442 (represent a health state worse than death) to 0 (represent health state equivalent to death) and 1 (represent perfect health state). Secondary outcomes included VAS scores at each timepoint, mean change in utility values over time, mean change in VAS scores over time, and the proportion of patients reporting problems (Levels 2 to 5) and severe problems (Level 5) for each EQ-5D-5 L dimension at each timepoint.

### Missing data

All patients were included in the analysis, including those lost to follow-up. Missing covariates and EQ-5D-5 L data at 1MPD were imputed for 50 times using multiple imputation (MI) [6, 28–30]. Details were described in Online Resource 4.

### Statistical analysis

All variables were presented in descriptive statistics. Categorisation of variables was summarised in Online Resource 3. EQ-5D-5 L dimensions were presented in frequency and percentages. The utility values and VAS scores were estimated in mean ± standard deviation. Independent t-tests and ANOVA tests were used to examine the group differences on the mean utility values. Paired t-tests were used to compare the mean difference between the utility values and VAS scores differences between admission and discharge, and between discharge and 1MPD. To measure the change in utility values and VAS scores before and after hospitalisation for the HFrEF population, multivariable generalised linear mixed models (GLMM) [6, 31, 32] were fitted with repeated measures of utility values and VAS scores as the outcome, accounting for within-patient correlation and adjusting for variables and time of measurement. For base-case analysis (Model 1), LASSO regression (a supervised machine learning algorithm) was used to regularise all covariates and identify the important variables that could confound the outcomes to be adjusted in the model and prevent overfitting and multicollinearity [33, 34]. Details of LASSO regression and the important variables were described in Online Resource 5. To estimate the marginal mean utility values and VAS scores for HFrEF patients for each timepoint, post-hoc analysis was conducted using Kenward-Roger method due to its acceptable Type 1 error rates even for small samples [35]. All analyses were performed using R, with statistical significance set at a *p* < 0.05.

### Sensitivity analyses

Two sensitivity analyses were conducted. First, we explored model adjusted with variables selected based on LASSO regression using all available (AV) datasets (Model 2). Secondly, we explored model adjusted with variables selected based on literature and clinical relevance using MI datasets (Model 3). Variables that were statistically significant at the *p* < 0.05 level in univariate analyses and clinically relevant [6, 24, 36, 37] were included in multivariable regression models, while age, gender, ethnics, language of EQ-5D-5 L [24] were always retained. List of variables were in Online Resource 6.

## Results

**Fig. 1:**
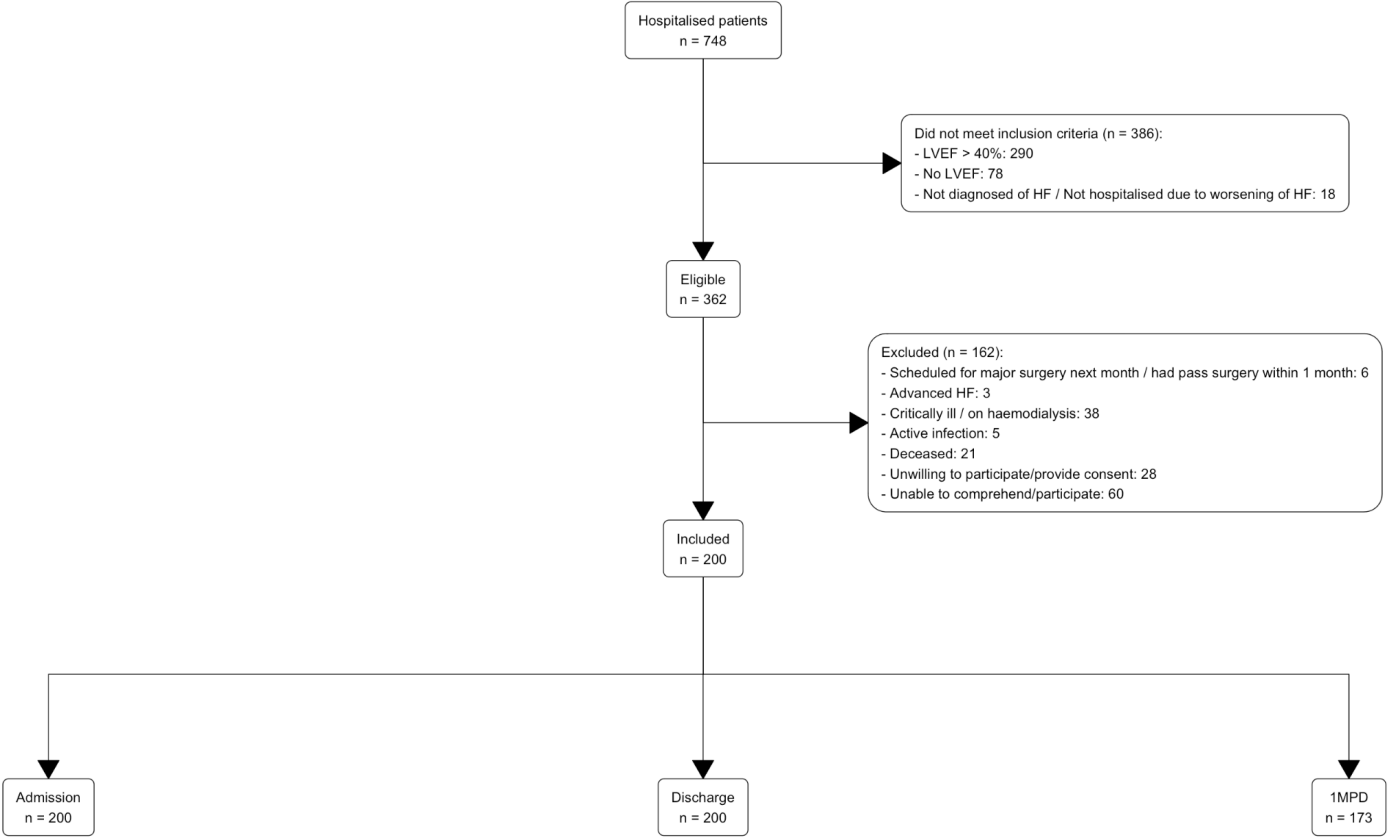
Study participants flowcharts

Of the 748 patients who were hospitalised in cardiac wards during the study timeframe, 200 patients were surveyed, and 173 patients (86.5%) were followed up at 1MPD (Fig. 1). The median age of study participants was 61.4 years old (40% were less than 60 years old), 74% were male and 55% were Malay ethnicity (Table 1), similar to the characteristics of general Malaysian HF patients (Online Resource 7A). Patients who were lost to follow up at 1MPD (*n* = 27) appeared to have poorer prognosis (Online Resource 7B) with lower utility values and VAS scores at admission and discharge, compared with those who had complete EQ-5D-5 L data at 1MPD (*n* = 173).

**Table 1:** Baseline demographics of study participants

Demographics	Descriptive Statistics (*n* = 200)
Mean age (SD)	61.4 (13.7)
Age ≥ 60 years old, *n* (%)	121 (60.5)
Male, *n* (%)	148 (74.0)
Ethnicity, *n* (%)	
Malay	110 (55.0)
Chinese	56 (28.0)
Indian / Others	33 (16.5)
Others	1 (0.5)
EQ-5D-5 L language	
Malay	98 (49.0)
Chinese	50 (25.0)
English	52 (26.0)
Married, *n* (%)	170 (85.0)
Working (*n*, %)	53 (26.5)
Income group (*n*, %)^§^	
B40 (< USD1017)	185 (92.5)
M40 (USD1017-USD2299)	4 (2.0)
T20 (≥ USD2299)	6 (3.0)
Unknown	5 (2.5)
Smoking (*n*, %)	
Non-smoker	99 (49.5)
Current active smoker	42 (21.0)
Ex-smoker	59 (29.5)
Median BMI, kg/m^2^ (IQR)	25.1 (22.2, 29.4)
Ischaemic heart failure, *n* (%)	133 (66.5)
De novo HF, *n* (%)	54 (27.0)
Years since HF diagnosis < 1 year, *n* (%)	125 (62.5)
Prior HF hospitalisation, *n* (%)	119 (59.5)
Median length of hospital stay, days (IQR)	4.0 (3.0, 6.3)
CCI scores ≥ 3, *n* (%)	144 (72.0)
Comorbidities, *n* (%)	
Hypertension	130 (65.0)
Type 2 Diabetes	128 (64.0)
Dyslipidaemia	73 (36.5)
Prior ischaemic heart disease	118 (59.0)
Prior stroke / TIA	23 (11.5)
Atrial fibrillation	49 (24.5)
Chronic kidney disease	60 (30.0)
Lung diseases (COPD / Asthma)	17 (8.5)
Anaemia	103 (51.5)
Mean LVEF, % (SD)	26.6 (7.6)
Baseline laboratory level, at admission	
Median serum creatinine, umol/l (IQR)	108.0 (85.8, 151.8)
Median eGFR, ml/min/1.73m^2^ (IQR)	61 (39.8, 81.0)
Mean haemoglobin, g/dL (SD)	12.8 (2.2)
Mean SBP at discharge, mmHg (SD)	121 (18)
Pre-admission medication, *n* (%)	
Diuretics	115 (57.5)
ACEI/ARB	86 (43.0)
ARNI	47 (23.5)
Beta-blocker	143 (71.5)
MRA	88 (44.0)
SGLT-2i	87 (43.5)
Discharged medication, *n* (%)	
Diuretics	155 (77.5)
ACEI/ARB	63 (31.5)
ARNI	81 (40.5)
Beta-blocker	178 (89.0)
MRA	157 (78.5)
SGLT-2i	146 (73.0)
NYHA at 1MPD, *n* (%)	
Class I	72 (36.0)
Class II	55 (27.5)
Class III	20 (10.0)
Class IV	26 (13.0)
Unknown	27 (13.5)

Data presented in *n* (%) unless otherwise stated. 1MPD: 1-month post-discharge; ACEI: angiotensin converting enzyme inhibitor; ARB: angiotensin receptor blocker; ARNI: angiotensin receptor neprilysin inhibitor; BMI: body mass index; CCI: Charles Comorbidity Index; COPD: chronic obstructive pulmonary disease; eGFR: estimated glomerular filtration rate; HF: heart failure; IQR: Interquartile range; LVEF: left ventricular ejection fraction; MRA: mineralocorticoid receptor antagonist; n: number of patients; N: total number of patients; NYHA: New York Heart Association; SGLT-2i: sodium-glucose co-transporter-2 inhibitor; SD: standard deviation; SBP: systolic blood pressure; TIA: transient ischaemic attack; VAS: visual analogue scale. eGFR was calculated based on MDRD equation

^§^In Malaysia, the monthly income level was categorised into B40 (< RM4850), M40 (RM4850-RM10959) and T20 (≥ RM10960). USD was presented in the table, with the exchange rate of MYR1 = USD 0.21 as of 19/10/2023

### Utility values

**Fig. 2:**
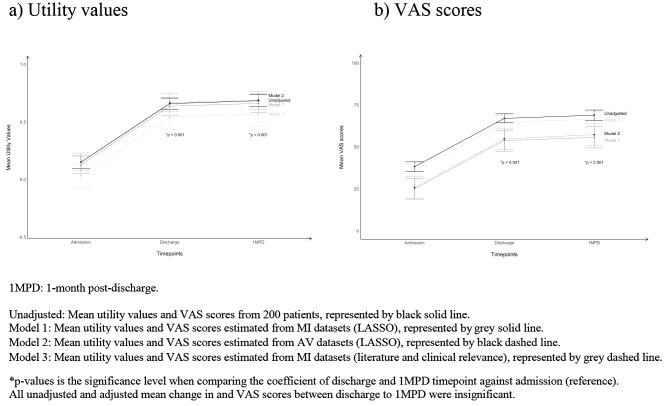
The change in utility values and VAS scores over time

The unadjusted mean utility value of HFrEF patients at admission was 0.150 ± 0.392 (Table 2), corresponding to a health state near to death. After a median hospital stay of 4 days, the unadjusted mean utility value of HFrEF patients significantly improved by 0.510 (95% CI: 0.455–0.564), which was equivalent to half a year of perfect health. At 1MPD, the unadjusted mean utility values of both MI and AV datasets were not significantly different from discharge. Subgroup-level utility values were summarised in Online Resource 8. Compared to those who were not working, HFrEF patients who were working before hospitalisation has consistently higher utility values across three time-points, suggesting that work is an independent predictor of HRQoL irrespective of time. Univariate analysis found that patients who have higher number of comorbidities, particularly chronic kidney disease (CKD) and anaemia, have lower utility values. Patients discharged with ARNI and beta-blocker had higher utility values (Online Resource 9). After adjusting for covariates (Online Resource 10), the mean utility value at admission estimated by Model 2 was the highest at 0.152 (95% CI: 0.075-0.230), followed by Model 1 at 0.127 (95% CI: 0.049–0.206) and Model 3 at 0.033 (-0.074–0.139) (Table 2). Despite small variations in the mean utility values at admission, the mean change in utility values (estimated by Model 1, Model 2 and Model 3) was consistent with the unadjusted values, ranging from 0.510–0.514 between admission and discharge and 0.022–0.024 between discharge and 1MPD. (Fig. 2).

**Table 2:** Mean utility values at different time-points

	Mean	SD / 95% CI	Mean change	95% CI	*p*-value
Unadjusted model					
Admission	0.150	0.392	Reference		
Discharge	0.659	0.342	0.510	0.455–0.564	< 0.001
1MPD	0.684^**†**^	0.379	0.534^**‡**^	0.467–0.601	< 0.001
Model 1) Base-case: MI datasets (LASSO)^¶^					
Admission	0.127	0.049–0.206	Reference		
Discharge	0.637	0.558–0.715	0.510	0.454–0.565	< 0.001
1MPD	0.661	0.580–0.743	0.534	0.475–0.593	< 0.001
Model 2) Sensitivity analysis: AV datasets (LASSO)^¶^					
Admission	0.152	0.075–0.230	Reference		
Discharge	0.666	0.589–0.744	0.514	0.456–0.573	< 0.001
1MPD	0.689	0.611–0.766	0.536	0.478–0.595	< 0.001
Model 3) Sensitivity analysis: MI datasets (literature and clinical relevance)					
Admission	0.033	-0.074–0.139	Reference		
Discharge	0.542	0.436–0.649	0.510	0.454–0.565	< 0.001
1MPD	0.567	0.459–0.675	0.534	0.475–0.593	< 0.001

^**†**^Using complete EQ-5D-5L datasets (*n* = 173), the unadjusted mean utility value at 1MPD was 0.712 ± 0.363

^**‡**^Using complete EQ-5D-5L datasets (*n* = 173), the unadjusted mean change in utility value between admission and 1MPD was 0.538 (95% CI: 0.471–0.606). Using MI EQ-5D-5L datasets (*n* = 200), the unadjusted mean change in utility value between discharge to 1MPD was insignificant at 0.025 (95% CI: -0.028–0.077). Using complete EQ-5D-5L datasets (*n* = 173), the unadjusted mean change in utility value between discharge to 1MPD was insignificant at 0.022 (95% CI: -0.0285– 0.070)

^¶^Model 1: AIC = 319, BIC = 447; Model 2: AIC = 226, BIC = 349; Model 3: AIC = 342, BIC = 470

1MPD: 1-month post-discharge; AIC: Akaike information criterion; AV: all available; BIC: Bayesian information criterion (BIC);  CI: confidence interval; LASSO: Least Absolute Shrinkage and Selection Operator. MI: multiple imputation; SD: standard deviation

### VAS scores

On a scale of 0 to 100, the unadjusted mean VAS score of HFrEF patients at admission was 38.2 ± 20.8 (Table 3). This corresponds to one-third of perfect health. Upon discharge, the unadjusted mean VAS score of HFrEF patients nearly doubled to 67.0 ± 19.2 at discharge. Similar to utility values, there was no significant difference in the unadjusted mean VAS score between discharge and 1MPD for both MI and AV datasets. Subgroup-level VAS scores were summarised in Online Resource 11. Interestingly, patients with HFrEF and anaemia had lower VAS scores, compared with those without anaemia across all time-points. Univariate analysis of VAS scores showed similar trends as utility values (Online Resource 12). After adjusting for covariates (Online Resource 13), Model 1 estimated the lowest mean VAS scores at admission which was 25.0 (95% CI: 18.9–13.1), followed by Model 2 at 25.9 (95% CI: 19.5–32.4) and Model 3 at 35.9 (95% CI: 31.5–40.2). The change in VAS scores over time was similar to the utility values (Fig. 2).

**Table 3:** Mean VAS scores at different time-points

	Mean	SD / 95% CI	Mean change	95% CI	*p*-value
Unadjusted model					
Admission	38.2	20.8	Reference		
Discharge	67.0	19.2	28.8	25.5–32.1	< 0.001
1MPD	68.9^†^	22.0	30.7^**‡**^	26.5–34.9	< 0.001
Model 1) Base-case: MI datasets (LASSO)					
Admission	25.0	18.9–31.1	Reference		
Discharge	53.8	47.4–59.9	28.8	25.3–32.3	< 0.001
1MPD	55.7	49.4–62.0	30.7	27.1–34.3	< 0.001
Model 2) Sensitivity analysis: AV datasets (LASSO)					
Admission	25.9	19.5–32.4	Reference		
Discharge	54.8	48.4–61.2	28.9	25.3–32.5	< 0.001
1MPD	57.3	50.9–63.7	31.3	27.8–34.9	< 0.001
Model 3) Sensitivity analysis: MI datasets (literature and clinical relevance)					
Admission	35.9	31.5–40.2	Reference		
Discharge	64.6	60.3–69.0	28.8	25.3–32.3	< 0.001
1MPD	66.6	62.1–71.0	30.7	27.1–34.3	< 0.001

^**†**^Using complete EQ-5D-5 L datasets (*n* = 173), the unadjusted mean VAS score at 1MPD was 70.1 ± 20.6

^**‡**^Using complete EQ-5D-5 L datasets (*n* = 173), the mean change in unadjusted mean VAS score between admission and 1MPD was 31.3 (95% CI: 27.2–35.5). Using MI EQ-5D-5 L datasets (*n* = 200), the unadjusted mean change in VAS score between discharge to 1MPD was insignificant at 1.9 (95% CI: -1.4– 5.2). Using complete EQ-5D-5 L datasets (*n* = 173), the unadjusted mean change in VAS score between discharge to 1MPD was insignificant at 2.5 (95% CI: -0.59– 5.5)

Model 1: AIC = 5250, BIC = 5378; Model 2: AIC = 4501, BIC = 4625; Model 3: AIC = 5271, BIC = 5377

1MPD: 1-month post-discharge; AIC: Akaike information criterion; AV: all available; BIC: Bayesian information criterion (BIC); CI: confidence interval; LASSO: Least Absolute Shrinkage and Selection Operator. MI: multiple imputation; SD: standard deviation

### EQ-5D-5 L dimensional levels

**Fig. 3:**
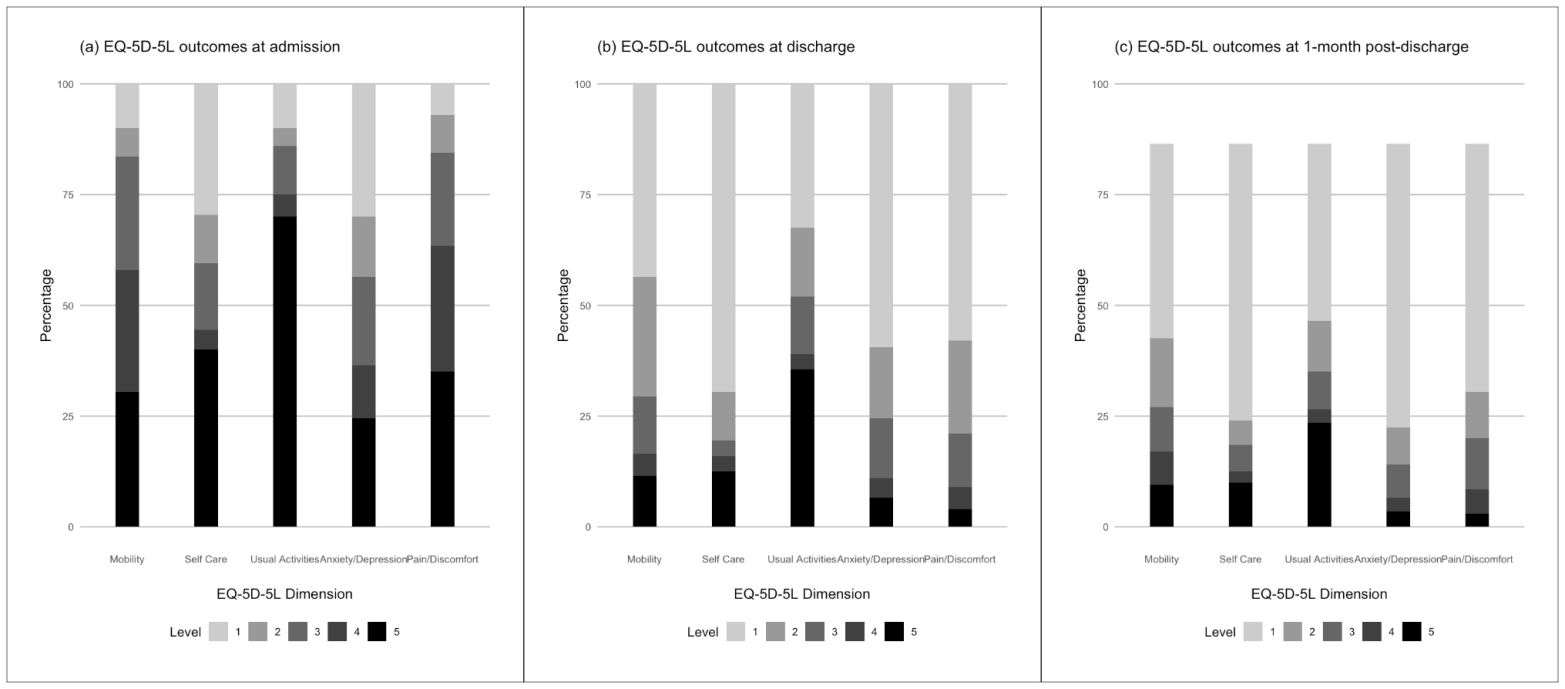
The change in EQ-5D-5 L dimensional levels over time

Figure 3 showed the changes in EQ-5D-5 L dimension levels of patients admitted to hospital due to worsening of HF. The proportion of patients reporting problem and severe problem decreased at varying degree from admission to discharge and 1MPD across all dimensions (Table 4). At admission, patients were severely affected by limitation in usual activities (70%), followed by self-care (40%) and pain/discomfort (35%) at admission. Irrespective of severity, the commonly affected dimensions were pain/discomfort (93%), followed by problem in performing usual activities (90%), and mobility (90%). Upon discharge, problems in usual activities (67.5%), mobility (56.5%), pain/discomfort (42%), anxiety/depression (40.5%) and self-care (30%) remained. Despite improvement over time, a considerable proportion of patients continued to have persistent problems in performing usual activities at discharge (any problem: 67.5%; severe problem: 35.5%) and 1MPD (any problem: 46.5%; severe problem: 27.2%). Notably, the dimensions with the most apparent change for severe problems from admission to discharge were pain/discomfort (reduced by 89%), followed by anxiety/depression (reduced by 73%) and self-care (reduced by 69%). The dimensions with the least apparent change for severe problems from discharge to 1MPD were mobility (reduced by 4%) and self-care (reduced by 7%).

**Table 4:** The percentage of patients encountering any problem and severe problem over time

Category	Admission (*n* = 200)	Discharge (*n* = 200)		1MPD (*n* = 173)	
	Any Problem	Any Problem	% change^†^	Any Problem	% change^††^
Mobility	180 (90%)	113 (56.5%)	-37%	85 (42.5%)	-25%
Self-care	141 (70.5%)	61 (30.5%)	-57%	48 (24%)	-21%
Usual Activities	180 (90%)	135 (67.5%)	-25%	93 (46.5%)	-31%
Anxiety/Depression	140 (70%)	81 (40.5%)	-42%	45 (22.5%)	-44%
Pain/Discomfort	186 (93%)	84 (42%)	-55%	61 (30.5%)	-27%
Category	Severe problem	Severe problem	% change^†^	Severe problem	% change^††^
Mobility	61 (30.5%)	23 (11.5%)	-62%	19 (11.0%)	-4%
Self-care	80 (40.0%)	25 (12.5%)	-69%	20 (11.6%)	-7%
Usual Activities	140 (70.0%)	71 (35.5%)	-49%	47 (27.2%)	-23%
Anxiety/Depression	49 (24.5%)	13 (6.5%)	-73%	7 (4.0%)	-38%
Pain/Discomfort	70 (35.0%)	8 (4.0%)	-89%	6 (3.5%)	-13%

^**†**^The difference in percentage from admission

^**††**^The difference in percentage from discharge

## Discussion

This longitudinal study provides insights into the trajectory of HRQoL of patients living with HFrEF in Malaysia, a multi-ethnics and multi-cultural upper-middle income country. Worsening HF significantly impacted the HRQoL of patients, approaching a state near to death from the perspective of general public (utility scores) and one-third of perfect health from patients’ viewpoint (VAS scores). After receiving care in the hospital, rapid and significant HRQoL improvement was observed among survivors, and the improvement sustained over 1 month. This study offers several recommendations from discharge planning to continuity of care to improve HFrEF patients’ overall outcomes. The time-specific subgroup-level utility values estimated in this study can inform future CEAs.

Upon admission, over two-thirds of patients experienced severe limitations in daily activities. Two in five were unable to self-care and necessitated complete reliance on caregivers and over one-third had severe pain and discomfort. These combinations led to an average low level of HRQoL during HF worsening. From the perspective of the general population, this type of HRQoL approaches a state akin to being dead and from the viewpoint of patients, it was one-third of their perfect health. This study finding is largely consistent with many previous studies that showed hospitalisation due to worsening HF led to a steep declination of HRQoL [8–11]. Specifically, HF hospitalisation led to broad and marked impairments in physical function, frailty and decreased cognitive functions, which limited patients’ ability to perform daily activities and self-care [9, 11]. Intense pain/discomfort is likely due to the systemic effects of acute decompensation HF mediated through activation of haemodynamic, inflammatory, neurohormonal, nutritional pathways, decreasing cardiac output and increasing fluid accumulation, proinflammatory cytokines and catecholamines, acute skeletal muscle wasting and hepatic injury [9, 38, 39]. A meta-analysis suggested that Hospital at Home (HaH), a model in which patients receive intravenous diuretics and treatments in their homes with daily healthcare professional visits and monitoring, improved HRQoL and reduced hospitalisation costs without adverse impact on mortality in patients with acute decompensated HF, compared with routine hospitalisation [40, 41]. A recent study also demonstrated that HaH may be more concordant with the values and preferences of patients [42]. While the feasibility, clinical and economic implications of HaH model in Malaysia remain unknown, further research needs to strengthen the evidence base of HaH model, including the provider and patients’ preference.

After receiving in-hospital treatments, HFrEF patients experienced rapid and significant HRQoL improvement. Utility values indicated an increase equivalent to half a year of perfect health while VAS scores doubled from baseline, consistent with previous studies [9, 11, 38]. Notably, severe pain/discomfort and anxiety/depression were decreased by 73% and 89% at discharge. Despite worsening of HF negatively impacting the physical and psychological well-being, patients were able to cope quickly after initiation of medical treatments such as intravenous diuretics, vasoactive inotropes, inodilators, and vasodilators. Notwithstanding improved HRQoL at discharge, some levels of impairment persisted at discharge, particularly on usual activities, mobility, and pain/discomfort. Implementing cost-effective cardiac rehabilitation, a multidimensional care model that included exercise training, self-care education, nutritional counselling and psychosocial support, at discharge is crucial to facilitate the recovery of patients and reduce dependence on caregivers. While our study showed that improvement in severe problem in mobility and self-care mostly occurred during the course of hospitalisation and less so after discharge and REHAB-HF trial [8, 43] demonstrated that an early, tailored and transitional physical rehabilitation program that focused on leg strength, balance, mobility, and endurance significantly improved patients’ HRQoL over the first 3 months from discharge, early cardiac rehabilitation is recommended before discharge. Persistent and continuous efforts are crucial in the implementation of rehabilitation to improve the overall HRQoL, given their dose-dependence relationship [44].

In this study, the HRQoL of HFrEF patients at 1-month after hospitalisation was notably poorer (utility value: 0.68; VAS score: 68.9), compared with the general elderly population (utility value: 0.89; VAS score: 83.7) [45] and the ambulatory HF patients attending outpatient clinics in Malaysia (utility value: 0.82; VAS score: 77.1) [46]. Despite absence of baseline EQ-5D-5 L data prior to hospitalisation in this study, a post-hoc analysis on two clinical trials [10] that analysed HRQoL data before and after hospitalisation found that upon recovery, patients had slightly lower level of HRQoL than the baseline value before the hospitalisation. The lower level of HRQoL in patients recently discharged from hospital due to a worsening HF than ambulatory patients are likely due to the combined effects of pre-existing impairments from chronic HF, aging and comorbidities, and the residual systemic effects of acute decompensation HF, exacerbated by hospitalisation, prolonged bed-bound and persisted muscle wasting [9]. Another study also revealed that patients living with HFrEF often relate their overall HRQoL to moments of crisis including hospitalisation and anchor all subsequent experiences to previous events [47]. The significant impact of hospitalisation on HRQoL [8–11] and prognosis (i.e. increased risk of mortality and rehospitalisation) [48] supports shifting the priority of HF management towards preventing repeat HF hospitalisation.

Previous studies have demonstrated that initiation of guideline-directed medical therapies (GDMT) including ACEI/ARB/ARNI, beta-blockers, MRA, SGLT-2i lowered hospitalisations rates in HF patients [49–52]. The STRONG-HF trial showed that early initiation and titration of GDMT (at least half the target dose within 2 days before anticipated hospital discharge) not only reduced readmissions for HF or all-cause mortality but significantly improved patients’ HRQoL within 6 months [53], supporting adopting intensive GDMT with rapid up-titration and close follow-up in patients hospitalised due to worsening of HF. In our study, patients who were discharged with ARNI and beta-blockers had significantly better HRQoL after 1 month. The improvement in health status following treatment with ARNI and beta-blockers are also evident with large clinical trials [54–58]. In Malaysia, the utilisation of beta-blocker seems satisfactory, but the adoption of ARNI remains low due to cost and access barriers [59]. Enhancing haemodynamic stable HFrEF patients who had a hospitalisation due to worsening of HF access to ARNI in Malaysia could potentially improve patients’ HRQoL and overall outcomes. Generating cost-effectiveness evidence of ARNI helps inform the adoption decision. Given the substantial impact of HF hospitalisation on HRQoL, omitting the effect of HF hospitalisation on the utility values in CEAs, particularly interventions that were clinically proven to reduce HF hospitalisation, could potentially underestimate the cost-effectiveness of HF technologies and services.

In our study, over 70% of HFrEF patients have CCI scores ≥ 3 and patients with CKD and anaemia consistently reported lower levels of HRQoL, compared with those without the comorbidities [60, 61]. Apart from differences in HRQoL, previous studies have also shown that heart failure patients with CCI scores > 2.97, CKD and anaemia are predictors of poor prognosis [62, 63]. Given that anaemia is a modifiable risk factor, anaemia correction before discharge may represent a major opportunity to improve the HRQoL and long-term outcomes of patients admitted for worsening HF. MY-HF registry reported a large substantial proportion of HF patients with multimorbidity (particularly CKD) and multimorbidity complicates the management of HF [59], a personalised multimorbidity model, including cardiac, pulmonary, renal and haematological rehabilitation, presents an appealing opportunity to increase healthcare efficiency and improve patients’ overall outcomes [64–66]. Deliberate planning is needed to develop an efficient personalised multimorbidity model in the primary care settings and map out seamless patient journey transition from hospital to primary clinics, reducing hospitalisation and decentralising care from tertiary centres.

There has been an increasing incidence of HF in younger adults [67] and 25% patients at the working age old did not return to work after the first hospitalisation for heart failure [68]. Our study finding that HFrEF patients who were in the workforce prior to hospitalisation had better HRQoL throughout the disease trajectory and a Danish study finding that working patients had lower mortality, when compared with those not working [68], strongly suggest the inclusion of return to work as a treatment goal in the management plan of patients living with HFrEF. To encourage patients who are fit to resume work as early as possible, a multidisciplinary and coordinated vocational rehabilitation that individualises the return-to-work plans for patients is worth exploring. Multi-level support from various stakeholders including employers, social care and patient advocacy group is needed to reduce frictions associated with return to work [69, 70].

### Study strengths

First, this real-world cohort study enriches the findings from international clinical trials. Despite clinical trials representing an excellent opportunity to measure changes in utility over time, the transient effect of acute HF hospitalisation on utility values is often not captured in clinical trials [16, 19] due to the unparalleled timing between regular follow-up schedule and the timing of acute events. This was observed in the HRQoL analysis from TENS-HMS trial [71] where hospitalisation did not significantly affect patients’ HRQoL. Besides that, the trial-derived utility data could not be generalised to all populations given the population heterogeneity and patients who were excluded from the trial might be eligible for treatment in routine practice [16, 19]. By measuring the EQ-5D-5 L at specific timepoints during HF hospitalisation and estimating the utility values using local value sets, this study sheds light on the trajectory of HRQoL in people living with HFrEF and offers several evidence-based recommendations for the management of HFrEF in Malaysia, a multi-ethnics and multi-cultural upper-middle income country. By considering patients’ disease experiences and quantifying the HRQoL through general public’s valuation of health state, this study involves both patients and public in the local policy-decision process for the management of HF.

Secondly, our study provides adequate validity and reliability for estimating the mean change in utility values and VAS scores in the local context to inform future CEAs [6, 31, 32]. The study participants’ distribution adequately represents the general population of Malaysia by resembling the characteristics from National Heart Institute, a major cardiac tertiary centre and referral centre for advanced HF care in Malaysia and the Malaysian Heart Failure (MY-HF) registry [72], which involved 18 participating sites in Malaysia. Besides that, this study recruited adequately homogenous sample in a similar clinical setting (teaching hospitals) and there were exhaustive patients’ baseline characteristics which allow adjustment for between-patient variations and within-patient correlation using GLMM. Lastly, our study included two sensitivity analyses to explore the impact of adjusting for different variables (identified through LASSO regression and literature) and using different approaches (MI and AV datasets) in addressing missing data as recommended by Mukherjee et al. [30]. Despite small differences in the mean utility values and VAS scores between base-case and sensitivity analyses, the changes in HRQoL (utility values and VAS scores) over time were largely consistent.

### Study limitations

First, our study only included patients with HFrEF. A study has shown that patients with HFrEF had better HRQoL compared with HFpEF patients [9], therefore the trajectory of HRQoL for HFrEF patients might not be transferable to patients with HFmrEF and HFpEF and this warrants further study on the differences. Secondly, the risk of sampling bias particularly socioeconomic representation cannot be ruled out because we did not include patients from the higher income groups and attending to private hospitals. This limits the generalisability of findings to all Malaysians with HFrEF. Third, exclusion of patients who passed away before discharge or were critically ill due to difficulty in interviewing them could possibly overestimate the utility values of HFrEF population. Lastly, our study only followed up with the patients for 1 month after discharge. Longer follow-up is useful to observe the changes in HRQoL over time.

## Conclusion

HF is a progressive condition with substantial variation in HRQoL during the disease trajectory. During hospitalisation due to worsening of HF, HFrEF population has unfavourable HRQoL. Rapid and significant HRQoL improvement was observed at discharge, which sustained over 1 month. The change in HRQoL during and after hospitalisation can inform future CEAs and policies.

## Electronic supplementary material

Below is the link to the electronic supplementary material.

Supplementary Material 1

## Data Availability

The data supporting this study’s findings is available from the corresponding author upon reasonable request.
