# Supplementary material for: Trajectory of health-related quality of life during and after hospitalisation due to worsening of heart failure
Source: Qual Life Res. 2024 Oct 30;34(2):471–84. doi: 10.1007/s11136-024-03818-6 (PMC11865137; doi:10.1007/s11136-024-03818-6)
Supplement: Supplementary file 1 — Supplementary Material 1 [file 11136_2024_3818_MOESM1_ESM.docx]

**Online Resource**

**Trajectory of health-related quality of life during and after hospitalisations due to worsening of heart failure**

Wai-Chee Kuan, BPharm^1^, Ka Keat Lim, PhD^2^, Kok Han Chee, MD^3^, Sazzli Kasim, MD^4,5^, Dujaili Juman Abdulelah, PhD^1,6^, Kenneth Kwing-Chin Lee, PhD^7,8^, Siew Li Teoh, PhD^1^

**Table of Content**

[Online Resource 1: Inclusion and exclusion criteria 3](#_Toc179131053)

[Online Resource 2: STROBE checklist 4](#_Toc179131054)

[Online Resource 3: Variables categorisation 6](#_Toc179131055)

[Online Resource 4: Missing data and multiple imputation 7](#_Toc179131056)

[Online Resource 5: LASSO regression variable selection procedure 9](#_Toc179131057)

[Online Resource 6: Generalised Linear Mixed Model (GLMM) 13](#_Toc179131058)

[(A) Variables adjusted for utility model 14](#_Toc179131059)

[(B) Variables adjusted for VAS model 14](#_Toc179131060)

[Online Resource 7: Baseline demographics of study participants 15](#_Toc179131061)

[(A) Comparison among different settings 15](#_Toc179131062)

[(B) Comparison among different datasets 16](#_Toc179131063)

[Online Resource 8: Unadjusted mean utility values of HFrEF patients by subgroups 19](#_Toc179131064)

[Online Resource 9: Univariate analysis of GLMM for utility values 21](#_Toc179131065)

[(A) Univariate analysis using MI datasets 21](#_Toc179131066)

[(B) Univariate analysis using AV datasets 23](#_Toc179131067)

[Online Resource 10: Multivariable analysis using GLMM for utility values 25](#_Toc179131068)

[(A) Model 1: Base-case analysis using MI datasets (LASSO) 25](#_Toc179131069)

[(B) Model 2: Sensitivity analysis using AV datasets (LASSO) 26](#_Toc179131070)

[(C) Model 3: Sensitivity analysis using MI datasets (literature and clinical relevance) 27](#_Toc179131071)

[Online Resource 11: Unadjusted mean VAS score of HFrEF patients by subgroups 28](#_Toc179131072)

[Online Resource 12: Univariate analysis of GLMM for VAS scores 30](#_Toc179131073)

[(A) Univariate analysis using MI datasets 30](#_Toc179131074)

[(B) Univariate analysis using AV datasets 32](#_Toc179131075)

[Online Resource 13: Multivariable analysis using GLMM for VAS scores 34](#_Toc179131076)

[(A) Model 1: Base-case analysis using MI datasets (LASSO) 34](#_Toc179131077)

[(B) Model 2: Sensitivity analysis using AV datasets (LASSO) 35](#_Toc179131078)

[(C) Model 3: Sensitivity analysis using MI datasets (literature and clinical relevance) 36](#_Toc179131079)

[Online Resource 14: Plot density for unadjusted mean utility values and VAS scores 37](#_Toc179131080)

[(A) Unadjusted mean utility values 37](#_Toc179131081)

[(B) Unadjusted mean VAS scores 37](#_Toc179131082)

[(C) Unadjusted mean change in utility values 38](#_Toc179131083)

[(D) Unadjusted mean change in VAS scores 38](#_Toc179131084)

[References 39](#_Toc179131085)

# Online Resource 1: Inclusion and exclusion criteria

| Inclusion criteria |
| --- |
| 1. Patients ≥18 years of age 2. A confirmed diagnosis of heart failure (ICD-10 diagnosis I50.9) and with evidence of left ventricular ejection fraction (LVEF) ≤40% within the past 6 months (by formal echocardiography, Point-of-Care Ultrasound (POCUS), CT scan, MRI or ventricular angiography) and no subsequent documented LVEF >40% 3. Hospitalised for more than 24 hours with any symptoms and signs of fluid overload (including but not limited to dyspnoea, orthopnoea, paroxysmal nocturnal dyspnoea, reduced exercise tolerance and peripheral oedema) and requiring IV diuretics, suggestive of HF worsening |
| Exclusion criteria |
| 1. Patients who are critically ill (e.g. on mechanical ventilation throughout hospitalisation until discharge, end-stage renal failure/severe kidney insufficiency, severe liver cirrhosis, cancer receiving active treatment) 2. Patients with active contagious infective diseases requiring isolation (e.g. MRSA, tuberculosis, Covid-19) 3. Patients who passed away during index hospitalisation 4. Patients who have scheduled major surgery within the 1 month or had a surgery in the past 1 month 5. Patients/caregiver who are unwilling to participant or provide consent 6. Patients/caregiver who are unable to provide consent and/or comprehend (e.g. mentally ill, unconscious, unable to speak) |

# Online Resource 2: STROBE checklist

STROBE Statement—Checklist of items that should be included in reports of ***cohort studies***

|  | Item No | Recommendation | Page |
| --- | --- | --- | --- |
| **Title and abstract** | 1 | (*a*) Indicate the study’s design with a commonly used term in the title or the abstract | Title page |
|  |  | (*b*) Provide in the abstract an informative and balanced summary of what was done and what was found | 1 |
| Introduction | | |  |
| Background/rationale | 2 | Explain the scientific background and rationale for the investigation being reported | 3-4 |
| Objectives | 3 | State specific objectives, including any prespecified hypotheses | 3-4 |
| Methods | | |  |
| Study design | 4 | Present key elements of study design early in the paper | 5-7 |
| Setting | 5 | Describe the setting, locations, and relevant dates, including periods of recruitment, exposure, follow-up, and data collection | 5-7 |
| Participants | 6 | (*a*) Give the eligibility criteria, and the sources and methods of selection of participants. Describe methods of follow-up | 5-7 and Online Resource 1 |
|  |  | (*b*) For matched studies, give matching criteria and number of exposed and unexposed | NA |
| Variables | 7 | Clearly define all outcomes, exposures, predictors, potential confounders, and effect modifiers. Give diagnostic criteria, if applicable | 5-7 and Online Resource 3 |
| Data sources/ measurement | 8* | For each variable of interest, give sources of data and details of methods of assessment (measurement). Describe comparability of assessment methods if there is more than one group | 5-7 |
| Bias | 9 | Describe any efforts to address potential sources of bias | 5-7 |
| Study size | 10 | Explain how the study size was arrived at | 5-7 |
| Quantitative variables | 11 | Explain how quantitative variables were handled in the analyses. If applicable, describe which groupings were chosen and why | 5-7 |
| Statistical methods | 12 | (*a*) Describe all statistical methods, including those used to control for confounding | 5-7 |
|  |  | (*b*) Describe any methods used to examine subgroups and interactions | 5-7 |
|  |  | (*c*) Explain how missing data were addressed | 5-7 |
|  |  | (*d*) If applicable, explain how loss to follow-up was addressed | 5-7 |
|  |  | (*e*) Describe any sensitivity analyses | 5-7 |
| Results | | |  |
| Participants | 13* | (a) Report numbers of individuals at each stage of study—eg numbers potentially eligible, examined for eligibility, confirmed eligible, included in the study, completing follow-up, and analysed | Figure 1 |
|  |  | (b) Give reasons for non-participation at each stage | Figure 1 |
|  |  | (c) Consider use of a flow diagram | Figure 1 |
| Descriptive data | 14* | (a) Give characteristics of study participants (eg demographic, clinical, social) and information on exposures and potential confounders | Table 1 and Online Resource 7 |
|  |  | (b) Indicate number of participants with missing data for each variable of interest | Table 1 and Online Resource 7 |
|  |  | (c) Summarise follow-up time (eg, average and total amount) | 8-9 |
| Outcome data | 15* | Report numbers of outcome events or summary measures over time | Table 2-4  and Online Resource 8 and 12 |
| Main results | 16 | (*a*) Give unadjusted estimates and, if applicable, confounder-adjusted estimates and their precision (eg, 95% confidence interval). Make clear which confounders were adjusted for and why they were included | Table 2-4  and Online Resource 6, 8 and 12 |
|  |  | (*b*) Report category boundaries when continuous variables were categorized | Table 1 and Online Resource 6, 8 and 12 |
|  |  | (*c*) If relevant, consider translating estimates of relative risk into absolute risk for a meaningful time period | NA |
| Other analyses | 17 | Report other analyses done—eg analyses of subgroups and interactions, and sensitivity analyses | Figure 2 and Online Resource 10 and 13 |
| Discussion | | |  |
| Key results | 18 | Summarise key results with reference to study objectives |  |
| Limitations | 19 | Discuss limitations of the study, taking into account sources of potential bias or imprecision. Discuss both direction and magnitude of any potential bias | 10-17 |
| Interpretation | 20 | Give a cautious overall interpretation of results considering objectives, limitations, multiplicity of analyses, results from similar studies, and other relevant evidence | 10-17 |
| Generalisability | 21 | Discuss the generalisability (external validity) of the study results | 10-17 |
| Other information | | |  |
| Funding | 22 | Give the source of funding and the role of the funders for the present study and, if applicable, for the original study on which the present article is based | Title page |

NA: not available

*Give information separately for exposed and unexposed groups.

**Note:** An Explanation and Elaboration article discusses each checklist item and gives methodological background and published examples of transparent reporting. The STROBE checklist is best used in conjunction with this article (freely available on the Web sites of PLoS Medicine at http://www.plosmedicine.org/, Annals of Internal Medicine at http://www.annals.org/, and Epidemiology at http://www.epidem.com/). Information on the STROBE Initiative is available at http://www.strobe-statement.org.

# Online Resource 3: Variables categorisation

The variables collected in the study were age, gender, ethnics (Malay, Chinese and others), language versions of EQ-5D-5L (Malay, Chinese and English), marital status (yes/no), working status (yes/no), educational status (primary, secondary, tertiary level and above), income groups (B40, M40, T20), smoke (non-smoker, former and current smoker), body mass index (BMI), cause of HF (ischaemic/non-ischaemic), denovo HF (newly diagnosed/recurrent HF), year since diagnosis (≤1 year and >1 year), prior HF hospitalisation (yes/no), number of days in the hospital, left ventricular ejection fraction (LVEF) categories (≤10%, 11-20%, 21-30%, 31-40%), NYHA classification (class I, II, III, IV), comorbidities including hypertension, diabetes, dyslipidemia, ischaemic heart disease (IHD), stroke/transient ischaemic attack (TIA), atrial fibrillation (AF), chronic kidney disease (CKD), lung disease (chronic obstructive pulmonary disease and/or asthma), anaemia, prior coronary artery bypass graft (CABG), previous percutaneous coronary intervention (PCI), prior implantable cardioverter-defibrillators (ICD), and prior valve replacement, laboratory measurements such as serum haemoglobin, serum creatinine and estimated glomerular filtration rate (eGFR), systolic blood pressure (SBP) at discharge, pre-admission and discharged medications which included diuretics, angiotensin-converting enzyme inhibitors (ACEI), angiotensin II receptor blockers (ARB), angiotensin receptor-neprilysin inhibitors (ARNI), beta-blockers, mineralocorticoid receptor antagonist (MRA) and sodium-glucose cotransporter 2 inhibitors (SGLT-2i). Comorbidities were summarised using Charlson’s Comorbidity Index (CCI) and CCI ≥ 3 was defined as high comorbidity group due to its predictive ability for mortality and readmission [1].

Charles comorbidity index (CCI): the 19 item-version


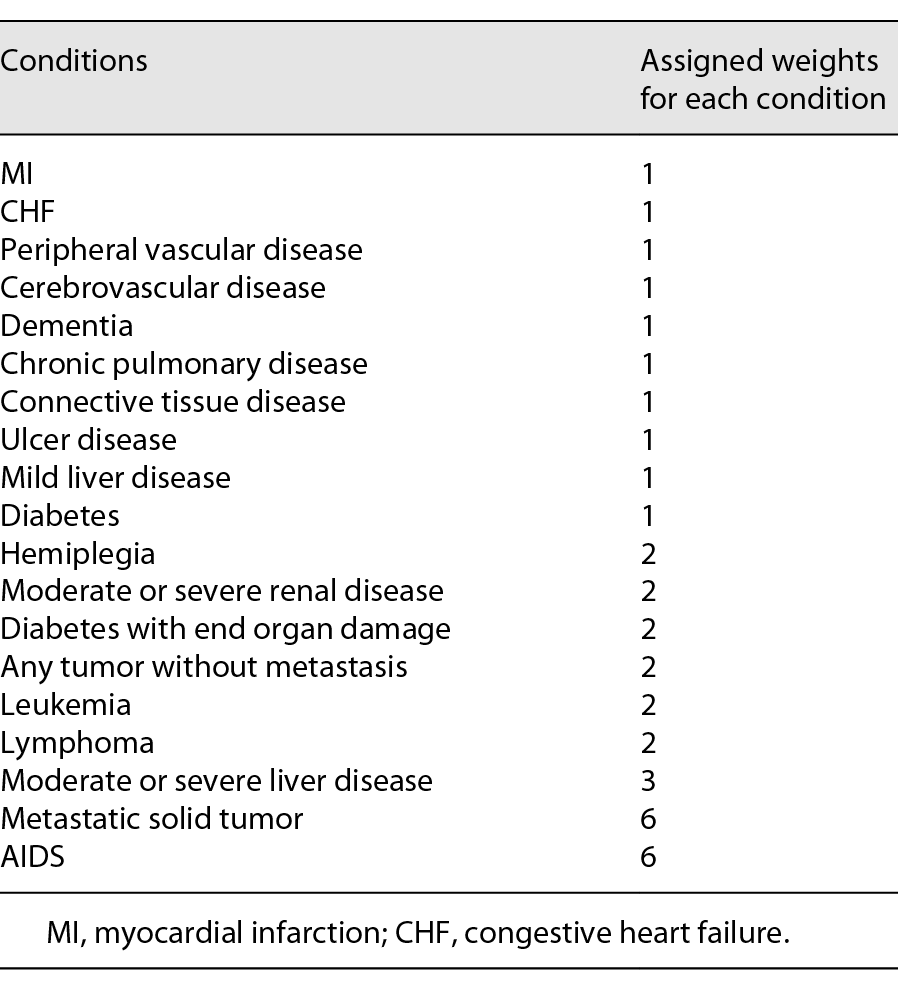


# Online Resource 4: Missing data and multiple imputation

Given that our data included missing covariates and EQ-5D-5L data at 1MPD and multiple imputation (MI) produced unbiased estimates regardless of the percentages of missingness and whether the data were missing at random or completely at random [2], multiple imputation chained equation (MICE) [2,3,4,5] was chosen. The MI was performed using the function in the R ‘mice’ package. As a rule of thumb, the number of imputed data sets should be at least as large as the percentage of missing data[6]. In this study, 50 imputations were performed as per Simons et al. [3]. EQ-5D-5L at 1MPD were imputed by score level because imputation by score level has higher accuracy compared with item level for medium size datasets (n=200) with missing EQ-5D-5L being unit non-response [3]. Table 4A listed the variables included in the multiple imputation. Following imputation, the 50 multiple imputed datasets were pooled using ‘pool’ function that averages the estimates by Rubin’s rules.[van Buuren, #4424]

Rosel et al [7] has shown that MI and all available (AV) datasets have similar performance when utility values were imputed but not covariates. While this study has both missing utility values and covariates (Table 4B), sensitivity analysis was conducted to examine the difference of using MI datasets and AV datasets in the presence of missing covariates. Complete-case analysis (n=173) was not chosen because it consistently produced high EQ-5D-5L scores, overestimating the true EQ-5D-5L scores with increasingly larger mean square errors and standard errors [2]. Besides that, complete-case analysis requires a very strong assumption that data was missing completely at random [5], which is less relevant in this study because patients with worse general health status were more prone to be lost to follow-up and this simulated the mechanism of data missing at random [2].

(A) Variables included in the multiple imputation

| Categories | Variables |
| --- | --- |
| **Demographics** | Age, gender, ethnicity, marital status, education, employment status, income level, body mass index, smoking |
| **Medical history** | De novo HF, ischaemic HF, year since diagnosis, number of hospital days, prior HF hospitalisation |
| **Comorbidities** | Hypertension, Type 2 diabetes, dyslipidemia, IHD, stroke/TIA, atrial fibrillation, CABG, PCI, ICD, valve replacement, liver disease, lung disease (COPD/asthma), anaemia, CCI |
| **HF-related investigations** | LVEF, NYHA at discharge |
| **Laboratory measurements at baseline** | Serum sodium, serum potassium, serum urea, serum creatinine, eGFR, plasma haemoglobin, HbA1c |
| **Vital signs** | Systolic blood pressure at discharge |
| **Pre-admission medications** | Diuretics, ACEI, ARB, ARNI, BB, MRA, SGLT-2i, statin, antiplatelet, anticoagulant, trimetazidine, ivabradine, digoxin, isosorbide nitrates, insulin, ezetimibe |
| **Discharged medications** | Diuretics, ACEI, ARB, ARNI, BB, MRA, SGLT-2i, statin, antiplatelet, anticoagulant, trimetazidine, ivabradine, isosorbide nitrates, digoxin, insulin, ezetimibe |
| **Outcomes** | Utility scores, VAS scores |
| **Others** | EQ-5D-5L language |

ACEI: angiotensin converting enzyme inhibitor; ARB: angiotensin receptor blocker; ARNI: angiotensin receptor neprilysin inhibitor; BB: beta-blocker; BMI: body mass index; SBP: systolic blood pressure; CCI: Charles Comorbidity Index; COPD: chronic obstructive pulmonary disease; CABG: coronary artery bypass graft; CKD: chronic kidney disease; eGFR: estimated glomerular filtration rate; HF: heart failure; IHD: ischemic heart disease, LVEF: left ventricular ejection fraction; MRA: mineralocorticoid receptor antagonist; NYHA: New York Heart Association; PCI: percutaneous coronary intervention; ICD: implantable cardioverter-defibrillator; SGLT-2i: sodium-glucose co-transporter-2 inhibitor; TIA: transient ischaemic attack; VAS: visual analogue scale.

(B) Missing Data pattern, percentage and methods of imputation

| No | Variable | Count (n) | Percentage (%) | Methods of imputation |
| --- | --- | --- | --- | --- |
| 1 | Income level | 5 | 2.5 | Polytomous regression |
| 2 | BMI | 10 | 5.0 | Predictive mean matching |
| 3 | NYHA | 27 | 13.5 | Polytomous regression |
| 4 | HbA1c | 11 | 5.5 | Predictive mean matching |
| 5 | Systolic blood pressure | 2 | 1 | Predictive mean matching |
| 6 | Utility value at 1MPD | 27 | 13.5 | Predictive mean matching |
| 7 | VAS scores at 1MPD | 27 | 13.5 | Predictive mean matching |

# Online Resource 5: LASSO regression variable selection procedure

Given that HRQoL is subjected to many social determinants and population-specific factors [4,8,9] and inclusion of large numbers of predictors through predictor analytic steps in the regression model could potentially lead to overfitting and multicollinearity, we used data-mining method (LASSO regression - a supervised machine learning algorithm) [10,11] to regularise all covariates and identify the important variables that could confound the outcomes to be adjusted for. This also allows us to identify covariates unmeasured in other studies but relevant to our local context.

We used the *train* function under the “caret” package in R to train the 50 sets of imputed data with LASSO regression. We also employed 10-fold cross-validation to minimise the mean square error and increase the model accuracy and interpretability [11]. The variables included in the model for variable selection were listed in Table 5A. The RMSE and R-square were listed in Table 5B. Model with the lowest RMSE and acceptable R-square was chosen as the best model. The top 20 most important variables and their relative importance were listed in Table 5C.

(A) Variables included in LASSO selection procedure:

| **Categories** | **Variables** |
| --- | --- |
| **Demographics** | Age, gender, ethnicity, marital status, education level, employment status, body mass index, smoking |
| **Medical history** | De novo HF, ischaemic HF, year since diagnosis, prior HF hospitalisation, number of hospital days |
| **Comorbidities** | Hypertension, Type 2 diabetes, dyslipidaemia, ischemic heart disease, stroke/TIA, atrial fibrillation, CABG, PCI, ICD, liver disease, CKD, lung disease, CCI |
| **HF-related investigations** | LVEF, NYHA at discharge |
| **Laboratory measurements at baseline** | Serum sodium, serum potassium, serum urea, serum creatinine, eGFR, plasma haemoglobin, HbA1c |
| **Vital signs** | Systolic blood pressure at discharge |
| **Pre-admission medications** | Diuretics, ACEI, ARB, ARNI, BB, MRA, SGLT-2i, statin, antiplatelet, anticoagulant, trimetazidine, ivabradine, insulin, ezetimibe |
| **Discharged medications** | Diuretics, ACEI, ARB, ARNI, BB, MRA, SGLT-2i, statin, antiplatelet, anticoagulant, trimetazidine, ivabradine, digoxin, insulin, ezetimibe |
| **Outcomes** | Utility values |
| **Others** | EQ-5D-5L language |
| **Interactions** | Age*NYHA  Age*CCI≥3  Age* year since diagnosis  Age* prior HF hospitalisation  Age*number of hospital days  Work*NYHA |

Variables with near zero variance (i.e. income level, valve replacement, pre-admission digoxin, pre-admission isosorbide nitrates and discharged isosorbide nitrates) were removed from LASSO selection procedure.

(B) RMSE and R-squared for all 50 imputation sets

| Utility | Model | RMSE | R-square |  | VAS | Model | RMSE | R-square |
| --- | --- | --- | --- | --- | --- | --- | --- | --- |
| 1 | 3 | 0.3178 | 0.5003 |  | **1** | 6 | 19.23 | 0.4146 |
| 2 | 25 | 0.3195 | 0.491 |  | **2** | 41 | 19.4 | 0.4059 |
| 3 | 10 | 0.3199 | 0.4923 |  | **3** | 26 | 19.43 | 0.3925 |
| 4 | 24 | 0.3201 | 0.4941 |  | **4** | 46 | 19.47 | 0.3993 |
| 5 | 4 | 0.3207 | 0.498 |  | **5** | 23 | 19.47 | 0.4023 |
| 6 | 30 | 0.3209 | 0.4861 |  | **6** | 3 | 19.47 | 0.3984 |
| 7 | 46 | 0.3214 | 0.4952 |  | **7** | 40 | 19.5 | 0.3966 |
| 8 | 45 | 0.3216 | 0.4919 |  | **8** | 4 | 19.52 | 0.3952 |
| 9 | 29 | 0.3217 | 0.4874 |  | **9** | 30 | 19.53 | 0.3815 |
| 10 | 23 | 0.322 | 0.4882 |  | **10** | 8 | 19.54 | 0.3915 |
| 11 | 42 | 0.3223 | 0.4874 |  | **11** | 43 | 19.56 | 0.3882 |
| 12 | 17 | 0.3225 | 0.4901 |  | **12** | 49 | 19.57 | 0.3923 |
| 13 | 49 | 0.3225 | 0.4755 |  | **13** | 19 | 19.58 | 0.3879 |
| 14 | 31 | 0.3227 | 0.4821 |  | **14** | 37 | 19.58 | 0.3916 |
| 15 | 19 | 0.3227 | 0.4903 |  | **15** | 18 | 19.58 | 0.403 |
| 16 | 26 | 0.3228 | 0.4794 |  | **16** | 25 | 19.59 | 0.3906 |
| 17 | 37 | 0.3229 | 0.4823 |  | **17** | 44 | 19.63 | 0.4014 |
| 18 | 34 | 0.3231 | 0.4824 |  | **18** | 35 | 19.64 | 0.3865 |
| 19 | 43 | 0.3235 | 0.479 |  | **19** | 15 | 19.65 | 0.3917 |
| 20 | 8 | 0.3235 | 0.4953 |  | **20** | 16 | 19.65 | 0.3835 |
| 21 | 16 | 0.3237 | 0.4807 |  | **21** | 17 | 19.67 | 0.3865 |
| 22 | 13 | 0.3245 | 0.4702 |  | **22** | 9 | 19.67 | 0.3922 |
| 23 | 5 | 0.3249 | 0.4795 |  | **23** | 12 | 19.69 | 0.4005 |
| 24 | 44 | 0.3257 | 0.482 |  | **24** | 2 | 19.72 | 0.3959 |
| 25 | 39 | 0.3258 | 0.4776 |  | **25** | 33 | 19.73 | 0.3933 |
| 26 | 28 | 0.3259 | 0.4666 |  | **26** | 29 | 19.73 | 0.3822 |
| 27 | 50 | 0.3261 | 0.4701 |  | **27** | 34 | 19.74 | 0.3844 |
| 28 | 12 | 0.3262 | 0.4852 |  | **28** | 47 | 19.74 | 0.3774 |
| 29 | 41 | 0.3263 | 0.4793 |  | **29** | 32 | 19.75 | 0.3882 |
| 30 | 40 | 0.3268 | 0.467 |  | **30** | 38 | 19.76 | 0.38 |
| 31 | 9 | 0.3268 | 0.4759 |  | **31** | 13 | 19.78 | 0.3823 |
| 32 | 7 | 0.3269 | 0.4741 |  | **32** | 5 | 19.78 | 0.3856 |
| 33 | 11 | 0.3271 | 0.4764 |  | **33** | 31 | 19.79 | 0.387 |
| 34 | 32 | 0.3271 | 0.4689 |  | **34** | 45 | 19.8 | 0.3832 |
| 35 | 35 | 0.3272 | 0.475 |  | **35** | 10 | 19.8 | 0.3861 |
| 36 | 33 | 0.3276 | 0.4631 |  | **36** | 22 | 19.82 | 0.3848 |
| 37 | 38 | 0.3276 | 0.4674 |  | **37** | 7 | 19.87 | 0.3851 |
| 38 | 48 | 0.3277 | 0.4676 |  | **38** | 21 | 19.88 | 0.3788 |
| 39 | 2 | 0.3277 | 0.4729 |  | **39** | 50 | 19.91 | 0.3874 |
| 40 | 14 | 0.3281 | 0.4752 |  | **40** | 39 | 19.91 | 0.3805 |
| 41 | 36 | 0.3282 | 0.4766 |  | **41** | 14 | 19.91 | 0.3754 |
| 42 | 27 | 0.3284 | 0.4671 |  | **42** | 28 | 19.94 | 0.3709 |
| 43 | 18 | 0.3285 | 0.4648 |  | **43** | 11 | 19.95 | 0.3831 |
| 44 | 15 | 0.3286 | 0.4669 |  | **44** | 24 | 19.98 | 0.3876 |
| 45 | 1 | 0.3286 | 0.4831 |  | **45** | 48 | 19.99 | 0.3704 |
| 46 | 20 | 0.3287 | 0.4759 |  | **46** | 27 | 20.02 | 0.3723 |
| 47 | 47 | 0.3295 | 0.4566 |  | **47** | 20 | 20.04 | 0.373 |
| 48 | 21 | 0.3301 | 0.468 |  | **48** | 42 | 20.07 | 0.3705 |
| 49 | 6 | 0.3311 | 0.454 |  | **49** | 1 | 20.14 | 0.3647 |
| 50 | 22 | 0.3316 | 0.4633 |  | **50** | 36 | 20.23 | 0.3577 |

Model 3 with the lowest RMSE and acceptable was selected for utility model whereas

(C) The top 20 most important variables and percentage importance

| **Utility model** | **Percentage importance** |  | **VAS model** | **Percentage importance** |
| --- | --- | --- | --- | --- |
| **Time: 1MPD** | 100 |  | **Time: 1MPD** | 100 |
| **Time: Discharge** | 94.58 |  | **Time: Discharge** | 92.56 |
| **NYHA 4** | 75.11 |  | **NYHA 4** | 43.85 |
| **Age*NYHA 3** | 23.63 |  | **Age*NYHA 2** | 32.5 |
| **NYHA 2** | 21.07 |  | **Age*NYHA 3** | 26.57 |
| **SBP at discharge** | 14.43 |  | **eGFR** | 21.93 |
| **NYHA 3** | 13.64 |  | **Recurrent HF** | 18.27 |
| **Pre-admission ezetimibe** | 11.96 |  | **Discharged ARB** | 15.72 |
| **Working** | 9.74 |  | **Pre-admission ARNI** | 15.01 |
| **Hypertension** | 8.93 |  | **Anaemia** | 14.3 |
| **Female** | 8.82 |  | **Serum urea** | 12.63 |
| **Serum sodium** | 8.53 |  | **CCI≥3** | 12 |
| **Current Smoker** | 7.75 |  | **Discharged statin** | 11.25 |
| **Pre-admission insulin** | 6.01 |  | **EQ-5D-5L Language: English** | 11.02 |
| **Discharged insulin** | 5.53 |  | **Serum sodium** | 11 |
| **Pre-admission anti-coagulants** | 5.31 |  | **LVEF** | 9.83 |
| **Age*Hospital days** | 4.08 |  | **Pre-admission SGLT-2i** | 9.72 |
| **EQ-5D-5L Language: English** | 4.04 |  | **Pre-admission trimetazidine** | 9.56 |
| **EQ-5D-5L Language: Chinese** | 3.72 |  | **Education: Tertiary** | 9.49 |
| **Education: Tertiary** | 3.71 |  | **Lung disease** | 9.11 |

# Online Resource 6: Generalised Linear Mixed Model (GLMM)

When examining EQ-5D-5L data, the common statistical considerations were ceiling effects and distributional issues [12,13]. To address these issues, different methods have been proposed, including Tobit, censored least absolute deviations, ordinal least square (OLS) linear regression, two-part, generalised linear model (GLM), and beta regression (Breg) models [9,14]. It seems that no single model can address all concerns and issues related to utility data. Estimation using Tobit or censored least absolute deviations could be affected by their censoring assumptions [15] while OLS model assumptions may be violated by the ceiling effect and bounded properties of utility data. Berg et al [9], on the other hand, reported that two-part model performed poorer than the OLS model due to its lower consistency in capturing the range of observed utilities. Martín-Fernández et al [14] found that generalised linear mixed model (GLMM) and beta regression produced the lowest mean absolute error (MAE) and were the most accurate methods for predicting utility values.

Given that the primary concern of this study was the consistent estimation of changes in utility values and VAS scores before and after hospitalisation considering the potential bias arising from within and between-patient variability, instead of the distributional form [16], GLMM was chosen to estimate the to account for the variability among patients within a health state and variability over time within a single health state like previous studies [4,8,17]. Its relative accuracy in predicting the population mean and ease of implementation also justified the choice of GLMM [9]. Table 6A summarised the variables adjusted for utility model whereas Table 6B is summarised the variables adjusted for VAS model.

## (A) Variables adjusted for utility model

| Utility Model | Base-case analysis  (LASSO) | Sensitivity analysis  (literature and clinical relevance) |
| --- | --- | --- |
| Fixed effect | - Age, gender, EQ-5D-5L language, educational level, work, smoke, number of hospital stay, NYHA, hypertension, serum sodium, systolic blood pressure (SBP) at discharge, pre-admission insulin, pre-admission ezetimibe, pre-admission anticoagulants, discharged insulin, and time of measurements, interaction between age and NYHA, interaction between age and number of hospital stay | - Age, gender, ethnics, EQ-5D-5L language, work, married, educational level, number of hospital stay, NYHA, CCI, serum haemoglobin level, eGFR, pre-admission ivabradine, pre-admission insulin, discharged ARNI, discharged beta-blockers, time of measurements, interaction between age and NYHA. |
| Random effect | - Within patient correlation | - Within patient correlation |

Other potential variables not adjusted for in this study and could be potential confounders included income level, living alone, HF duration, previous HF admission in the preceding year, aetiology, NT-pro-BNP, heart rate, BMI, potassium, MRA, SGLT-2i, digoxin, nitrates, diuretic, anti-platelet, revascularisation, device, myocardial infarction (MI), dilated cardiomyopathy, valve disease, valve surgery, atrial arrhythmia, diabetes, lung disease[4,18]. Most variables have been included in the LASSO variable selection except NT-pro-BNP, income level, valve replacement, digoxin, nitrates due to zero variance. Therefore, these are the potential confounders.

## (B) Variables adjusted for VAS model

| VAS Model | Base-case analysis  (LASSO) | Sensitivity analysis  (literature and clinical relevance) |
| --- | --- | --- |
| Fixed effect | - Age, EQ-5D-5L language, educational level, recurrent HF, LVEF, NYHA, CCI, lung disease (COPD/asthma), anaemia, serum sodium level, serum urea level, eGFR, pre-admission ARNI, pre-admission SGLT-2i, pre-admission trimetazidine, discharged statin, discharged ARB, time of measurements, interaction between age and NYHA. | - Age, gender, ethnics, EQ-5D-5L language, work, NYHA, CCI, IHD, serum haemoglobin level, pre-admission anti-platelet, discharged ARNI, discharged beta-blockers, time of measurements. |
| Random effect | - Within patient correlation | - Within patient correlation |

Other potential variables not adjusted for in this study and could be potential confounders included income level, living alone, HF duration, previous HF admission in the preceding year, aetiology, NT-pro-BNP, systolic blood pressure, heart rate, BMI, potassium, MRA, SGLT-2i, digoxin, nitrates, diuretic, anti-coagulant, revascularisation, device, smoking, myocardial infarction (MI), dilated cardiomyopathy, valve disease, valve surgery, atrial arrhythmia, hypertension, diabetes. [18,19] Most variables have been included in the LASSO variable selection except NT-pro-BNP, income level, valve replacement, digoxin, nitrates due to zero variance. Therefore, these are the potential confounders.

# Online Resource 7: Baseline demographics of study participants

## (A) Comparison among different settings

| Variables | Our study  (n=200) | IJN Registry  (n=4496) | MY-HF registry (n=2717) [20] |
| --- | --- | --- | --- |
| HFrEF, n (%) | 200 (100) | 4496 (100) | 1737 (63.9) |
| Mean age (SD) | 61.4 (13.7) | 61.5 (13.6) | 60.2 (13.6) |
| ≥60 years, n (%) | 121 (60.5) | 102 (59.0) | 1204 (55.7) |
| Male (%) | 148 (74.0) | 130 (75.1) | 1814 (66.8) |
| Ethnicity (%) |  |  |  |
| Malay | 110 (55.0) | 98 (56.6) | 1,609 (59.3) |
| Chinese | 56 (28.0) | 50 (28.9) | 543 (20.0) |
| Indian | 33 (16.5) | 24 (13.9) | 444 (17.0) |
| Others | 1 (0.5) | 1 (0.6) | 121 (4.4) |
| Current/Previous smoker, n (%) | 101 (51.0) | 1954 (47.9) | 1,274 (46.9) |
| Ischaemic aetiology, n (%) | 133 (66.5) | 3122 (69.4) | 1717 (63.2) |
| Prior HF hospitalisation, n (%) | 119 (59.5) | 2435 (54.2) | 1610 (59.2) |
| Comorbidities, n (%) |  |  |  |
| Hypertension | 130 (65.0) | 3194 (71.0) | 1943 (71.5) |
| Type 2 Diabetes | 128 (64.0) | 2831 (63.0) | 1,625 (59.8) |
| Dyslipidemia | 73 (36.5) | 1711 (38.1) | 1266 (46.6) |
| Prior ischemic heart disease | 118 (59.0) | 3244 (72.2) | 1519 (55.9) |
| Prior stroke / TIA | 23 (11.5) | 235 (5.2) | 103 (3.8) |
| Atrial fibrillation | 49 (24.5) | 843 (18.8) | 375 (13.8) |
| Prior device implantation | 12 (6.0) | 493 (11.0) | 66 (9.8) |

HFrEF: heart failure with reduced ejection fraction; SD: standard deviation; HF: heart failure; TIA: transient ischaemic attack.

## (B) Comparison among different datasets

| Variables | AV datasets  (N=200)^†^ | MI datasets  (N=200)^††^ | Complete data at 1MPD (N=173)^‡^ | Missing data at 1MPD (N=27)^‡‡^ |
| --- | --- | --- | --- | --- |
| Mean age (SD) | 61.4 (13.7) | 61.4 (13.7) | 61.3 (13.8) | 61.7 (12.8) |
| ≥60 years, n (%) | 121 (60.5) | 121 (60.5) | 102 (59.0) | 19 (70.4) |
| Gender, n (%) |  |  |  |  |
| Male | 148 (74.0) | 148 (74.0) | 130 (75.1) | 18 (66.7) |
| Female | 52 (26.0) | 52 (26.0) | 43 (24.9) | 9 (33.3) |
| Ethnicity, n (%) |  |  |  |  |
| Malay | 110 (55.0) | 110 (55.0) | 98 (56.6) | 12 (44.4) |
| Chinese | 56 (28.0) | 56 (28.0) | 50 (28.9) | 6 (22.2) |
| Indian | 33 (16.5) | 33 (16.5) | 24 (13.9) | 9 (33.3) |
| Others | 1 (0.5) | 1 (0.5) | 1 (0.6) | 0 (0) |
| Married, n (%) |  |  |  |  |
| No | 30 (15.0) | 30 (15.0) | 25 (14.5) | 5 (18.5) |
| Yes | 170 (85.0) | 170 (85.0) | 148 (85.5) | 22 (81.5) |
| Working (n, %) |  |  |  |  |
| No | 147 (73.5) | 147 (73.5) | 126 (72.8) | 21 (77.8) |
| Yes | 53 (26.5) | 53 (26.5) | 47 (27.2) | 6 (22.2) |
| Income group (n, %)^§^ |  |  |  |  |
| B40 (<USD1017) | 185 (92.5) | 185 (92.5) | 161 (93.1) | 24 (88.9) |
| M40 (USD1017-USD2299) | 4 (2.0) | 4 (2.0) | 3 (1.7) | 1 (3.7) |
| T20 (≥USD2299) | 6 (3.0) | 6 (3.0) | 5 (2.9) | 1 (3.7) |
| Unknown | 5 (2.5) |  | 4 (2.3) | 1 (3.7) |
| Educational level (n, %) |  |  |  |  |
| No formal education | 12 (6.0) | 12 (6.0) | 9 (5.2) | 3 (11.1) |
| Primary level | 43 (21.5) | 43 (21.5) | 35 (20.2) | 8 (29.6) |
| Secondary level | 109 (54.5) | 109 (54.5) | 97 (56.1) | 12 (44.4) |
| Tertiary level and above | 36 (18.0) | 36 (18.0) | 32 (18.5) | 4 (14.8) |
| Smoking (n, %) |  |  |  |  |
| Non-smoker | 99 (49.5) | 99 (49.5) | 83 (48.0) | 16 (59.3) |
| Current active smoker | 42 (21.0) | 42 (21.0) | 37 (21.4) | 5 (18.5) |
| Ex-smoker | 59 (29.5) | 59 (29.5) | 53 (30.6) | 6 (22.2) |
| Median BMI, kg/m^2^ (IQR) | 25.1 (22.2, 29.4) | 25.2 (22.3, 29.5) | 25.7 (22.5, 29.4) | 24.2 (21.9, 29.5) |
| Primary cause of heart failure, n (%) |  |  |  |  |
| Non-ischaemic | 67 (33.5) | 67 (33.5) | 60 (34.7) | 7 (25.9) |
| Ischaemic | 133 (66.5) | 133 (66.5) | 113 (65.3) | 20 (74.1) |
| De Novo HF, n (%) | 54 (27.0) | 54 (27.0) | 50 (28.9) | 4 (14.8) |
| Years since HF diagnosis <1 year, n (%) | 125 (62.5) | 125 (62.5) | 110 (63.6) | 15 (55.6) |
| Prior HF hospitalisation, n (%) | 119 (59.5) | 119 (59.5) | 99 (57.3) | 20 (74.1) |
| Median length of hospital stay, days (IQR) | 4.0 (3.0, 6.3) | 4.0 (3.0, 6.3) | 4.0 (3.0, 6.0) | 4.0 (3.0, 8.0) |
| Mean CCI, scores (SD) | 3.7 (1.7) | 3.7 (1.7) | 3.6 (1.7) | 4.0 (1.7) |
| CCI scores > 3 | 102 (51.0) | 102 (51.0) | 86 (49.7) | 20 (74.1) |
| Comorbidities, n (%) |  |  |  |  |
| Hypertension | 130 (65.0) | 130 (65.0) | 114 (65.9) | 16 (59.3) |
| Type 2 Diabetes | 128 (64.0) | 128 (64.0) | 111 (64.2) | 17 (63.0) |
| Dyslipidemia | 73 (36.5) | 73 (36.5) | 63 (36.4) | 10 (37.0) |
| Prior ischemic heart disease | 118 (59.0) | 118 (59.0) | 99 (57.2) | 19 (70.4) |
| Prior stroke / TIA | 23 (11.5) | 23 (11.5) | 19 (11.0) | 4 (14.8) |
| Atrial fibrillation | 49 (24.5) | 49 (24.5) | 41 (23.7) | 8 (29.6) |
| Prior CABG | 15 (7.5) | 15 (7.5) | 13 (7.5) | 2 (7.4) |
| Prior PCI | 41 (20.5) | 41 (20.5) | 35 (20.2) | 6 (22.2) |
| Prior device implantation | 12 (6.0) | 12 (6.0) | 11 (6.4) | 1 (3.7) |
| Prior valve replacement | 9 (4.5) | 9 (4.5) | 9 (5.2) | 0 (0) |
| Chronic kidney disease | 60 (30.0) | 60 (30.0) | 52 (30.1) | 8 (29.6) |
| Liver diseases | 23 (11.5) | 23 (11.5) | 21 (12.1) | 2 (7.4) |
| Lung diseases (COPD / Asthma) | 17 (8.5) | 17 (8.5) | 16 (9.2) | 1 (3.7) |
| Anaemia | 103 (51.5) | 103 (51.5) | 89 (51.4) | 14 (51.9) |
| Mean LVEF, % (SD) | 26.6 (7.6) | 26.6 (7.6) | 26.7 (7.6) | 25.8 (7.9) |
| Baseline laboratory level, at admission |  |  |  |  |
| Hyponatremia (<135mmol/L), n (%) | 46 (23.0) | 46 (23.0) | 41 (23.7) | 5 (18.5) |
| Hyperkalemia (>5.5mmol/L), n (%) | 11 (5.5) | 11 (5.5) | 9 (5.2) | 2 (7.4) |
| Median serum sodium, mmol/l (SD) | 138 (135, 140) | 138 (135, 140) | 138 (135, 140) | 138 (135, 141) |
| Mean serum potassium, mmol/l (SD) | 4.4 (0.6) | 4.4 (0.6) | 4.4 (0.6) | 4.4 (0.8) |
| Median serum urea, mmol/l (SD) | 7.7 (5.7, 11.7) | 7.7 (5.7, 11.7) | 7.4 (5.7, 11.0) | 8.5 (6.9, 13.5) |
| Median serum creatinine, umol/l (IQR) | 108.0 (85.8, 151.8) | 108.0 (85.8, 151.8) | 107.0 (85.0, 145.0) | 136.0 (86.5, 183.5) |
| Median eGFR, ml/min/1.73m^2^ (IQR) | 61 (39.8, 81.0) | 61 (39.8, 81.0) | 62 (42.0, 84.0) | 52 (31.5, 76.0) |
| Median HbA1c, g/dL (IQR) | 7.0 (5.9, 8.1) | 7.0 (5.9, 8.1) | 6.8 (5.9, 8.1) | 7.3 (5.9, 7.8) |
| Mean haemoglobin, g/dL (SD) | 12.8 (2.2) | 12.8 (2.2) | 12.8 (2.2) | 13.0 (2.4) |
| Mean SBP at discharge, mmHg (SD) | 121 (18) | 121 (18) | 122 (18) | 117 (20) |
| Pre-admission medication, n (%) |  |  |  |  |
| Diuretics | 115 (57.5) | 115 (57.5) | 101 (58.4) | 14 (51.9) |
| ACEI | 60 (30.0) | 60 (30.0) | 55 (31.8) | 5 (18.5) |
| ARB | 26 (13.0) | 26 (13.0) | 22 (12.7) | 4 (14.8) |
| ARNI | 47 (23.5) | 47 (23.5) | 39 (22.5) | 8 (29.6) |
| Beta-blocker | 143 (71.5) | 143 (71.5) | 122 (70.5) | 21 (77.8) |
| MRA | 88 (44.0) | 88 (44.0) | 75 (43.4) | 13 (48.1) |
| SGLT-2i | 87 (43.5) | 87 (43.5) | 74 (42.8) | 13 (48.1) |
| Statin | 136 (68.0) | 136 (68.0) | 118 (68.2) | 18 (66.7) |
| Ezetimibe | 22 (11.0) | 22 (11.0) | 17 (9.8) | 5 (18.5) |
| Antiplatelet | 125 (62.5) | 125 (62.5) | 108 (62.4) | 17 (63.0) |
| Anticoagulant | 32 (16.0) | 32 (16.0) | 27 (15.6) | 5 (18.5) |
| Nitrates | 6 (3.0) | 6 (3.0) | 6 (3.5) | 0 (0) |
| Digoxin | 6 (3.0) | 6 (3.0) | 4 (2.3) | 2 (7.4) |
| Insulin | 41 (20.5) | 41 (20.5) | 36 (20.8) | 5 (18.5) |
| Discharged medication, n (%) |  |  |  |  |
| Diuretics | 155 (77.5) | 155 (77.5) | 137 (79.2) | 18 (66.7) |
| ACEI | 41 (20.5) | 41 (20.5) | 37 (21.4) | 4 (14.8) |
| ARB | 20 (10.0) | 20 (10.0) | 17 (9.8) | 3 (11.1) |
| ARNI | 81 (40.5) | 81 (40.5) | 71 (41.0) | 10 (37.0) |
| Beta-blocker | 178 (89.0) | 178 (89.0) | 154 (89.0) | 24 (89.0) |
| MRA | 157 (78.5) | 157 (78.5) | 135 (78.0) | 22 (81.5) |
| SGLT-2i | 146 (73.0) | 146 (73.0) | 128(74.0) | 18 (66.7) |
| Statin | 151 (75.5) | 151 (75.5) | 131 (75.7) | 20 (74.1) |
| Ezetimibe | 29 (14.5) | 29 (14.5) | 23 (13.3) | 6 (22.2) |
| Antiplatelet | 147 (73.5) | 147 (73.5) | 129 (74.6) | 18 (66.7) |
| Anticoagulant | 49 (24.5) | 49 (24.5) | 43 (24.9) | 6 (22.2) |
| Nitrates | 8 (4.0) | 8 (4.0) | 8 (4.6) | 0 (0) |
| Digoxin | 11 (5.5) | 11 (5.5) | 8 (4.6) | 3 (11.1) |
| Insulin | 41 (20.5) | 41 (20.5) | 35 (20.2) | 6 (22.2) |
| NYHA at 1-month post-discharge, n (%) |  |  |  |  |
| Class I | 72 (36.0) | 76 (37.8) | 72 (41.6) |  |
| Class II | 55 (27.5) | 61 (30.7) | 55 (31.8) |  |
| Class III | 20 (10.0) | 28 (13.9) | 20 (11.6) |  |
| Class IV | 26 (13.0) | 35 (17.6) | 26 (15.0) |  |
| Unknown | 27 (13.5) |  |  | 27 (100.0) |
| Unadjusted mean utility value (SD) |  |  |  |  |
| At admission | 0.150 (0.392) | 0.150 (0.392) | 0.174 (0.393) | -0.003 (0.362) |
| At discharge | 0.659 (0.342) | 0.659 (0.342) | 0.690 (0.327) | 0.464 (0.381) |
| At 1-month post-discharge | 0.712 (0.363) | 0.684 (0.379) | 0.712 (0.363) |  |
| Unadjusted mean VAS score (SD) |  |  |  |  |
| At admission | 38.2 (20.8) | 38.2 (20.8) | 38.8 (21.0) | 34.3 (19.5) |
| At discharge | 67.0 (19.2) | 67.0 (19.2) | 67.7 (18.4) | 62.4 (23.4) |
| At 1-month post-discharge | 70.1 (20.6) | 68.9 (22.0) | 70.1 (20.6) |  |

AV: all available; MI: multiple imputed.

^†^Baseline demographics for all 200 patients (missing data were reported as unknown or omitted)

^††^Baseline demographics for all 200 patients (missing data were imputed using multiple imputation)

^‡^Baseline demographics for 173 patients who were successfully followed up at 1MPD

^‡‡^Baseline demographics for 27 patients who were lost to follow-up at 1MPD
Data presented in n (%) unless otherwise stated. 1MPD, 1-month post-discharge; ACEI, angiotensin converting enzyme inhibitor; ARB, angiotensin receptor blocker; ARNI, angiotensin receptor neprilysin inhibitor; BMI, body mass index; CCI: Charles Comorbidity Index; COPD, chronic obstructive pulmonary disease; eGFR, estimated glomerular filtration rate; HF, heart failure; IQR, Interquartile range; LVEF, left ventricular ejection fraction; MRA, mineralocorticoid receptor antagonist; n, number of patients; N, total number of patients; NYHA, New York Heart Association; SGLT-2i, sodium-glucose co-transporter-2 inhibitor; SD, standard deviation; SBP, systolic blood pressure; TIA, transient ischaemic attack; VAS: visual analogue scale. eGFR was calculated based on MDRD equation.

^§^In Malaysia, the monthly income level was categorised into B40 (<RM4850), M40 (RM4850-RM10959) and T20 (≥RM10960). USD was presented in the table, with the exchange rate of MYR1 = USD 0.21 as of 19/10/2023.

# Online Resource 8: Unadjusted mean utility values of HFrEF patients by subgroups

| Mean utility values (SD) | | | At admission (n=200) | p-value^†^ | At discharge (n=200) | p-value^†^ | At 1MPD (n=200) | p-value^†^ | At 1MPD (n=173) | p-value^†^ |
| --- | --- | --- | --- | --- | --- | --- | --- | --- | --- | --- |
| All patients | | | 0.150 (0.393) |  | 0.659 (0.343) |  | 0.684 (0.379) |  | 0.712 (0.363) |  |
| Age < 60 (n =121) | | | 0.159 (0.394) | 0.792 | 0.714 (0.297) | 0.056 | 0.785 (0.335) | **0.003** | 0.823 (0.298) | **<0.001** |
| Age ≥ 60 (n =79) | | | 0.144 (0.394) |  | 0.623 (0.366) |  | 0.618 (0.393) |  | 0.635 (0.384) |  |
| Male (n =148) | | | 0.168 (0.409) | 0.219 | 0.683 (0.334) | 0.108 | 0.712 (0.374) | 0.093 | 0.745 (0.353) | **0.045** |
| Female (n =52) | | | 0.097 (0.34) |  | 0.591 (0.36) |  | 0.603 (0.385) |  | 0.612 (0.377) |  |
| Malay (n =110) | | | 0.141 (0.388) | 0.198 | 0.655 (0.331) | 0.427 | 0.718 (0.355) | 0.149 | 0.744 (0.334) | 0.259 |
| Chinese (n =56) | | | 0.222 (0.384) |  | 0.72 (0.283) |  | 0.699 (0.359) |  | 0.714 (0.341) |  |
| Indian (n =33) | | | 0.050 (0.413) |  | 0.561 (0.446) |  | 0.548 (0.461) |  | 0.563 (0.484) |  |
| Others (n=1) | | | 0.386 |  | 0.952 |  | 1 |  | 1 |  |
| Not married (n =30) | | | 0.155 (0.387) | 0.934 | 0.780 (0.239) | **0.008** | 0.875 (0.253) | **0.004** | 0.884 (0.257) | **0.002** |
| Married (n =170) | | | 0.149 (0.395) |  | 0.638 (0.354) |  | 0.650 (0.388) |  | 0.683 (0.37) |  |
| Not working (n =147) | | | 0.093 (0.381) | **<0.001** | 0.606 (0.368) | **<0.001** | 0.633 (0.392) | **0.002** | 0.655 (0.383) | **<0.001** |
| Working (n =53) | | | 0.306 (0.386) |  | 0.806 (0.196) |  | 0.824 (0.303) |  | 0.866 (0.243) |  |
| Income level B40 (n =185) | | | 0.140 (0.391) | 0.508 | 0.655 (0.351) | 0.633 | 0.676 (0.386) | 0.604 | 0.704 (0.371) | 0.980 |
| Income level M40 (n =4) | | | 0.055 (0.195) |  | 0.678 (0.157) |  | 0.800 (0.22) |  | 0.856 (0.021) |  |
| Income level T20 (n =6) | | | 0.361 (0.477) |  | 0.819 (0.131) |  | 0.766 (0.249) |  | 0.843 (0.069) |  |
| Primary school and below (n =55) | | | 0.145 (0.404) | 0.894 | 0.625 (0.384) | 0.358 | 0.632 (0.411) | **0.009** | 0.683 (0.371) | **0.005** |
| Secondary school (n =109) | | | 0.148 (0.407) |  | 0.635 (0.358) |  | 0.653 (0.38) |  | 0.678 (0.375) |  |
| High school and above (n =36) | | | 0.163 (0.339) |  | 0.783 (0.16) |  | 0.857 (0.268) |  | 0.856 (0.278) |  |
| Non-smoker (n =99) | | | 0.180 (0.411) | 0.202 | 0.646 (0.352) | 0.882 | 0.669 (0.379) | 0.306 | 0.695 (0.36) | 0.268 |
| Active (n =42) | | | 0.190 (0.397) |  | 0.667 (0.34) |  | 0.632 (0.418) |  | 0.664 (0.407) |  |
| Ex-smoker (n =59) | | | 0.071 (0.352) |  | 0.676 (0.333) |  | 0.746 (0.347) |  | 0.771 (0.333) |  |
| Underweight (n =9) | | | 0.133 (0.266) | 0.196 | 0.634 (0.282) | 0.381 | 0.697 (0.268) | 0.650 | 0.624 (0.236) | 0.095 |
| Normal BMI (n =47) | | | 0.201 (0.379) |  | 0.627 (0.349) |  | 0.622 (0.419) |  | 0.648 (0.41) |  |
| Overweight (n =62) | | | 0.071 (0.399) |  | 0.656 (0.344) |  | 0.689 (0.364) |  | 0.725 (0.336) |  |
| Obese (n =71) | | | 0.191 (0.401) |  | 0.702 (0.335) |  | 0.718 (0.377) |  | 0.753 (0.363) |  |
| Non-ischaemic (n =67) | | | 0.168 (0.373) | 0.631 | 0.708 (0.316) | 0.139 | 0.712 (0.367) | 0.469 | 0.722 (0.361) | 0.789 |
| Ischaemic (n =133) | | | 0.14 (0.404) |  | 0.635 (0.354) |  | 0.669 (0.386) |  | 0.706 (0.365) |  |
| De novo HF (n =54) | | | 0.185 (0.389) | 0.444 | 0.69 (0.344) | 0.445 | 0.766 (0.354) | 0.070 | 0.801 (0.323) | **0.030** |
| Recurrent HF (n =146) | | | 0.137 (0.395) |  | 0.648 (0.342) |  | 0.653 (0.385) |  | 0.676 (0.373) |  |
| Year since diagnosis <1 year (n =125) | | | 0.144 (0.374) | 0.782 | 0.67 (0.329) | 0.578 | 0.684 (0.385) | 0.995 | 0.713 (0.367) | 0.942 |
| Year since diagnosis ≥ 1 year (n =75) | | | 0.16 (0.425) |  | 0.641 (0.365) |  | 0.684 (0.373) |  | 0.709 (0.358) |  |
| No prior HF hospitalisation (n =81) | | | 0.197 (0.409) | 0.169 | 0.672 (0.347) | 0.657 | 0.727 (0.354) | 0.195 | 0.759 (0.331) | 0.131 |
| Prior HF hospitalisation (n =119) | | | 0.118 (0.38) |  | 0.650 (0.341) |  | 0.654 (0.394) |  | 0.677 (0.383) |  |
| LVEF <10% (n =3) | | | 0.269 (0.607) | 0.915 | 0.850 (0.054) | 0.508 | 0.901 (0.101) | 0.642 | 0.901 (0.101) | 0.888 |
| LVEF 11-20% (n =52) | | | 0.175 (0.396) |  | 0.649 (0.332) |  | 0.691 (0.378) |  | 0.725 (0.357) |  |
| LVEF 21-30% (n =87) | | | 0.136 (0.374) |  | 0.676 (0.353) |  | 0.697 (0.359) |  | 0.723 (0.34) |  |
| LVEF 31-40% (n =58) | | | 0.142 (0.416) |  | 0.634 (0.347) |  | 0.646 (0.418) |  | 0.674 (0.409) |  |
| NYHA class 1 (n =72) | | | 0.289 (0.404) | **0.004** | 0.844 (0.19) | **<0.001** | 0.953 (0.096) | **<0.001** | 0.958 (0.085) | **<0.001** |
| NYHA class 2 (n =55) | | | 0.135 (0.366) |  | 0.711 (0.231) |  | 0.782 (0.185) |  | 0.792 (0.153) |  |
| NYHA class 3 (n =20) | | | 0.069 (0.361) |  | 0.513 (0.396) |  | 0.372 (0.375) |  | 0.363 (0.385) |  |
| NYHA class 4 (n =26) | | | 0.016 (0.366) |  | 0.353 (0.432) |  | 0.122 (0.303) |  | 0.128 (0.281) |  |
| CCI ≤3 (n =98) | | | 0.178 (0.376) | 0.516 | 0.674 (0.366) | 0.721 | 0.772 (0.337) | **0.049** | 0.81 (0.312) | **0.016** |
| CCI >3 (n =102) | | | 0.139 (0.4) |  | 0.654 (0.334) |  | 0.649 (0.39) |  | 0.673 (0.375) |  |
| No hypertension (n =70) | | | 0.183 (0.398) | 0.382 | 0.724 (0.297) | **0.039** | 0.709 (0.359) | 0.522 | 0.741 (0.335) | 0.432 |
| Hypertension (n =130) | | | 0.132 (0.391) |  | 0.625 (0.361) |  | 0.670 (0.39) |  | 0.697 (0.377) |  |
| No diabetes (n =72) | | | 0.175 (0.392) | 0.498 | 0.66 (0.332) | 0.976 | 0.676 (0.38) | 0.834 | 0.706 (0.358) | 0.878 |
| Diabetes (n =128) | | | 0.136 (0.394) |  | 0.659 (0.35) |  | 0.688 (0.38) |  | 0.715 (0.367) |  |
| No dyslipidemia (n =127) | | | 0.191 (0.404) | **0.045** | 0.654 (0.351) | 0.781 | 0.668 (0.389) | 0.454 | 0.706 (0.363) | 0.761 |
| Dyslipidemia (n =73) | | | 0.078 (0.364) |  | 0.668 (0.329) |  | 0.711 (0.363) |  | 0.723 (0.364) |  |
| Not obese (n =129) | | | 0.127 (0.386) | 0.281 | 0.643 (0.34) | 0.247 | 0.663 (0.38) | 0.369 | 0.689 (0.361) | 0.267 |
| Obese (n =71) | | | 0.191 (0.401) |  | 0.702 (0.335) |  | 0.718 (0.377) |  | 0.753 (0.363) |  |
| No IHD (n =82) | | | 0.193 (0.396) | 0.199 | 0.698 (0.333) | 0.179 | 0.696 (0.384) | 0.725 | 0.715 (0.371) | 0.930 |
| IHD (n =118) | | | 0.12 (0.39) |  | 0.632 (0.348) |  | 0.676 (0.377) |  | 0.710 (0.358) |  |
| No stroke (n =177) | | | 0.148 (0.379) | 0.888 | 0.675 (0.33) | 0.153 | 0.690 (0.376) | 0.545 | 0.720 (0.357) | 0.465 |
| Stroke (n =23) | | | 0.163 (0.495) |  | 0.541 (0.417) |  | 0.637 (0.41) |  | 0.647 (0.41) |  |
| No atrial fibrillation (n =151) | | | 0.134 (0.375) | 0.377 | 0.646 (0.346) | 0.314 | 0.682 (0.383) | 0.906 | 0.71 (0.365) | 0.902 |
| Atrial fibrillation (n =49) | | | 0.197 (0.443) |  | 0.701 (0.332) |  | 0.69 (0.37) |  | 0.718 (0.361) |  |
| No CABG (n =185) | | | 0.151 (0.39) | 0.864 | 0.675 (0.329) | 0.093 | 0.694 (0.371) | 0.192 | 0.724 (0.35) | 0.256 |
| CABG (n =15) | | | 0.131 (0.446) |  | 0.462 (0.451) |  | 0.556 (0.464) |  | 0.561 (0.484) |  |
| No PCI (n =159) | | | 0.142 (0.391) | 0.616 | 0.654 (0.345) | 0.684 | 0.679 (0.382) | 0.726 | 0.703 (0.368) | 0.516 |
| PCI (n =41) | | | 0.178 (0.403) |  | 0.679 (0.338) |  | 0.703 (0.375) |  | 0.746 (0.345) |  |
| No ICD (n =188) | | | 0.156 (0.393) | 0.384 | 0.66 (0.346) | 0.910 | 0.69 (0.376) | 0.383 | 0.72 (0.357) | 0.369 |
| ICD (n =12) | | | 0.052 (0.387) |  | 0.65 (0.298) |  | 0.587 (0.429) |  | 0.593 (0.439) |  |
| No valve (n =191) | | | 0.149 (0.391) | 0.945 | 0.664 (0.343) | 0.393 | 0.694 (0.371) | 0.071 | 0.726 (0.35) | 0.159 |
| Valve (n =9) | | | 0.16 (0.451) |  | 0.563 (0.327) |  | 0.46 (0.508) |  | 0.46 (0.508) |  |
| No CKD (n =140) | | | 0.176 (0.398) | 0.146 | 0.682 (0.343) | 0.144 | 0.725 (0.359) | **0.023** | 0.759 (0.336) | **0.016** |
| CKD (n =60) | | | 0.089 (0.376) |  | 0.605 (0.339) |  | 0.587 (0.41) |  | 0.603 (0.401) |  |
| No liver disease (n =177) | | | 0.16 (0.394) | 0.318 | 0.653 (0.35) | 0.421 | 0.695 (0.372) | 0.256 | 0.724 (0.355) | 0.300 |
| No liver disease (n =23) | | | 0.073 (0.381) |  | 0.705 (0.279) |  | 0.598 (0.429) |  | 0.624 (0.41) |  |
| No lung disease (n =183) | | | 0.15 (0.396) | 0.989 | 0.663 (0.347) | 0.610 | 0.693 (0.378) | 0.259 | 0.725 (0.358) | 0.170 |
| Lung disease (n =17) | | | 0.148 (0.37) |  | 0.623 (0.3) |  | 0.582 (0.387) |  | 0.579 (0.394) |  |
| No Anaemia (n =97) | | | 0.191 (0.378) | 0.144 | 0.705 (0.314) | 0.068 | 0.749 (0.34) | **0.026** | 0.784 (0.312) | **0.010** |
| Anaemia (n =103) | | | 0.11 (0.404) |  | 0.617 (0.364) |  | 0.622 (0.405) |  | 0.643 (0.394) |  |
| Not discharged with diuretics (n =45) | | | 0.22 (0.477) | 0.240 | 0.637 (0.384) | 0.648 | 0.665 (0.422) | 0.720 | 0.712 (0.406) | 0.995 |
| Discharged with diuretics (n =155) | | | 0.129 (0.364) |  | 0.666 (0.331) |  | 0.689 (0.367) |  | 0.712 (0.352) |  |
| Not discharged with ACEI (n =159) | | | 0.166 (0.401) | 0.214 | 0.664 (0.336) | 0.838 | 0.689 (0.383) | 0.714 | 0.72 (0.363) | 0.687 |
| Discharged with ACEI (n =41) | | | 0.086 (0.357) |  | 0.642 (0.371) |  | 0.664 (0.369) |  | 0.682 (0.365) |  |
| Not discharged with ARB (n =180) | | | 0.162 (0.4) | 0.111 | 0.67 (0.34) | 0.171 | 0.694 (0.37) | 0.271 | 0.729 (0.346) | 0.170 |
| Discharged with ARB (n =20) | | | 0.039 (0.307) |  | 0.561 (0.358) |  | 0.592 (0.455) |  | 0.556 (0.472) |  |
| Not discharged with ARNI (n =119) | | | 0.111 (0.387) | 0.093 | 0.627 (0.361) | 0.096 | 0.628 (0.402) | **0.016** | 0.65 (0.393) | **0.005** |
| Discharged with ARNI (n =81) | | | 0.207 (0.397) |  | 0.707 (0.309) |  | 0.766 (0.329) |  | 0.8 (0.294) |  |
| Not discharged with BB (n =22) | | | 0.067 (0.354) | 0.262 | 0.496 (0.456) | 0.079 | 0.414 (0.484) | **0.001** | 0.418 (0.498) | **0.011** |
| Discharged with BB (n =178) | | | 0.16 (0.397) |  | 0.679 (0.322) |  | 0.717 (0.352) |  | 0.748 (0.326) |  |
| Not discharged with MRA (n =43) | | | 0.167 (0.398) | 0.755 | 0.622 (0.347) | 0.434 | 0.646 (0.398) | 0.478 | 0.678 (0.379) | 0.531 |
| Discharged with MRA (n =157) | | | 0.145 (0.392) |  | 0.669 (0.342) |  | 0.694 (0.375) |  | 0.721 (0.359) |  |
| Not discharged with SGLT2i (n =54) | | | 0.165 (0.403) | 0.737 | 0.607 (0.386) | 0.232 | 0.631 (0.419) | 0.268 | 0.663 (0.411) | 0.339 |
| Discharged with SGLT2i (n =146) | | | 0.144 (0.39) |  | 0.678 (0.324) |  | 0.703 (0.363) |  | 0.729 (0.344) |  |
|  |  |  | | | | | | |  |  |

Independent t-test and ANOVA tests were used to examine the differences on the mean utility values of different subgroups. P-value <0.05 suggested significant difference between subgroups.

# Online Resource 9: Univariate analysis of GLMM for utility values

## (A) Univariate analysis using MI datasets

| Utility model (MI) | Estimate | SE | p-value | 95% LCI | 95% UCI |
| --- | --- | --- | --- | --- | --- |
| Age | -0.003 | 0.001 | **0.032** | -0.006 | -0.0003 |
| Female | -0.091 | 0.047 | 0.053 | -0.183 | 0.001 |
| Chinese | 0.043 | 0.047 | 0.364 | -0.049 | 0.135 |
| Indian | -0.123 | 0.058 | **0.034** | -0.236 | -0.010 |
| Others | 0.275 | 0.285 | 0.336 | -0.286 | 0.835 |
| EQ-5D-5L: English | -0.006 | 0.050 | 0.899 | -0.105 | 0.092 |
| EQ-5D-5L: Chinese | 0.067 | 0.051 | 0.188 | -0.033 | 0.166 |
| Married | -0.124 | 0.057 | **0.030** | -0.237 | -0.012 |
| Working | 0.201 | 0.044 | **0.000** | 0.114 | 0.289 |
| M40 | 0.050 | 0.124 | 0.687 | -0.193 | 0.293 |
| T20 | 0.144 | 0.110 | 0.194 | -0.073 | 0.360 |
| Education: Secondary | 0.011 | 0.048 | 0.812 | -0.083 | 0.105 |
| Education: Tertiary | 0.134 | 0.062 | **0.031** | 0.013 | 0.255 |
| Former smoker | -0.002 | 0.054 | 0.972 | -0.108 | 0.104 |
| Current smoker | 0.000 | 0.048 | 0.997 | -0.095 | 0.094 |
| BMI | 0.000 | 0.004 | 0.930 | -0.007 | 0.007 |
| Ischaemic HF | -0.048 | 0.044 | 0.272 | -0.133 | 0.038 |
| Recurrent HF | -0.067 | 0.046 | 0.145 | -0.158 | 0.023 |
| Year since diagnosis ≥ 1 year | -0.004 | 0.043 | 0.926 | -0.088 | 0.080 |
| Prior HF hospitalisation | -0.058 | 0.042 | 0.165 | -0.140 | 0.024 |
| Number of days in hospital | -0.011 | 0.005 | **0.040** | -0.021 | -0.001 |
| CCI | -0.029 | 0.012 | **0.018** | -0.053 | -0.005 |
| CCI ≥ 3 | -0.061 | 0.046 | 0.186 | -0.151 | 0.029 |
| Hypertension | -0.063 | 0.043 | 0.146 | -0.148 | 0.022 |
| Type 2 Diabetes | -0.009 | 0.043 | 0.828 | -0.094 | 0.075 |
| Dyslipidemia | -0.019 | 0.043 | 0.666 | -0.103 | 0.066 |
| Ischaemic heart disease | -0.053 | 0.042 | 0.207 | -0.135 | 0.029 |
| Stroke/TIA | -0.057 | 0.065 | 0.378 | -0.184 | 0.070 |
| Atrial fibrillation | 0.042 | 0.048 | 0.383 | -0.053 | 0.137 |
| Prior CABG | -0.124 | 0.078 | 0.112 | -0.277 | 0.029 |
| Prior PCI | 0.028 | 0.051 | 0.584 | -0.073 | 0.129 |
| Prior ICD | -0.072 | 0.087 | 0.405 | -0.243 | 0.098 |
| Valve replacement | -0.108 | 0.099 | 0.274 | -0.302 | 0.086 |
| CKD | -0.101 | 0.045 | **0.024** | -0.188 | -0.013 |
| Liver disease | -0.044 | 0.064 | 0.497 | -0.170 | 0.083 |
| Lung disease | -0.051 | 0.074 | 0.490 | -0.196 | 0.094 |
| Anaemia | -0.099 | 0.041 | **0.016** | -0.179 | -0.018 |
| LVEF | -0.002 | 0.003 | 0.488 | -0.007 | 0.003 |
| NYHA2 | -0.170 | 0.038 | **<0.001** | -0.246 | -0.095 |
| NYHA3 | -0.383 | 0.053 | **<0.001** | -0.488 | -0.278 |
| NYHA4 | -0.507 | 0.049 | **<0.001** | -0.604 | -0.411 |
| Serum haemoglobin | 0.026 | 0.009 | **0.005** | 0.008 | 0.045 |
| Serum HBA1C | 0.006 | 0.012 | 0.616 | -0.017 | 0.029 |
| Serum sodium | 0.007 | 0.005 | 0.126 | -0.002 | 0.016 |
| Serum potassium | -0.006 | 0.033 | 0.845 | -0.071 | 0.058 |
| Serum urea | -0.006 | 0.004 | 0.188 | -0.014 | 0.003 |
| Serum creatinine | -0.00002 | 0.000 | 0.937 | -0.0004 | 0.0004 |
| eGFR | 0.002 | 0.001 | **0.016** | 0.000 | 0.004 |
| SBP at discharge | 0.002 | 0.001 | 0.057 | 0.000 | 0.004 |
| Pre-admission diuretics | -0.038 | 0.042 | 0.370 | -0.120 | 0.045 |
| Pre-admission ACEI | 0.017 | 0.045 | 0.714 | -0.072 | 0.105 |
| Pre-admission ARB | -0.038 | 0.062 | 0.542 | -0.159 | 0.083 |
| Pre-admission ARNI | -0.027 | 0.049 | 0.573 | -0.123 | 0.068 |
| Pre-admission beta-blocker | -0.061 | 0.045 | 0.180 | -0.150 | 0.028 |
| Pre-admission MRA | -0.035 | 0.042 | 0.400 | -0.117 | 0.047 |
| Pre-admission SGLT2i | -0.023 | 0.042 | 0.583 | -0.105 | 0.059 |
| Pre-admission statin | -0.084 | 0.044 | 0.057 | -0.170 | 0.002 |
| Pre-admission antiplatelet | -0.071 | 0.042 | 0.091 | -0.155 | 0.012 |
| Pre-admission anti-coagulant | 0.037 | 0.057 | 0.512 | -0.074 | 0.149 |
| Pre-admission isosorbide | -0.048 | 0.120 | 0.692 | -0.284 | 0.188 |
| Pre-admission digoxin | -0.220 | 0.123 | 0.074 | -0.462 | 0.022 |
| Pre-admission trimetazidine | -0.025 | 0.071 | 0.722 | -0.166 | 0.115 |
| Pre-admission ivabradine | -0.179 | 0.077 | **0.020** | -0.330 | -0.028 |
| Pre-admission insulin | -0.104 | 0.051 | **0.041** | -0.204 | -0.004 |
| Pre-admission ezetimibe | 0.087 | 0.066 | 0.187 | -0.042 | 0.217 |
| Discharged diuretics | -0.013 | 0.050 | 0.801 | -0.110 | 0.085 |
| Discharged ACEI | -0.042 | 0.051 | 0.405 | -0.143 | 0.058 |
| Discharged ARB | -0.111 | 0.068 | 0.103 | -0.246 | 0.023 |
| Discharged ARNI | 0.104 | 0.042 | **0.012** | 0.023 | 0.186 |
| Discharged beta-blocker | 0.193 | 0.065 | **0.003** | 0.066 | 0.321 |
| Discharged MRA | 0.025 | 0.050 | 0.624 | -0.074 | 0.123 |
| Discharged SGLT-2i | 0.041 | 0.047 | 0.384 | -0.051 | 0.133 |
| Discharged statin | 0.006 | 0.048 | 0.904 | -0.089 | 0.101 |
| Discharged antiplatelet | -0.030 | 0.047 | 0.527 | -0.122 | 0.063 |
| Discharged anti-coagulant | 0.059 | 0.048 | 0.222 | -0.035 | 0.153 |
| Discharged isosorbide | 0.122 | 0.104 | 0.242 | -0.083 | 0.327 |
| Discharged digoxin | -0.084 | 0.091 | 0.360 | -0.263 | 0.096 |
| Discharged trimetazidine | -0.008 | 0.065 | 0.906 | -0.135 | 0.120 |
| Discharged ivabradine | -0.034 | 0.076 | 0.659 | -0.184 | 0.116 |
| Discharged insulin | -0.074 | 0.051 | 0.150 | -0.174 | 0.027 |
| Discharged ezetimibe | 0.041 | 0.059 | 0.485 | -0.074 | 0.157 |

MI: multiple imputation; SE: standard error; LCI: lower confidence interval; UCL: upper confidence interval

## (B) Univariate analysis using AV datasets

| Utility model (AV) | Estimate | SE | p-value | 95% LCI | 95% UCI |
| --- | --- | --- | --- | --- | --- |
| Age | -0.004 | 0.001 | **0.019** | -0.006 | -0.001 |
| Female | -0.099 | 0.047 | **0.036** | -0.192 | -0.006 |
| Chinese | 0.044 | 0.047 | 0.359 | -0.050 | 0.137 |
| Indian | -0.116 | 0.058 | **0.045** | -0.230 | -0.003 |
| Others | 0.272 | 0.288 | 0.345 | -0.294 | 0.839 |
| EQ-5D-5L: English | -0.016 | 0.050 | 0.755 | -0.115 | 0.083 |
| EQ-5D-5L: Chinese | 0.063 | 0.051 | 0.217 | -0.037 | 0.163 |
| Married | -0.116 | 0.058 | **0.047** | -0.229 | -0.002 |
| Working | 0.209 | 0.045 | **0.000** | 0.121 | 0.297 |
| M40 | 0.024 | 0.150 | 0.873 | -0.272 | 0.320 |
| T20 | 0.186 | 0.123 | 0.132 | -0.056 | 0.427 |
| Education: Secondary | 0.006 | 0.048 | 0.900 | -0.089 | 0.101 |
| Education: Tertiary | 0.117 | 0.062 | 0.063 | -0.006 | 0.240 |
| Former smoker | -0.003 | 0.053 | 0.962 | -0.107 | 0.102 |
| Current smoker | -0.004 | 0.048 | 0.936 | -0.099 | 0.091 |
| BMI | 0.002 | 0.003 | 0.563 | -0.005 | 0.009 |
| Ischaemic HF | -0.047 | 0.044 | 0.293 | -0.133 | 0.040 |
| Recurrent HF | -0.069 | 0.047 | 0.141 | -0.160 | 0.023 |
| Year since diagnosis ≥ 1 year | -0.009 | 0.042 | 0.831 | -0.093 | 0.074 |
| Prior HF hospitalisation | -0.063 | 0.042 | 0.138 | -0.146 | 0.020 |
| Number of days in hospital | -0.012 | 0.005 | **0.025** | -0.022 | -0.001 |
| CCI | -0.025 | 0.012 | **0.034** | -0.049 | -0.002 |
| CCI ≥ 3 | -0.058 | 0.045 | 0.201 | -0.148 | 0.031 |
| Hypertension | -0.062 | 0.043 | 0.152 | -0.146 | 0.023 |
| Type 2 Diabetes | -0.005 | 0.043 | 0.907 | -0.089 | 0.079 |
| Dyslipidemia | -0.031 | 0.043 | 0.465 | -0.115 | 0.053 |
| Ischaemic heart disease | -0.058 | 0.042 | 0.174 | -0.141 | 0.025 |
| Stroke/TIA | -0.060 | 0.063 | 0.345 | -0.185 | 0.065 |
| Atrial fibrillation | 0.046 | 0.048 | 0.345 | -0.049 | 0.141 |
| Prior CABG | -0.133 | 0.078 | 0.092 | -0.287 | 0.022 |
| Prior PCI | 0.030 | 0.052 | 0.558 | -0.071 | 0.132 |
| Prior ICD | -0.075 | 0.087 | 0.390 | -0.247 | 0.097 |
| Valve replacement | -0.101 | 0.094 | 0.281 | -0.285 | 0.083 |
| CKD | -0.100 | 0.044 | **0.025** | -0.187 | -0.013 |
| Liver disease | -0.043 | 0.065 | 0.507 | -0.171 | 0.085 |
| Lung disease | -0.059 | 0.074 | 0.423 | -0.205 | 0.087 |
| Anaemia | -0.099 | 0.040 | **0.015** | -0.179 | -0.019 |
| LVEF | -0.002 | 0.003 | 0.424 | -0.007 | 0.003 |
| NYHA2 | -0.149 | 0.037 | **<0.001** | -0.221 | -0.077 |
| NYHA3 | -0.380 | 0.052 | **<0.001** | -0.482 | -0.278 |
| NYHA4 | -0.524 | 0.046 | **<0.001** | -0.615 | -0.432 |
| Serum haemoglobin | 0.028 | 0.009 | **0.003** | 0.010 | 0.046 |
| Serum HBA1C | 0.006 | 0.012 | 0.601 | -0.018 | 0.030 |
| Serum sodium | 0.007 | 0.004 | 0.105 | -0.002 | 0.016 |
| Serum potassium | -0.007 | 0.033 | 0.838 | -0.071 | 0.058 |
| Serum urea | -0.006 | 0.004 | 0.165 | -0.014 | 0.002 |
| Serum creatinine | 0.000 | 0.000 | 0.959 | 0.000 | 0.000 |
| eGFR | 0.002 | 0.001 | **0.026** | 0.000 | 0.003 |
| SBP at discharge | 0.002 | 0.001 | **0.047** | 0.000 | 0.005 |
| Pre-admission diuretics | -0.045 | 0.042 | 0.283 | -0.128 | 0.038 |
| Pre-admission ACEI | 0.017 | 0.046 | 0.713 | -0.073 | 0.106 |
| Pre-admission ARB | -0.046 | 0.062 | 0.459 | -0.168 | 0.076 |
| Pre-admission ARNI | -0.036 | 0.049 | 0.460 | -0.133 | 0.060 |
| Pre-admission beta-blocker | -0.064 | 0.046 | 0.164 | -0.154 | 0.026 |
| Pre-admission MRA | -0.046 | 0.042 | 0.272 | -0.129 | 0.036 |
| Pre-admission SGLT2i | -0.024 | 0.042 | 0.562 | -0.107 | 0.058 |
| Pre-admission statin | -0.081 | 0.044 | 0.070 | -0.168 | 0.006 |
| Pre-admission antiplatelet | -0.068 | 0.042 | 0.110 | -0.151 | 0.015 |
| Pre-admission anti-coagulant | 0.041 | 0.057 | 0.466 | -0.070 | 0.153 |
| Pre-admission isosorbide | -0.052 | 0.121 | 0.667 | -0.290 | 0.186 |
| Pre-admission digoxin | -0.181 | 0.123 | 0.142 | -0.423 | 0.061 |
| Pre-admission trimetazidine | -0.034 | 0.072 | 0.635 | -0.176 | 0.107 |
| Pre-admission ivabradine | -0.168 | 0.078 | **0.032** | -0.322 | -0.015 |
| Pre-admission insulin | -0.097 | 0.050 | 0.055 | -0.195 | 0.002 |
| Pre-admission ezetimibe | 0.089 | 0.067 | 0.181 | -0.042 | 0.220 |
| Discharged diuretics | -0.014 | 0.049 | 0.770 | -0.111 | 0.082 |
| Discharged ACEI | -0.053 | 0.051 | 0.303 | -0.154 | 0.048 |
| Discharged ARB | -0.115 | 0.067 | 0.086 | -0.247 | 0.016 |
| Discharged ARNI | 0.102 | 0.042 | **0.015** | 0.020 | 0.185 |
| Discharged beta-blocker | 0.172 | 0.063 | **0.007** | 0.047 | 0.297 |
| Discharged MRA | 0.016 | 0.051 | 0.760 | -0.085 | 0.116 |
| Discharged SGLT-2i | 0.033 | 0.046 | 0.474 | -0.058 | 0.124 |
| Discharged statin | -0.002 | 0.049 | 0.961 | -0.098 | 0.093 |
| Discharged antiplatelet | -0.030 | 0.047 | 0.528 | -0.123 | 0.063 |
| Discharged anti-coagulant | 0.053 | 0.048 | 0.273 | -0.042 | 0.148 |
| Discharged isosorbide | 0.118 | 0.105 | 0.262 | -0.089 | 0.325 |
| Discharged digoxin | -0.092 | 0.092 | 0.316 | -0.273 | 0.088 |
| Discharged trimetazidine | -0.011 | 0.066 | 0.869 | -0.140 | 0.118 |
| Discharged ivabradine | -0.023 | 0.077 | 0.767 | -0.174 | 0.129 |
| Discharged insulin | -0.074 | 0.051 | 0.151 | -0.175 | 0.027 |
| Discharged ezetimibe | 0.054 | 0.059 | 0.359 | -0.062 | 0.171 |

AV: all available; SE: standard error; LCI: lower confidence interval; UCL: upper confidence interval

# Online Resource 10: Multivariable analysis using GLMM for utility values

## (A) Model 1: Base-case analysis using MI datasets (LASSO)

|  | Variables for utility values | Estimate | SE | p-value | 95% CI |
| --- | --- | --- | --- | --- | --- |
| 1 | (Intercept) | -0.622 | 0.468 | 0.184 | -1.541 - 0.297 |
| 2 | Age | 0.002 | 0.002 | 0.357 | -0.002 - 0.006 |
| 3 | Female | -0.087 | 0.039 | **0.027** | -0.163 - -0.01 |
| 4 | Language: English | -0.064 | 0.038 | 0.095 | -0.139 - 0.011 |
| 5 | Language: Chinese | 0.012 | 0.038 | 0.748 | -0.063 - 0.087 |
| 6 | Education: Secondary level | -0.009 | 0.035 | 0.799 | -0.078 - 0.06 |
| 7 | Education: Tertiary level | 0.024 | 0.049 | 0.632 | -0.073 - 0.12 |
| 8 | Working (Yes) | 0.077 | 0.040 | 0.055 | -0.002 - 0.155 |
| 9 | Former smoker | -0.048 | 0.042 | 0.246 | -0.13 - 0.033 |
| 10 | Current smoker | -0.088 | 0.039 | **0.024** | -0.165 - -0.012 |
| 11 | Hospital days | 0.014 | 0.020 | 0.471 | -0.024 - 0.052 |
| 12 | NYHA2 | -0.189 | 0.172 | 0.271 | -0.527 - 0.148 |
| 13 | NYHA3 | -0.234 | 0.303 | 0.439 | -0.829 - 0.361 |
| 14 | NYHA4 | -0.609 | 0.219 | **0.006** | -1.04 - -0.178 |
| 15 | Hypertension | -0.070 | 0.033 | **0.031** | -0.134 - -0.006 |
| 16 | Serum sodium | 0.005 | 0.003 | 0.185 | -0.002 - 0.011 |
| 17 | SBP at discharge | 0.003 | 0.001 | **0.004** | 0.001 - 0.004 |
| 18 | Pre-admission insulin | -0.056 | 0.048 | 0.245 | -0.151 - 0.039 |
| 19 | Pre-admission ezetimibe | 0.138 | 0.051 | **0.007** | 0.037 - 0.239 |
| 20 | Pre-admission anticoagulant | 0.054 | 0.041 | 0.184 | -0.026 - 0.135 |
| 21 | Discharged insulin | -0.032 | 0.048 | 0.512 | -0.127 - 0.063 |
| 22 | Time: Discharge | 0.510 | 0.028 | **<0.001** | 0.454 - 0.565 |
| 23 | Time: 1MPD | 0.534 | 0.030 | **<0.001** | 0.475 - 0.593 |
| 24 | Age*NYHA 2 | 0.001 | 0.003 | 0.821 | -0.005 - 0.006 |
| 25 | Age*NYHA 3 | -0.002 | 0.005 | 0.730 | -0.01 - 0.007 |
| 26 | Age*NYHA 4 | 0.002 | 0.003 | 0.519 | -0.004 - 0.009 |
| 27 | Age*Hospital days | 0.000 | 0.000 | 0.316 | -0.001 - 0 |

## (B) Model 2: Sensitivity analysis using AV datasets (LASSO)

|  | Variables for utility values | Estimate | SE | p-value | 95% CI |
| --- | --- | --- | --- | --- | --- |
| 1 | (Intercept) | -0.527 | 0.448 | 0.241 | -1.409 - 0.356 |
| 2 | Age | 0.002 | 0.002 | 0.439 | -0.002 - 0.006 |
| 3 | Female | -0.084 | 0.038 | **0.026** | -0.159 - -0.01 |
| 4 | Language: English | -0.081 | 0.038 | **0.032** | -0.155 - -0.007 |
| 5 | Language: Chinese | -0.011 | 0.037 | 0.772 | -0.083 - 0.061 |
| 6 | Education: Secondary level | -0.037 | 0.034 | 0.277 | -0.105 - 0.03 |
| 7 | Education: Tertiary level | 0.019 | 0.048 | 0.697 | -0.076 - 0.114 |
| 8 | Working | 0.056 | 0.038 | 0.144 | -0.019 - 0.13 |
| 9 | Former smoker | -0.034 | 0.040 | 0.395 | -0.112 - 0.044 |
| 10 | Current smoker | -0.084 | 0.037 | **0.026** | -0.158 - -0.01 |
| 11 | Hospital days | 0.015 | 0.018 | 0.426 | -0.022 - 0.051 |
| 12 | NYHA2 | -0.164 | 0.159 | 0.303 | -0.477 - 0.149 |
| 13 | NYHA3 | -0.142 | 0.285 | 0.618 | -0.704 - 0.419 |
| 14 | NYHA4 | -0.739 | 0.229 | **0.001** | -1.19 - -0.289 |
| 15 | Hypertension | -0.074 | 0.031 | **0.018** | -0.136 - -0.013 |
| 16 | Serum sodium | 0.004 | 0.003 | 0.201 | -0.002 - 0.011 |
| 17 | SBP at discharge | 0.003 | 0.001 | **0.002** | 0.001 - 0.004 |
| 18 | Pre-admission insulin | -0.046 | 0.046 | 0.322 | -0.136 - 0.045 |
| 19 | Pre-admission ezetimibe | 0.176 | 0.051 | **0.001** | 0.075 - 0.277 |
| 20 | Pre-admission anticoagulant | 0.051 | 0.039 | 0.196 | -0.026 - 0.129 |
| 21 | Discharged insulin | -0.041 | 0.047 | 0.381 | -0.133 - 0.051 |
| 22 | Time: Discharge | 0.514 | 0.030 | **<0.001** | 0.456 - 0.573 |
| 23 | Time: 1MPD | 0.536 | 0.030 | **<0.001** | 0.478 - 0.595 |
| 24 | Age*NYHA 2 | 0.000 | 0.003 | 0.861 | -0.005 - 0.005 |
| 25 | Age*NYHA 3 | -0.003 | 0.004 | 0.489 | -0.011 - 0.005 |
| 26 | Age*NYHA 4 | 0.004 | 0.004 | 0.302 | -0.003 - 0.011 |
| 27 | Age*Hospital days | 0.000 | 0.000 | 0.319 | -0.001 - 0 |

## (C) Model 3: Sensitivity analysis using MI datasets (literature and clinical relevance)

|  | Variables for utility values | Estimate | SE | p-value | 95% CI |
| --- | --- | --- | --- | --- | --- |
| 1 | (Intercept) | 0.074 | 0.201 | 0.712 | -0.32 - 0.469 |
| 2 | Age | 0.000 | 0.002 | 0.844 | -0.003 - 0.004 |
| 3 | Female | -0.034 | 0.039 | 0.379 | -0.11 - 0.042 |
| 4 | Ethnicity: Chinese | 0.070 | 0.097 | 0.471 | -0.12 - 0.259 |
| 5 | Ethnicity: Indian | 0.047 | 0.051 | 0.356 | -0.053 - 0.147 |
| 6 | EQ-5D-5L Language: English | -0.083 | 0.046 | 0.073 | -0.174 - 0.008 |
| 7 | EQ-5D-5L Language: Chinese | -0.027 | 0.101 | 0.789 | -0.226 - 0.172 |
| 8 | Working | 0.089 | 0.042 | **0.037** | 0.005 - 0.172 |
| 9 | Married | 0.005 | 0.050 | 0.914 | -0.092 - 0.103 |
| 10 | Education: Secondary level | -0.006 | 0.037 | 0.876 | -0.079 - 0.067 |
| 11 | Education: Tertiary level | 0.044 | 0.053 | 0.408 | -0.06 - 0.147 |
| 12 | CCI | 0.017 | 0.014 | 0.237 | -0.011 - 0.044 |
| 13 | Number of hospital stay | -0.005 | 0.004 | 0.206 | -0.013 - 0.003 |
| 14 | NYHA 2 | -0.171 | 0.180 | 0.344 | -0.524 - 0.183 |
| 15 | NYHA 3 | -0.355 | 0.324 | 0.273 | -0.993 - 0.282 |
| 16 | NYHA 4 | -0.493 | 0.234 | **0.035** | -0.953 - -0.034 |
| 17 | Serum haemoglobin level | 0.008 | 0.009 | 0.352 | -0.009 - 0.025 |
| 18 | eGFR | 0.000 | 0.001 | 0.575 | -0.001 - 0.002 |
| 19 | Pre-admission ivabradine | -0.064 | 0.062 | 0.308 | -0.186 - 0.059 |
| 20 | Pre-admission insulin | -0.083 | 0.043 | 0.053 | -0.167 - 0.001 |
| 21 | Discharged ARNI | 0.013 | 0.034 | 0.701 | -0.053 - 0.079 |
| 22 | Discharged Beta-blocker | 0.049 | 0.054 | 0.361 | -0.056 - 0.154 |
| 23 | Time: Discharge | 0.510 | 0.028 | **<0.001** | 0.454 - 0.565 |
| 24 | Time: 1MPD | 0.534 | 0.030 | **<0.001** | 0.475 - 0.593 |
| 25 | Age*NYHA 2 | 0.001 | 0.003 | 0.825 | -0.005 - 0.006 |
| 26 | Age*NYHA 3 | 0.000 | 0.005 | 0.952 | -0.009 - 0.01 |
| 27 | Age*NYHA 4 | 0.001 | 0.004 | 0.860 | -0.006 - 0.008 |

# Online Resource 11: Unadjusted mean VAS scores of HFrEF patients by subgroups

| Mean VAS scores (SD) | | | At admission (n=200) | p-value^†^ | At discharge (n=200) | p-value^†^ | At 1MPD (n=200) | p-value^†^ | At 1MPD (n=173) | p-value^†^ |
| --- | --- | --- | --- | --- | --- | --- | --- | --- | --- | --- |
| All patients | | | 38.2 (20.8) |  | 67 (19.2) |  | 68.9 (22) |  | 70.1 (20.6) |  |
| Age < 60 (n =121) | | | 39.8 (20.7) | 0.391 | 70.5 (18.6) | **0.035** | 74.1 (21.2) | **0.009** | 75.3 (19.7) | **0.006** |
| Age ≥ 60 (n =79) | | | 37.2 (20.9) |  | 64.7 (19.2) |  | 65.5 (22) |  | 66.6 (20.5) |  |
| Male (n =148) | | | 38.2 (21.5) | 0.996 | 66.9 (20) | 0.978 | 68.8 (22.2) | 0.896 | 70.6 (20.7) | 0.601 |
| Female (n =52) | | | 38.2 (19) |  | 67 (16.8) |  | 69.3 (21.7) |  | 68.7 (20.2) |  |
| Malay (n =110) | | | 38.6 (21.8) | 0.834 | 69.6 (18.8) | 0.063 | 72.6 (20.8) | 0.052 | 73.2 (19.7) | 0.086 |
| Chinese (n =56) | | | 39 (16.2) |  | 65.6 (16.8) |  | 65.8 (20.9) |  | 68 (19.4) |  |
| Indian (n =33) | | | 35.1 (24.6) |  | 60.9 (22.7) |  | 61.4 (25.7) |  | 61.5 (24.4) |  |
| Others (n=1) | | | 45 |  | 50 |  | 80 |  | 80 |  |
| Not married (n =30) | | | 36.9 (14.3) | 0.628 | 69.6 (17.8) | 0.395 | 74.5 (21.1) | 0.141 | 77.7 (17.6) | **0.030** |
| Married (n =170) | | | 38.4 (21.8) |  | 66.5 (19.4) |  | 67.9 (22.1) |  | 68.9 (20.8) |  |
| Not working (n =147) | | | 37.9 (21) | 0.761 | 64.8 (19.8) | **0.003** | 66.5 (22.4) | **0.012** | 67.3 (21.2) | **0.001** |
| Working (n =53) | | | 38.9 (20.4) |  | 73.1 (15.8) |  | 75.6 (19.6) |  | 77.8 (16.4) |  |
| Income level B40 (n =185) | | | 38.1 (20.8) | 0.847 | 66.7 (19.3) | 0.841 | 69 (22.3) | 0.825 | 69.9 (20.9) | 0.876 |
| Income level M40 (n =4) | | | 46.3 (28.7) |  | 67.5 (20.6) |  | 68.7 (18.3) |  | 76.7 (5.8) |  |
| Income level T20 (n =6) | | | 35.5 (18.6) |  | 71.7 (14.7) |  | 68.2 (18.3) |  | 72 (11) |  |
| Primary school and below (n =55) | | | 40 (20) | 0.750 | 70.1 (17.3) | 0.321 | 65.5 (23.9) | **0.025** | 67.2 (21.4) | 0.088 |
| Secondary school (n =109) | | | 37.5 (20.5) |  | 64.6 (20.7) |  | 67.7 (21.8) |  | 69.1 (20.7) |  |
| High school and above (n =36) | | | 37.6 (23.2) |  | 69.2 (16.3) |  | 77.8 (17.7) |  | 77.1 (17.9) |  |
| Non-smoker (n =99) | | | 37.8 (20.5) | 0.953 | 67.4 (18.7) | 0.981 | 67.9 (22.9) | 0.813 | 69.2 (21.1) | 0.845 |
| Active (n =42) | | | 38.1 (22.5) |  | 66.4 (17.6) |  | 69.7 (23.2) |  | 69.6 (23) |  |
| Ex-smoker (n =59) | | | 39 (20.4) |  | 66.6 (21.3) |  | 70 (19.8) |  | 72 (18.1) |  |
| Underweight (n =9) | | | 38.3 (19) | 0.151 | 61.1 (14.7) | 0.544 | 64 (19.5) | 0.206 | 60 (19.1) | **0.043** |
| Normal BMI (n =47) | | | 41.5 (20.3) |  | 66.2 (17.9) |  | 64.2 (23.4) |  | 63.5 (22.3) |  |
| Overweight (n =62) | | | 33.7 (19.8) |  | 66 (21.5) |  | 69 (23.9) |  | 72 (22) |  |
| Obese (n =71) | | | 40.4 (22.2) |  | 69 (17.9) |  | 72.6 (19.1) |  | 73.7 (16.8) |  |
| Non-ischaemic (n =67) | | | 37.8 (19.1) | 0.832 | 71.5 (14.4) | **0.007** | 72.9 (21.7) | 0.075 | 72.9 (21.1) | 0.213 |
| Ischaemic (n =133) | | | 38.4 (21.7) |  | 64.7 (20.9) |  | 66.9 (22) |  | 68.7 (20.2) |  |
| De novo HF (n =54) | | | 35.7 (20.8) | 0.307 | 66.1 (18.3) | 0.696 | 71.8 (20) | 0.264 | 73.2 (18.3) | 0.178 |
| Recurrent HF (n =146) | | | 39.1 (20.8) |  | 67.3 (19.5) |  | 67.8 (22.7) |  | 68.9 (21.3) |  |
| Year since diagnosis <1 year (n =125) | | | 38.1 (20.5) | 0.944 | 67.1 (17.6) | 0.925 | 69.2 (22.2) | 0.826 | 71 (20.6) | 0.488 |
| Year since diagnosis ≥ 1 year (n =75) | | | 38.3 (21.5) |  | 66.8 (21.7) |  | 68.4 (21.8) |  | 68.7 (20.5) |  |
| No prior HF hospitalisation (n =81) | | | 38.7 (21.9) | 0.760 | 67.6 (18.3) | 0.686 | 70.1 (20.4) | 0.557 | 71.6 (18.7) | 0.414 |
| Prior HF hospitalisation (n =119) | | | 37.8 (20.1) |  | 66.5 (19.8) |  | 68.1 (23.1) |  | 69.1 (21.9) |  |
| LVEF <10% (n =3) | | | 46.7 (32.2) | 0.398 | 60 (26.5) | 0.099 | 88.3 (2.9) | 0.202 | 88.3 (2.9) | 0.251 |
| LVEF 11-20% (n =52) | | | 40 (16.6) |  | 61.3 (21.2) |  | 64.6 (22) |  | 68 (19.8) |  |
| LVEF 21-30% (n =87) | | | 38.9 (21.5) |  | 70.3 (17.1) |  | 70.5 (21.5) |  | 71 (20.2) |  |
| LVEF 31-40% (n =58) | | | 35.1 (22.6) |  | 67.4 (19.1) |  | 69.4 (22.9) |  | 69.6 (22.1) |  |
| NYHA class 1 (n =72) | | | 40.8 (20.7) | 0.424 | 74.8 (14.5) | **<0.001** | 84.6 (12.2) | **<0.001** | 84.4 (12.1) | **<0.001** |
| NYHA class 2 (n =55) | | | 35.1 (20.4) |  | 64.2 (15.5) |  | 69.2 (16.5) |  | 68.9 (14.2) |  |
| NYHA class 3 (n =20) | | | 41.5 (18.5) |  | 69.5 (18.4) |  | 46.9 (19.2) |  | 48.5 (18.7) |  |
| NYHA class 4 (n =26) | | | 39.1 (24.7) |  | 53.8 (24.1) |  | 49.4 (21.3) |  | 50 (20.1) |  |
| CCI ≤3 (n =98) | | | 39.3 (19) | 0.607 | 67.6 (18.9) | 0.771 | 72.1 (21.5) | 0.216 | 75.2 (17.8) | **0.028** |
| CCI >3 (n =102) | | | 37.7 (21.5) |  | 66.7 (19.3) |  | 67.7 (22.2) |  | 68.1 (21.3) |  |
| No hypertension (n =70) | | | 41.2 (21.4) | 0.141 | 69.4 (20.4) | 0.205 | 66.3 (24.6) | 0.243 | 67.9 (23.2) | 0.332 |
| Hypertension (n =130) | | | 36.6 (20.4) |  | 65.7 (18.4) |  | 70.3 (20.5) |  | 71.3 (19) |  |
| No diabetes (n =72) | | | 39.9 (19.2) | 0.378 | 68.1 (17.6) | 0.505 | 67.7 (22.5) | 0.574 | 69.5 (20.4) | 0.768 |
| Diabetes (n =128) | | | 37.2 (21.7) |  | 66.3 (20) |  | 69.6 (21.8) |  | 70.5 (20.7) |  |
| No dyslipidemia (n =127) | | | 39.7 (21.2) | 0.168 | 66.9 (19.5) | 0.976 | 67.6 (22.5) | 0.277 | 69.9 (20.8) | 0.838 |
| Dyslipidemia (n =73) | | | 35.6 (19.9) |  | 67 (18.7) |  | 71.2 (21.1) |  | 70.6 (20.3) |  |
| Not obese (n =129) | | | 37.1 (20.1) | 0.316 | 65.7 (19.6) | 0.243 | 66.7 (23.4) | 0.082 | 67.9 (22.2) | 0.058 |
| Obese (n =71) | | | 40.4 (22.2) |  | 69 (17.9) |  | 72.6 (19.1) |  | 73.7 (16.8) |  |
| No IHD (n =82) | | | 39.8 (20.5) | 0.355 | 70.7 (16) | **0.015** | 72.7 (21.3) | 0.052 | 73.7 (20.3) | 0.051 |
| IHD (n =118) | | | 37.1 (21) |  | 64.3 (20.7) |  | 66.3 (22.2) |  | 67.5 (20.4) |  |
| No stroke (n =177) | | | 38.6 (20.1) | 0.554 | 67 (18.9) | 0.917 | 69.4 (22) | 0.377 | 70.6 (20.5) | 0.439 |
| Stroke (n =23) | | | 35.2 (25.8) |  | 66.5 (21.7) |  | 64.9 (22.3) |  | 66.6 (20.9) |  |
| No atrial fibrillation (n =151) | | | 37.5 (20.2) | 0.463 | 66.6 (19.5) | 0.634 | 70 (21) | 0.257 | 70.8 (19.7) | 0.499 |
| Atrial fibrillation (n =49) | | | 40.2 (22.6) |  | 68.1 (18.2) |  | 65.6 (24.8) |  | 68.1 (23.3) |  |
| No CABG (n =185) | | | 38 (20.9) | 0.573 | 67.7 (19.1) | **0.043** | 69.2 (22.5) | 0.494 | 70.7 (20.9) | 0.107 |
| CABG (n =15) | | | 41.1 (20.6) |  | 57.5 (17.4) |  | 65 (15.4) |  | 63.5 (14.1) |  |
| No PCI (n =159) | | | 38.8 (20.7) | 0.441 | 67.9 (18.5) | 0.196 | 70 (21.8) | 0.174 | 70.8 (20.7) | 0.395 |
| PCI (n =41) | | | 35.9 (21.2) |  | 63.2 (21.3) |  | 64.6 (22.5) |  | 67.5 (20.1) |  |
| No ICD (n =188) | | | 38.2 (21) | 0.953 | 67.4 (19) | 0.273 | 69.5 (21.7) | 0.136 | 70.9 (20) | 0.165 |
| ICD (n =12) | | | 38.5 (18.5) |  | 60.2 (21.3) |  | 59.4 (25.1) |  | 59.1 (25.8) |  |
| No valve (n =191) | | | 38.2 (21) | 0.940 | 66.9 (19.4) | 0.770 | 69.5 (22) | 0.105 | 70.8 (20.3) | 0.096 |
| Valve (n =9) | | | 37.8 (16) |  | 68.3 (13.7) |  | 57.2 (21.4) |  | 57.2 (21.4) |  |
| No CKD (n =140) | | | 38.6 (21.1) | 0.656 | 68.1 (20.2) | 0.183 | 70.4 (22.2) | 0.157 | 72.1 (20.3) | 0.058 |
| CKD (n =60) | | | 37.2 (20.1) |  | 64.4 (16.4) |  | 65.4 (21.5) |  | 65.6 (20.5) |  |
| No liver disease (n =177) | | | 38.2 (21) | 0.941 | 67 (19.5) | 0.947 | 69.5 (21.9) | 0.273 | 71 (20.3) | 0.183 |
| No liver disease (n =23) | | | 38.5 (20) |  | 66.7 (16.6) |  | 64 (22.6) |  | 64.1 (21.9) |  |
| No lung disease (n =183) | | | 38.7 (20.7) | 0.348 | 67.5 (19.2) | 0.202 | 69.3 (22.2) | 0.439 | 70.5 (20.6) | 0.409 |
| Lung disease (n =17) | | | 33.2 (22.3) |  | 61.2 (18.8) |  | 64.9 (20.3) |  | 66.1 (19.8) |  |
| No Anaemia (n =97) | | | 41.2 (20.7) | **0.044** | 70.1 (18) | **0.022** | 72.7 (21.5) | **0.023** | 74.5 (19.5) | **0.006** |
| Anaemia (n =103) | | | 35.3 (20.6) |  | 64 (19.8) |  | 65.4 (22) |  | 66 (20.7) |  |
| Not discharged with diuretics (n =45) | | | 38.7 (22.7) | 0.777 | 67.7 (20.5) | 0.670 | 70.3 (23.9) | 0.466 | 73.7 (21.1) | 0.055 |
| Discharged with diuretics (n =155) | | | 37.8 (19.4) |  | 66.5 (18.2) |  | 67.9 (20.6) |  | 67.6 (19.8) |  |
| Not discharged with ACEI (n =159) | | | 38.5 (21.5) | 0.740 | 67.9 (19.2) | 0.279 | 70 (23) | 0.289 | 71.6 (21.6) | 0.137 |
| Discharged with ACEI (n =41) | | | 37.5 (19.3) |  | 64.7 (19) |  | 66.3 (19.5) |  | 66.9 (17.9) |  |
| Not discharged with ARB (n =180) | | | 38.8 (21.1) | 0.248 | 67 (19) | 0.928 | 68.6 (22) | 0.639 | 70.1 (20.4) | 0.959 |
| Discharged with ARB (n =20) | | | 34.1 (18.6) |  | 66.6 (20.9) |  | 70.9 (22.8) |  | 70.4 (22.4) |  |
| Not discharged with ARNI (n =119) | | | 38.7 (20.4) | 0.531 | 66.8 (19.3) | 0.827 | 69 (21.8) | 0.920 | 70.2 (20.3) | 0.918 |
| Discharged with ARNI (n =81) | | | 36.4 (22.3) |  | 67.5 (18.9) |  | 68.6 (23) |  | 69.8 (21.6) |  |
| Not discharged with BB (n =22) | | | 35.9 (22.7) | 0.362 | 69.1 (20.1) | 0.330 | 73.6 (21) | 0.067 | 74.8 (19.3) | **0.049** |
| Discharged with BB (n =178) | | | 39.1 (20) |  | 66.1 (18.8) |  | 67 (22.2) |  | 68.2 (20.8) |  |
| Not discharged with MRA (n =43) | | | 38.6 (21.5) | 0.755 | 67.4 (19.8) | 0.734 | 69.9 (22.5) | 0.489 | 72 (20.7) | 0.181 |
| Discharged with MRA (n =157) | | | 37.7 (20) |  | 66.4 (18.5) |  | 67.7 (21.4) |  | 67.8 (20.2) |  |
| Not discharged with SGLT2i (n =54) | | | 37.1 (20.8) | 0.389 | 66.6 (20.2) | 0.755 | 70.3 (22.4) | 0.332 | 71.8 (20.5) | 0.208 |
| Discharged with SGLT2i (n =146) | | | 39.6 (20.8) |  | 67.4 (17.8) |  | 67.1 (21.6) |  | 67.9 (20.5) |  |
|  |  |  | | | | | | |  |  |

Independent t-test and ANOVA tests were used to examine the differences on the mean VAS scores of different subgroups. P-value <0.05 suggested significant difference between subgroups.

# Online Resource 12: Univariate analysis of GLMM for VAS scores

## (A) Univariate analysis using MI datasets

| VAS model (MI) | Estimate | SE | p-value | 95% LCI | 95% UCI |
| --- | --- | --- | --- | --- | --- |
| Age | -0.20 | 0.08 | **0.008** | -0.35 | -0.05 |
| Female | 0.18 | 2.38 | 0.939486 | -4.50 | 4.86 |
| Chinese | -3.47 | 2.38 | 0.145 | -8.14 | 1.20 |
| Indian | -7.86 | 2.91 | **0.007** | -13.56 | -2.15 |
| Others | -1.96 | 14.44 | 0.892 | -30.33 | 26.41 |
| EQ-5D-5L: English | -2.89 | 2.53 | 0.255 | -7.87 | 2.09 |
| EQ-5D-5L: Chinese | -2.17 | 2.56 | 0.398 | -7.20 | 2.87 |
| Married | -2.73 | 2.92 | 0.350 | -8.47 | 3.01 |
| Working | 6.13 | 2.33 | **0.009** | 1.56 | 10.70 |
| M40 | 2.85 | 6.39 | 0.656 | -9.71 | 15.41 |
| T20 | 0.59 | 5.63 | 0.916 | -10.48 | 11.66 |
| Education: Secondary | -1.93 | 2.43 | 0.427 | -6.70 | 2.84 |
| Education: Tertiary | 3.02 | 3.14 | 0.337 | -3.15 | 9.19 |
| Former smoker | 0.41 | 2.72 | 0.880 | -4.93 | 5.75 |
| Current smoker | 0.87 | 2.43 | 0.719 | -3.90 | 5.65 |
| BMI | 0.11 | 0.18 | 0.546 | -0.24 | 0.45 |
| Ischaemic HF | -4.07 | 2.19 | 0.064 | -8.38 | 0.24 |
| Recurrent HF | 0.18 | 2.35 | 0.939 | -4.44 | 4.80 |
| Year since diagnosis ≥ 1 year | -0.27 | 2.16 | 0.902 | -4.52 | 3.98 |
| Prior HF hospitalisation | -1.33 | 2.13 | 0.534 | -5.52 | 2.86 |
| Number of days in hospital | -0.14 | 0.26 | 0.578 | -0.65 | 0.37 |
| CCI | -1.57 | 0.62 | **0.012** | -2.78 | -0.35 |
| CCI ≥ 3 | -2.31 | 2.32 | 0.321 | -6.87 | 2.26 |
| Hypertension | -1.45 | 2.20 | 0.508 | -5.77 | 2.86 |
| Type 2 Diabetes | -0.85 | 2.18 | 0.695 | -5.12 | 3.42 |
| Dyslipidemia | -0.14 | 2.17 | 0.949 | -4.41 | 4.13 |
| Ischaemic heart disease | -5.18 | 2.10 | **0.014** | -9.31 | -1.06 |
| Stroke/TIA | -2.79 | 3.28 | 0.396 | -9.24 | 3.66 |
| Atrial fibrillation | -0.07 | 2.45 | 0.977 | -4.88 | 4.73 |
| Prior CABG | -3.77 | 3.97 | 0.343 | -11.56 | 4.03 |
| Prior PCI | -4.36 | 2.57 | 0.091 | -9.41 | 0.70 |
| Prior ICD | -5.67 | 4.39 | 0.197 | -14.29 | 2.94 |
| Valve replacement | -3.74 | 5.00 | 0.455 | -13.56 | 6.08 |
| CKD | -3.35 | 2.27 | 0.141 | -7.82 | 1.12 |
| Liver disease | -1.80 | 3.27 | 0.581 | -8.22 | 4.62 |
| Lung disease | -5.38 | 3.72 | 0.148 | -12.68 | 1.92 |
| Anaemia | -6.48 | 2.04 | **0.002** | -10.49 | -2.46 |
| LVEF | 0.10 | 0.14 | 0.482 | -0.17 | 0.37 |
| NYHA2 | -11.09 | 2.18 | **<0.001** | -15.37 | -6.81 |
| NYHA3 | -15.41 | 3.16 | **<0.001** | -21.62 | -9.21 |
| NYHA4 | -18.74 | 2.78 | **<0.001** | -24.20 | -13.29 |
| Serum haemoglobin | 1.15 | 0.47 | **0.016** | 0.22 | 2.08 |
| Serum HBA1C | 0.22 | 0.59 | 0.716 | -0.95 | 1.38 |
| Serum sodium | 0.25 | 0.24 | 0.292 | -0.21 | 0.71 |
| Serum potassium | 0.46 | 1.68 | 0.785 | -2.85 | 3.76 |
| Serum urea | -0.13 | 0.21 | 0.536 | -0.55 | 0.29 |
| Serum creatinine | 0.00 | 0.01 | 0.868 | -0.02 | 0.02 |
| eGFR | 0.08 | 0.04 | 0.053 | 0.00 | 0.16 |
| SBP at discharge | 0.10 | 0.06 | 0.091 | -0.02 | 0.21 |
| Pre-admission diuretics | -1.49 | 2.12 | 0.482 | -5.66 | 2.68 |
| Pre-admission ACEI | -2.66 | 2.27 | 0.243 | -7.12 | 1.81 |
| Pre-admission ARB | -0.92 | 3.13 | 0.770 | -7.07 | 5.24 |
| Pre-admission ARNI | -0.66 | 2.47 | 0.788 | -5.52 | 4.19 |
| Pre-admission beta-blocker | -2.14 | 2.32 | 0.355 | -6.69 | 2.40 |
| Pre-admission MRA | -1.36 | 2.10 | 0.518 | -5.49 | 2.77 |
| Pre-admission SGLT2i | 0.08 | 2.11 | 0.970 | -4.07 | 4.23 |
| Pre-admission statin | -2.63 | 2.23 | 0.239 | -7.02 | 1.76 |
| Pre-admission antiplatelet | -4.64 | 2.13 | **0.030** | -8.82 | -0.46 |
| Pre-admission anti-coagulant | 0.29 | 2.88 | 0.919 | -5.36 | 5.94 |
| Pre-admission isosorbide | -9.29 | 6.05 | 0.125 | -21.17 | 2.58 |
| Pre-admission digoxin | -8.62 | 6.22 | 0.167 | -20.84 | 3.60 |
| Pre-admission trimetazidine | -5.33 | 3.69 | 0.150 | -12.59 | 1.93 |
| Pre-admission ivabradine | -6.30 | 3.94 | 0.111 | -14.04 | 1.44 |
| Pre-admission insulin | -3.93 | 2.58 | 0.128 | -8.99 | 1.14 |
| Pre-admission ezetimibe | 1.30 | 3.37 | 0.699 | -5.31 | 7.91 |
| Discharged diuretics | 2.49 | 2.51 | 0.322 | -2.44 | 7.42 |
| Discharged ACEI | -3.46 | 2.57 | 0.179 | -8.52 | 1.59 |
| Discharged ARB | -4.51 | 3.47 | 0.194 | -11.32 | 2.30 |
| Discharged ARNI | 5.07 | 2.10 | **0.016** | 0.94 | 9.19 |
| Discharged beta-blocker | 10.11 | 3.29 | **0.002** | 3.66 | 16.57 |
| Discharged MRA | -0.85 | 2.55 | 0.739 | -5.86 | 4.16 |
| Discharged SGLT-2i | -1.22 | 2.37 | 0.607 | -5.88 | 3.43 |
| Discharged statin | 3.12 | 2.44 | 0.202 | -1.68 | 7.91 |
| Discharged antiplatelet | -3.44 | 2.37 | 0.147 | -8.10 | 1.21 |
| Discharged anti-coagulant | 0.17 | 2.43 | 0.945 | -4.60 | 4.93 |
| Discharged isosorbide | -0.75 | 5.30 | 0.887 | -11.15 | 9.65 |
| Discharged digoxin | -3.27 | 4.61 | 0.478 | -12.32 | 5.77 |
| Discharged trimetazidine | -3.11 | 3.33 | 0.350 | -9.65 | 3.42 |
| Discharged ivabradine | -6.06 | 3.83 | 0.115 | -13.58 | 1.47 |
| Discharged insulin | 0.99 | 2.60 | 0.704 | -4.11 | 6.08 |
| Discharged ezetimibe | 0.03 | 2.99 | 0.991 | -5.85 | 5.91 |

## (B) Univariate analysis using AV datasets

| VAS model (AV) | Estimate | SE | p-value | 95% LCI | 95% UCI |
| --- | --- | --- | --- | --- | --- |
| Age | -0.2 | 0.1 | **0.006** | -0.4 | -0.1 |
| Female | -0.7 | 2.4 | 0.776 | -5.4 | 4.0 |
| Chinese | -2.9 | 2.4 | 0.229 | -7.6 | 1.8 |
| Indian | -7.4 | 2.9 | **0.012** | -13.1 | -1.7 |
| Others | -2.0 | 14.4 | 0.889 | -30.4 | 26.4 |
| EQ-5D-5L: English | -3.5 | 2.5 | 0.170 | -8.5 | 1.5 |
| EQ-5D-5L: Chinese | -1.8 | 2.6 | 0.478 | -6.9 | 3.2 |
| Married | -3.2 | 2.9 | 0.273 | -9.0 | 2.6 |
| Working | 6.4 | 2.3 | **0.006** | 1.8 | 11.0 |
| M40 | 4.5 | 7.6 | 0.557 | -10.6 | 19.5 |
| T20 | 1.9 | 6.2 | 0.764 | -10.4 | 14.1 |
| Education: Secondary | -2.1 | 2.4 | 0.394 | -6.9 | 2.7 |
| Education: Tertiary | 2.0 | 3.2 | 0.535 | -4.3 | 8.2 |
| Former smoker | 0.3 | 2.7 | 0.905 | -5.0 | 5.6 |
| Current smoker | 1.1 | 2.4 | 0.660 | -3.7 | 5.9 |
| BMI | 0.2 | 0.2 | 0.343 | -0.2 | 0.5 |
| Ischaemic HF | -3.4 | 2.2 | 0.122 | -7.8 | 0.9 |
| Recurrent HF | 0.3 | 2.4 | 0.886 | -4.3 | 5.0 |
| Year since diagnosis ≥ 1 year | -0.4 | 2.1 | 0.835 | -4.7 | 3.8 |
| Prior HF hospitalisation | -1.6 | 2.1 | 0.455 | -5.8 | 2.6 |
| Number of days in hospital | -0.2 | 0.3 | 0.343 | -0.8 | 0.3 |
| CCI | -1.7 | 0.6 | **0.005** | -2.9 | -0.5 |
| CCI ≥ 3 | -3.3 | 2.3 | 0.159 | -7.8 | 1.3 |
| Hypertension | -2.0 | 2.2 | 0.361 | -6.3 | 2.3 |
| Type 2 Diabetes | -1.3 | 2.2 | 0.551 | -5.6 | 3.0 |
| Dyslipidemia | -1.2 | 2.2 | 0.568 | -5.5 | 3.0 |
| Ischaemic heart disease | -5.2 | 2.1 | **0.015** | -9.3 | -1.0 |
| Stroke/TIA | -3.1 | 3.2 | 0.344 | -9.5 | 3.3 |
| Atrial fibrillation | 0.7 | 2.4 | 0.783 | -4.1 | 5.5 |
| Prior CABG | -4.2 | 4.0 | 0.294 | -12.0 | 3.6 |
| Prior PCI | -3.8 | 2.6 | 0.140 | -8.9 | 1.3 |
| Prior ICD | -5.8 | 4.4 | 0.189 | -14.4 | 2.8 |
| Valve replacement | -2.8 | 4.8 | 0.571 | -12.3 | 6.9 |
| CKD | -4.0 | 2.3 | 0.075 | -8.5 | 0.4 |
| Liver disease | -2.1 | 3.3 | 0.518 | -8.5 | 4.3 |
| Lung disease | -5.6 | 3.7 | 0.135 | -12.9 | 1.7 |
| Anaemia | -7.0 | 2.0 | **0.001** | -11.0 | -3.0 |
| LVEF | 0.0 | 0.1 | 0.890 | -0.3 | 0.3 |
| NYHA2 | -10.5 | 2.2 | **<0.001** | -14.8 | -6.1 |
| NYHA3 | -13.3 | 3.1 | **<0.001** | -19.5 | -7.1 |
| NYHA4 | -18.5 | 2.8 | **<0.001** | -24.0 | -12.9 |
| Serum haemoglobin | 1.4 | 0.5 | **0.004** | 0.4 | 2.3 |
| Serum HBA1C | 0.1 | 0.6 | 0.818 | -1.1 | 1.3 |
| Serum sodium | 0.3 | 0.2 | 0.174 | -0.1 | 0.8 |
| Serum potassium | 0.6 | 1.7 | 0.701 | -2.6 | 3.9 |
| Serum urea | -0.1 | 0.2 | 0.499 | -0.6 | 0.3 |
| Serum creatinine | 0.0 | 0.0 | 0.966 | 0.0 | 0.0 |
| eGFR | 0.1 | 0.0 | **0.038** | 0.0 | 0.2 |
| SBP at discharge | 0.1 | 0.1 | 0.101 | 0.0 | 0.2 |
| Pre-admission diuretics | -2.2 | 2.1 | 0.294 | -6.4 | 1.9 |
| Pre-admission ACEI | -2.8 | 2.3 | 0.222 | -7.3 | 1.7 |
| Pre-admission ARB | -1.7 | 3.1 | 0.589 | -7.8 | 4.4 |
| Pre-admission ARNI | -0.9 | 2.5 | 0.719 | -5.8 | 4.0 |
| Pre-admission beta-blocker | -1.9 | 2.3 | 0.417 | -6.4 | 2.7 |
| Pre-admission MRA | -1.8 | 2.1 | 0.385 | -6.0 | 2.3 |
| Pre-admission SGLT2i | 0.0 | 2.1 | 0.986 | -4.1 | 4.2 |
| Pre-admission statin | -3.1 | 2.2 | 0.163 | -7.5 | 1.3 |
| Pre-admission antiplatelet | -4.3 | 2.1 | **0.043** | -8.5 | -0.1 |
| Pre-admission anti-coagulant | 0.5 | 2.9 | 0.859 | -5.1 | 6.1 |
| Pre-admission isosorbide | -9.6 | 6.0 | 0.111 | -21.5 | 2.2 |
| Pre-admission digoxin | -5.6 | 6.2 | 0.370 | -17.9 | 6.7 |
| Pre-admission trimetazidine | -5.2 | 3.6 | 0.152 | -12.3 | 1.9 |
| Pre-admission ivabradine | -5.4 | 4.0 | 0.170 | -13.2 | 2.3 |
| Pre-admission insulin | -5.0 | 2.5 | 0.053 | -10.0 | 0.0 |
| Pre-admission ezetimibe | 0.9 | 3.4 | 0.788 | -5.7 | 7.5 |
| Discharged diuretics | 2.3 | 2.5 | 0.360 | -2.6 | 7.2 |
| Discharged ACEI | -4.0 | 2.6 | 0.119 | -9.1 | 1.0 |
| Discharged ARB | -4.3 | 3.4 | 0.207 | -11.0 | 2.5 |
| Discharged ARNI | 5.1 | 2.1 | **0.016** | 1.0 | 9.2 |
| Discharged beta-blocker | 10.3 | 3.2 | **0.002** | 4.0 | 16.7 |
| Discharged MRA | -1.0 | 2.6 | 0.689 | -6.1 | 4.0 |
| Discharged SGLT-2i | -0.8 | 2.3 | 0.735 | -5.4 | 3.8 |
| Discharged statin | 2.7 | 2.4 | 0.274 | -2.1 | 7.5 |
| Discharged antiplatelet | -2.9 | 2.4 | 0.224 | -7.6 | 1.8 |
| Discharged anti-coagulant | -0.4 | 2.4 | 0.861 | -5.2 | 4.4 |
| Discharged isosorbide | -1.1 | 5.3 | 0.838 | -11.5 | 9.3 |
| Discharged digoxin | -4.4 | 4.6 | 0.344 | -13.5 | 4.7 |
| Discharged trimetazidine | -3.5 | 3.3 | 0.288 | -10.0 | 3.0 |
| Discharged ivabradine | -4.5 | 3.9 | 0.247 | -12.1 | 3.1 |
| Discharged insulin | 0.5 | 2.6 | 0.857 | -4.6 | 5.6 |
| Discharged ezetimibe | 0.8 | 3.0 | 0.794 | -5.1 | 6.7 |

# Online Resource 13: Multivariable analysis using GLMM for VAS scores

## (A) Model 1: Base-case analysis using MI datasets (LASSO)

|  | Variables for VAS model | Estimate | SE | p-value | 95% CI |
| --- | --- | --- | --- | --- | --- |
| 1 | (Intercept) | -41.7 | 29.8 | 0.163 | -100.2 - 16.9 |
| 2 | Age | 0.0 | 0.1 | 0.936 | -0.2 - 0.2 |
| 3 | EQ-5D-5L: English | -4.1 | 2.2 | 0.059 | -8.4 - 0.2 |
| 4 | EQ-5D-5L: Chinese | -2.6 | 2.2 | 0.237 | -6.8 - 1.7 |
| 5 | Education: Secondary level | -4.1 | 2.0 | **0.040** | -8 - -0.2 |
| 6 | Education: Tertiary level | -2.1 | 2.8 | 0.449 | -7.7 - 3.4 |
| 7 | Recurrent HF | 3.8 | 2.2 | 0.088 | -0.6 - 8.1 |
| 8 | LVEF | 0.2 | 0.1 | 0.065 | 0 - 0.4 |
| 9 | NYHA 2 | -0.6 | 9.8 | 0.952 | -19.8 - 18.7 |
| 10 | NYHA 3 | 16.7 | 18.2 | 0.361 | -19.2 - 52.5 |
| 11 | NYHA 4 | -26.3 | 12.5 | **0.037** | -50.9 - -1.6 |
| 12 | CCI ≥ 3 | 2.7 | 2.2 | 0.224 | -1.7 - 7.1 |
| 13 | Lung disease | -5.4 | 3.2 | 0.096 | -11.7 - 0.9 |
| 14 | Anaemia | -4.8 | 1.8 | **0.010** | -8.4 - -1.1 |
| 15 | Serum sodium | 0.5 | 0.2 | **0.014** | 0.1 - 0.9 |
| 16 | Serum urea | 0.8 | 0.3 | **0.006** | 0.2 - 1.3 |
| 17 | eGFR | 0.2 | 0.1 | **0.002** | 0.1 - 0.3 |
| 18 | Pre-admission ARNI | -4.5 | 2.5 | 0.070 | -9.4 - 0.4 |
| 19 | Pre-admission SGLT-2i | 1.8 | 2.2 | 0.406 | -2.5 - 6.1 |
| 20 | Pre-admission trimetazidine | -7.5 | 3.0 | **0.014** | -13.4 - -1.5 |
| 21 | Discharged ARB | -7.1 | 2.9 | **0.013** | -12.8 - -1.5 |
| 22 | Discharged statin | 3.1 | 2.1 | 0.136 | -1 - 7.2 |
| 23 | Time: Discharge | 28.8 | 1.8 | **<0.001** | 25.3 - 32.3 |
| 24 | Time: 1MPD | 30.7 | 1.8 | **<0.001** | 27.1 - 34.3 |
| 25 | Age*NYHA 2 | -0.2 | 0.2 | 0.255 | -0.5 - 0.1 |
| 26 | Age*NYHA 3 | -0.5 | 0.3 | 0.097 | -1 - 0.1 |
| 27 | Age*NYHA 4 | 0.1 | 0.2 | 0.576 | -0.3 - 0.5 |

## (B) Model 2: Sensitivity analysis using AV datasets (LASSO)

|  | Variables for VAS model | Estimate | SE | p-value | 95% CI |
| --- | --- | --- | --- | --- | --- |
| 1 | (Intercept) | -57.2 | 30.7 | 0.064 | -117.7 - 3.3 |
| 2 | Age | 0.0 | 0.1 | 0.959 | -0.2 - 0.2 |
| 3 | EQ-5D-5L: English | -4.9 | 2.1 | **0.018** | -9 - -0.9 |
| 4 | EQ-5D-5L: Chinese | -3.2 | 2.9 | 0.286 | -9 - 2.6 |
| 5 | Education: Secondary level | -4.1 | 2.3 | 0.071 | -8.6 - 0.3 |
| 6 | Education: Tertiary level | -2.0 | 2.2 | 0.375 | -6.3 - 2.4 |
| 7 | Recurrent HF | 3.2 | 2.3 | 0.165 | -1.3 - 7.6 |
| 8 | LVEF | 0.3 | 0.1 | **0.022** | 0 - 0.5 |
| 9 | NYHA 2 | -1.0 | 9.8 | 0.915 | -20.4 - 18.3 |
| 10 | NYHA 3 | 27.5 | 17.6 | 0.121 | -7.2 - 62.1 |
| 11 | NYHA 4 | -31.7 | 13.1 | **0.017** | -57.6 - -5.8 |
| 12 | CCI ≥ 3 | 1.3 | 2.3 | 0.560 | -3.1 - 5.8 |
| 13 | Lung disease | -5.1 | 3.2 | 0.113 | -11.4 - 1.2 |
| 14 | Anaemia | -4.3 | 1.9 | **0.026** | -8 - -0.5 |
| 15 | Serum sodium | 0.6 | 0.2 | **0.002** | 0.2 - 1 |
| 16 | Serum urea | 0.7 | 0.3 | **0.015** | 0.1 - 1.3 |
| 17 | eGFR | 0.2 | 0.1 | **0.014** | 0 - 0.3 |
| 18 | Pre-admission ARNI | -3.3 | 2.6 | 0.203 | -8.3 - 1.8 |
| 19 | Pre-admission SGLT-2i | 0.8 | 2.2 | 0.709 | -3.6 - 5.2 |
| 20 | Pre-admission trimetazidine | -6.3 | 3.3 | 0.058 | -12.8 - 0.2 |
| 21 | Discharged ARB | -9.4 | 3.0 | **0.002** | -15.3 - -3.6 |
| 22 | Discharged statin | 2.9 | 2.1 | 0.179 | -1.3 - 7 |
| 23 | Time: Discharge | 28.9 | 1.8 | **<0.001** | 25.3 - 32.5 |
| 24 | Time: 1MPD | 31.3 | 1.8 | **<0.001** | 27.8 - 34.9 |
| 25 | Age*NYHA 2 | -0.2 | 0.2 | 0.292 | -0.5 - 0.1 |
| 26 | Age*NYHA 3 | -0.6 | 0.3 | **0.029** | -1.1 - -0.1 |
| 27 | Age*NYHA 4 | 0.2 | 0.2 | 0.331 | -0.2 - 0.6 |

## (C) Model 3: Sensitivity analysis using MI datasets (literature and clinical relevance)

|  | Variables for VAS model | Estimate | SE | p-value | 95% CI |
| --- | --- | --- | --- | --- | --- |
| 1 | (Intercept) | 39.5 | 10.1 | **<0.001** | 19.6 - 59.4 |
| 2 | Age | 0.0 | 0.1 | 0.819 | -0.2 - 0.2 |
| 3 | Female | 1.8 | 2.3 | 0.430 | -2.7 - 6.4 |
| 4 | Chinese | -3.5 | 5.6 | 0.527 | -14.5 - 7.4 |
| 5 | Indian | -2.8 | 2.8 | 0.323 | -8.3 - 2.7 |
| 6 | EQ-5D-5L: English | -3.0 | 2.5 | 0.237 | -7.9 - 2 |
| 7 | EQ-5D-5L: Chinese | -0.3 | 5.9 | 0.955 | -11.9 - 11.2 |
| 8 | Working | 1.7 | 2.4 | 0.480 | -3.1 - 6.5 |
| 9 | NYHA2 | -1.2 | 10.2 | 0.907 | -21.3 - 18.9 |
| 10 | NYHA3 | 8.3 | 19.0 | 0.664 | -29.1 - 45.6 |
| 11 | NYHA4 | -21.6 | 13.6 | 0.113 | -48.5 - 5.2 |
| 12 | CCI | -0.2 | 0.6 | 0.783 | -1.4 - 1.1 |
| 13 | Ischaemic heart disease | -0.9 | 2.1 | 0.665 | -4.9 - 3.2 |
| 14 | Serum haemoglobin | 0.4 | 0.5 | 0.362 | -0.5 - 1.4 |
| 15 | Pre-admission platelet | -2.6 | 2.0 | 0.200 | -6.5 - 1.4 |
| 16 | Discharged ARNI | 2.2 | 1.9 | 0.249 | -1.6 - 6.1 |
| 17 | Discharge beta-blocker | 2.7 | 3.1 | 0.397 | -3.5 - 8.8 |
| 18 | Time: Discharge | 28.8 | 1.8 | **<0.001** | 25.3 - 32.3 |
| 19 | Time: 1MPD | 30.7 | 1.8 | **<0.001** | 27.1 - 34.3 |
| 20 | Age*NYHA 2 | -0.14 | 0.16 | 0.389 | -0.5 - 0.2 |
| 21 | Age*NYHA 3 | -0.34 | 0.29 | 0.247 | -0.9 - 0.2 |
| 22 | Age*NYHA 4 | 0.09 | 0.21 | 0.660 | -0.3 - 0.5 |

# Online Resource 14: Plot density for unadjusted mean utility values and VAS scores

## (A) Unadjusted mean utility values


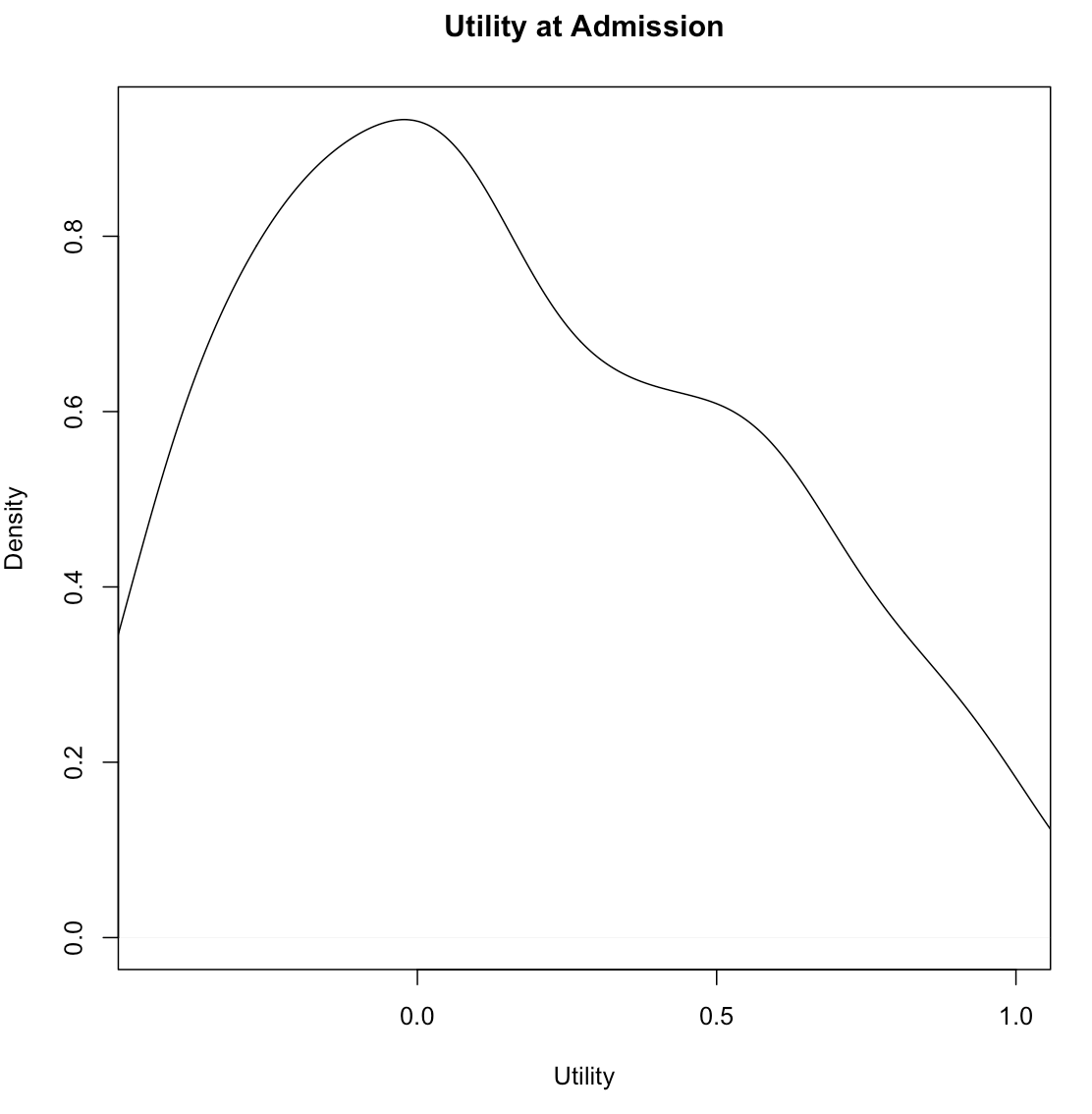

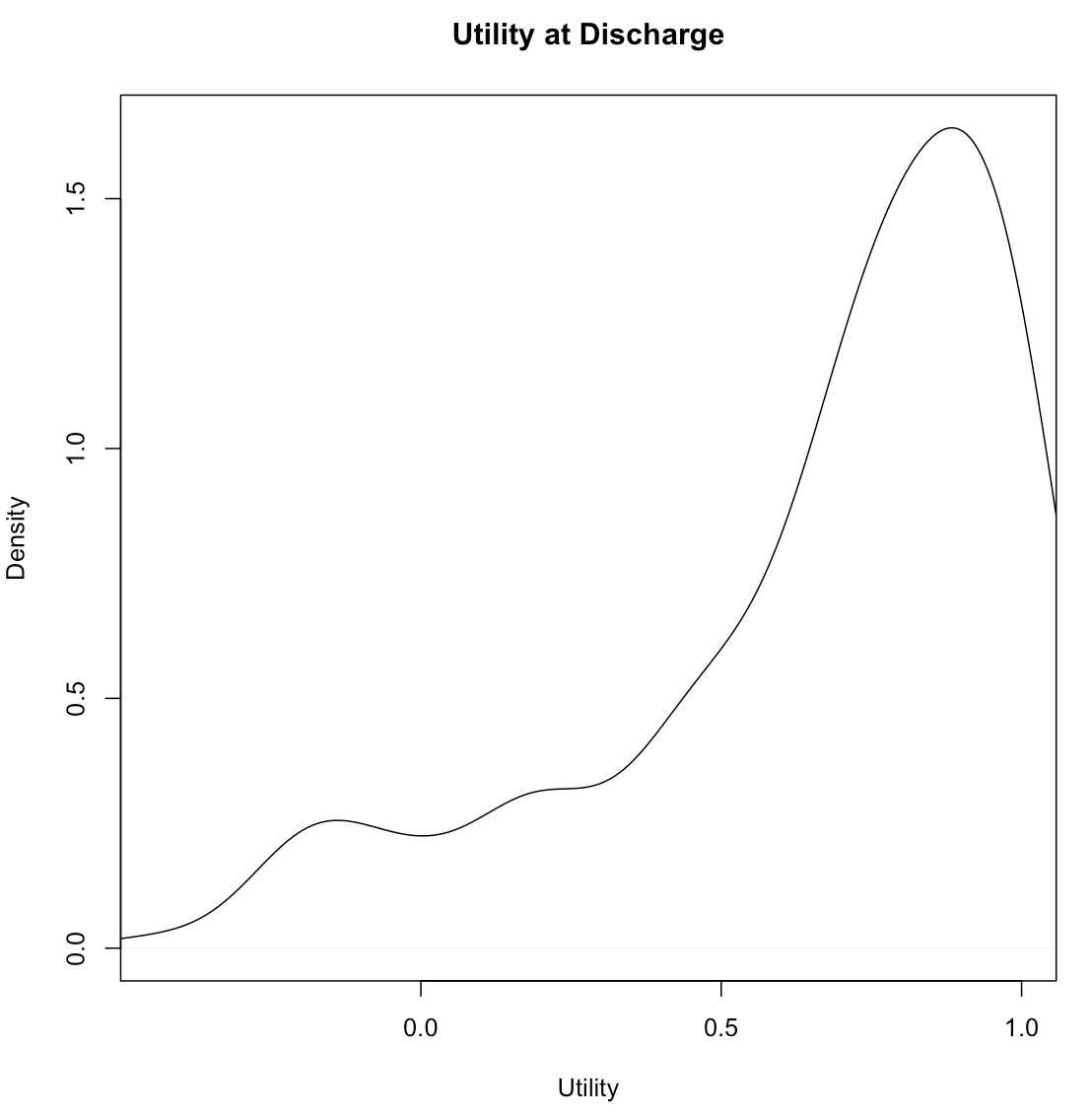

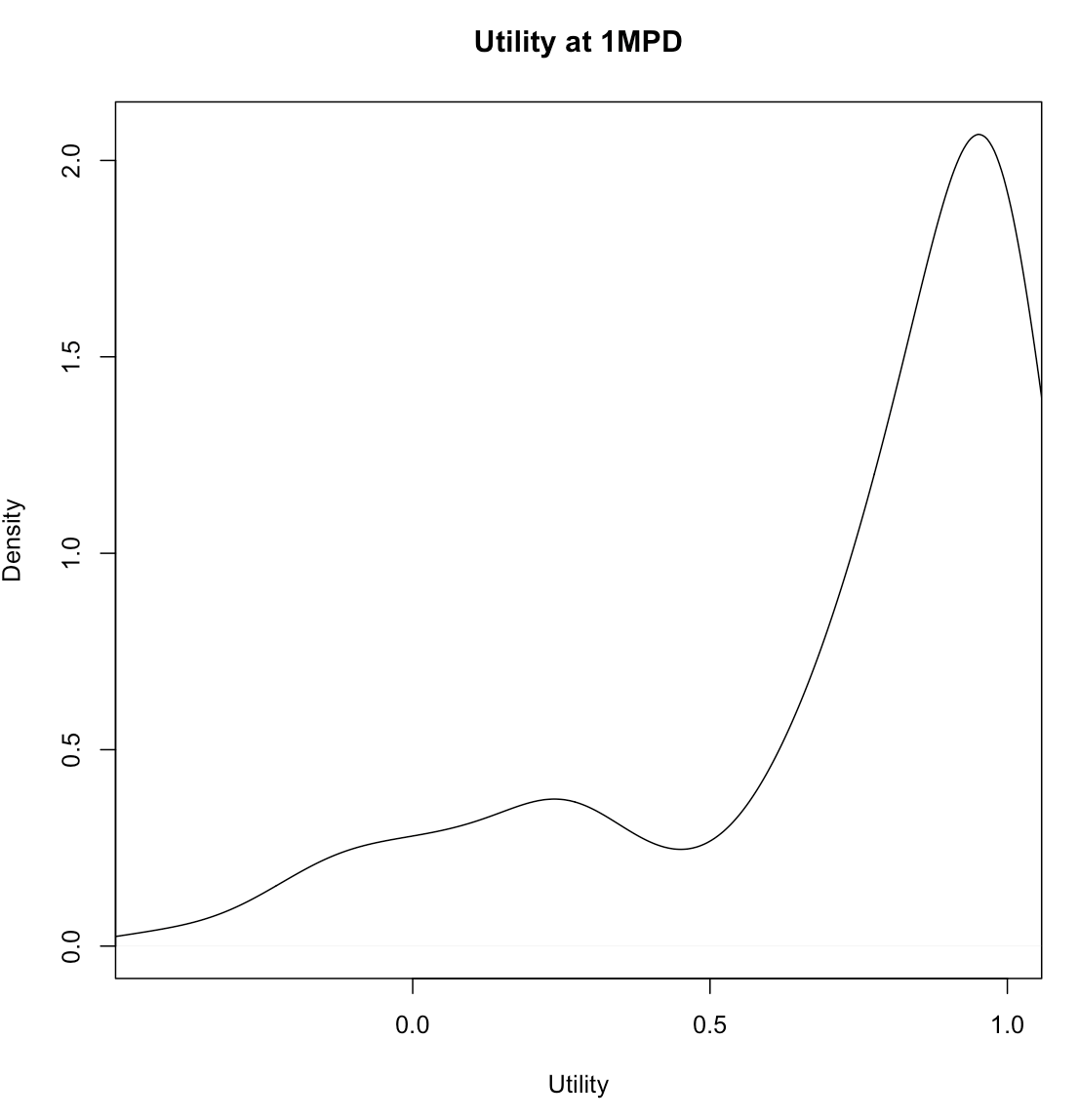


## (B) Unadjusted mean VAS scores


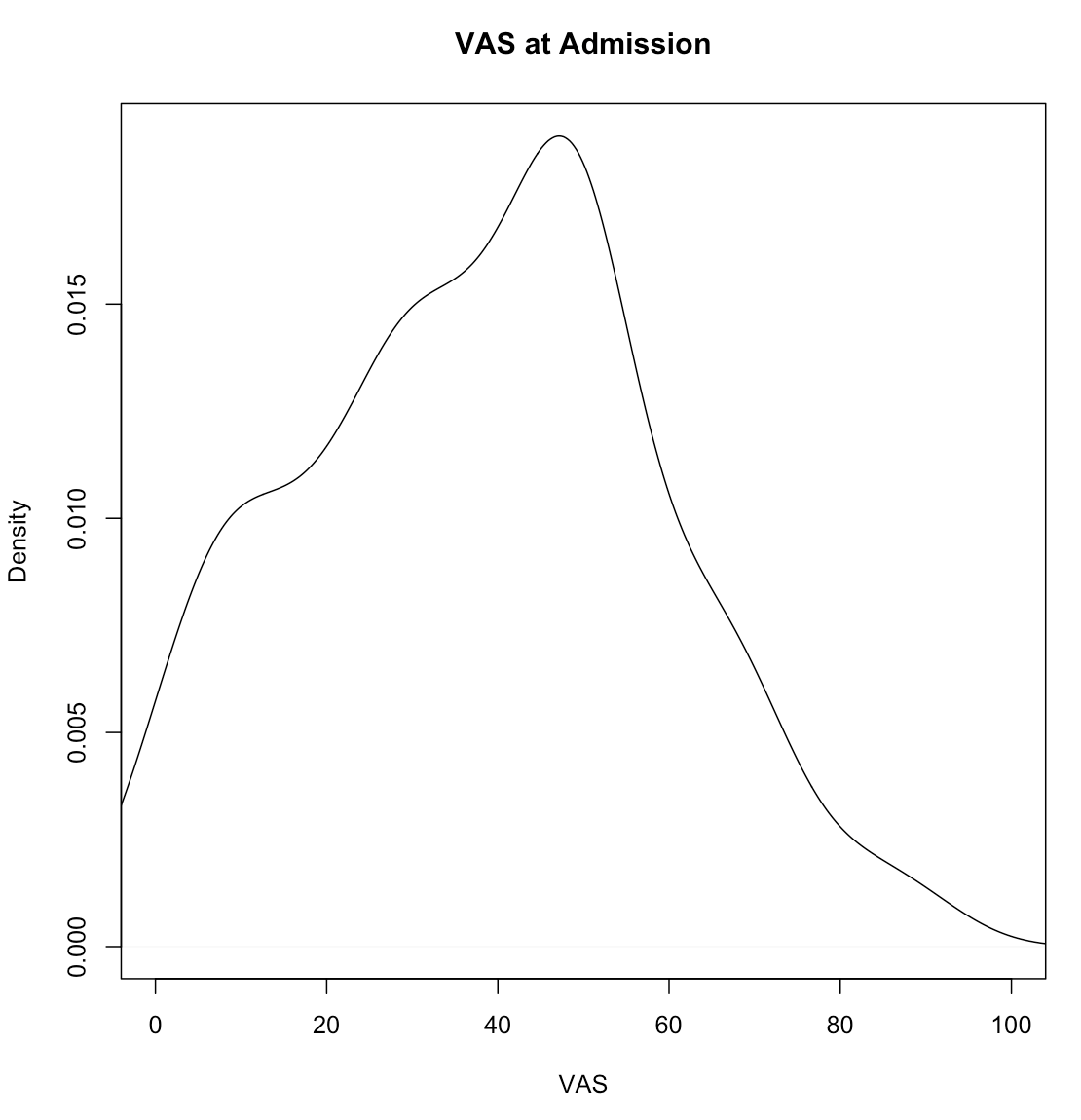

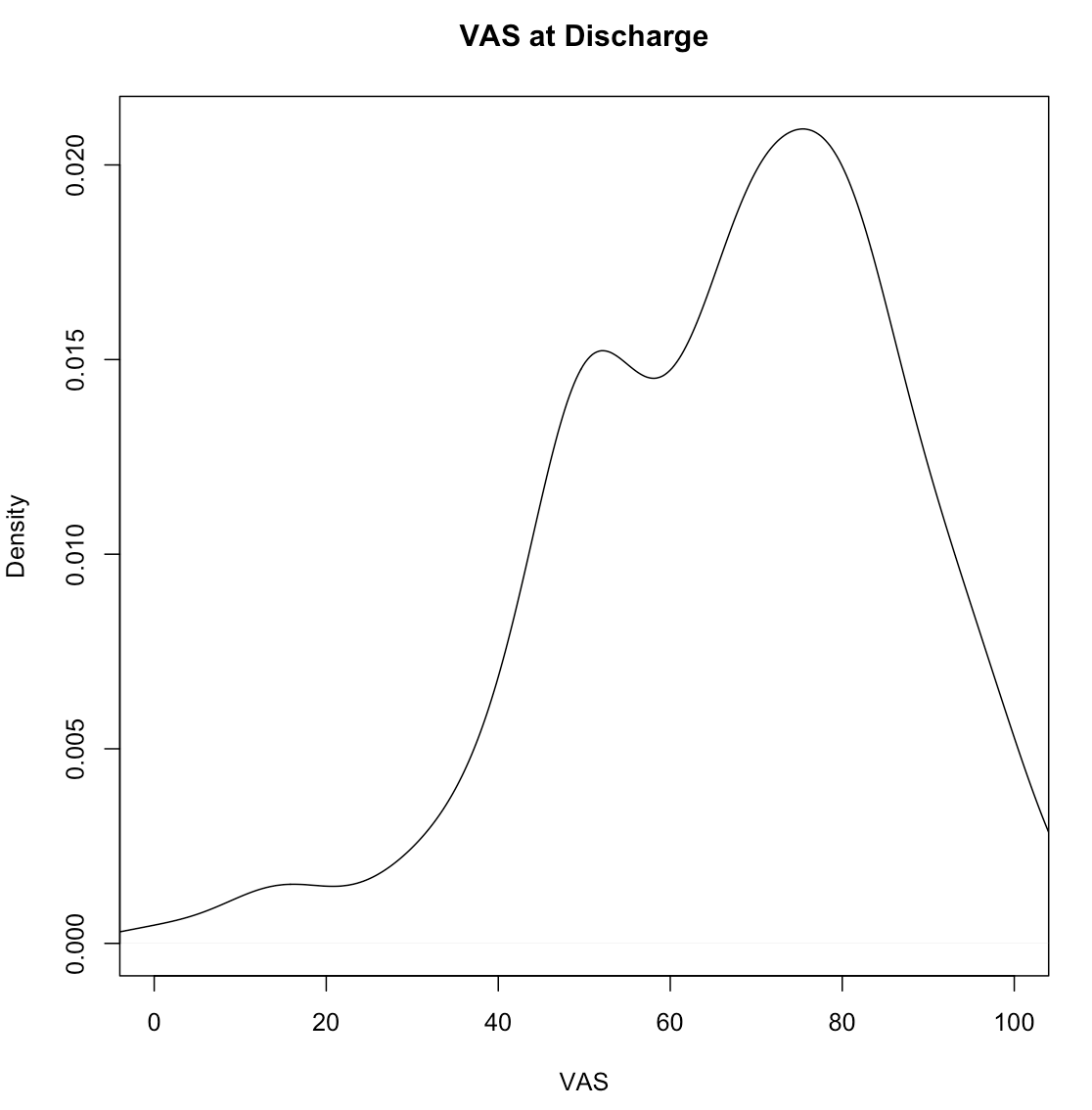

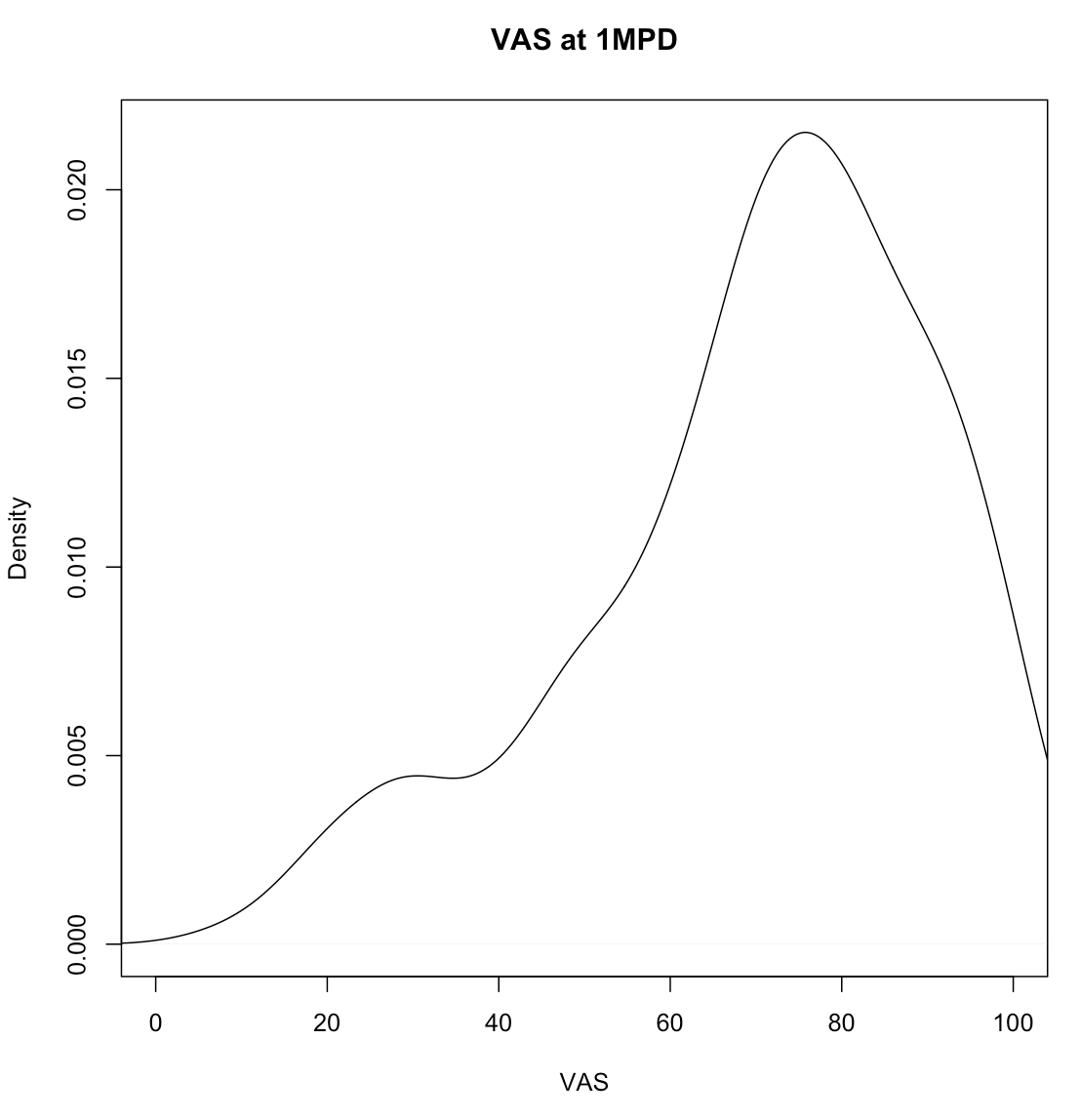


## (C) Unadjusted mean change in utility values


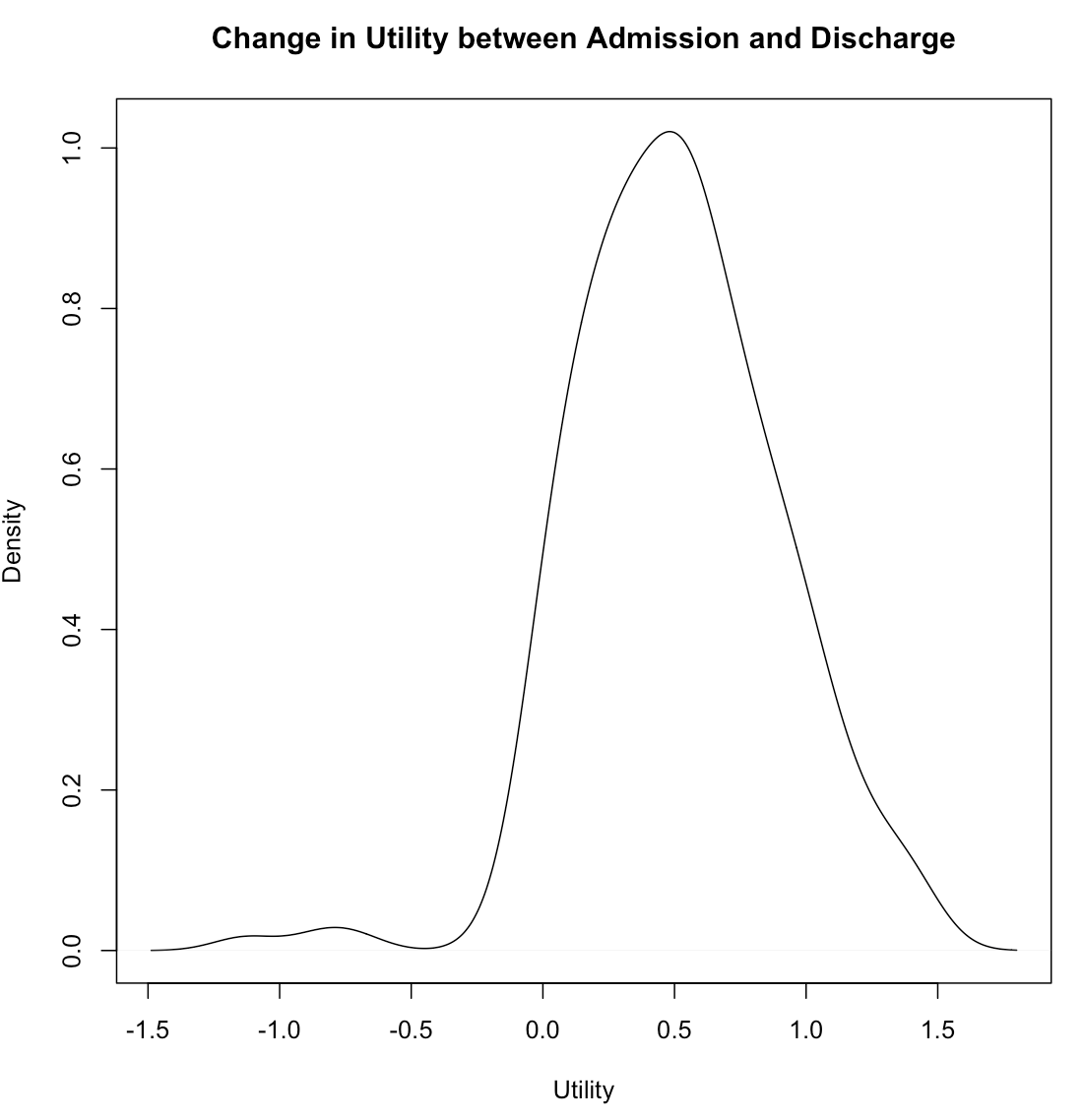

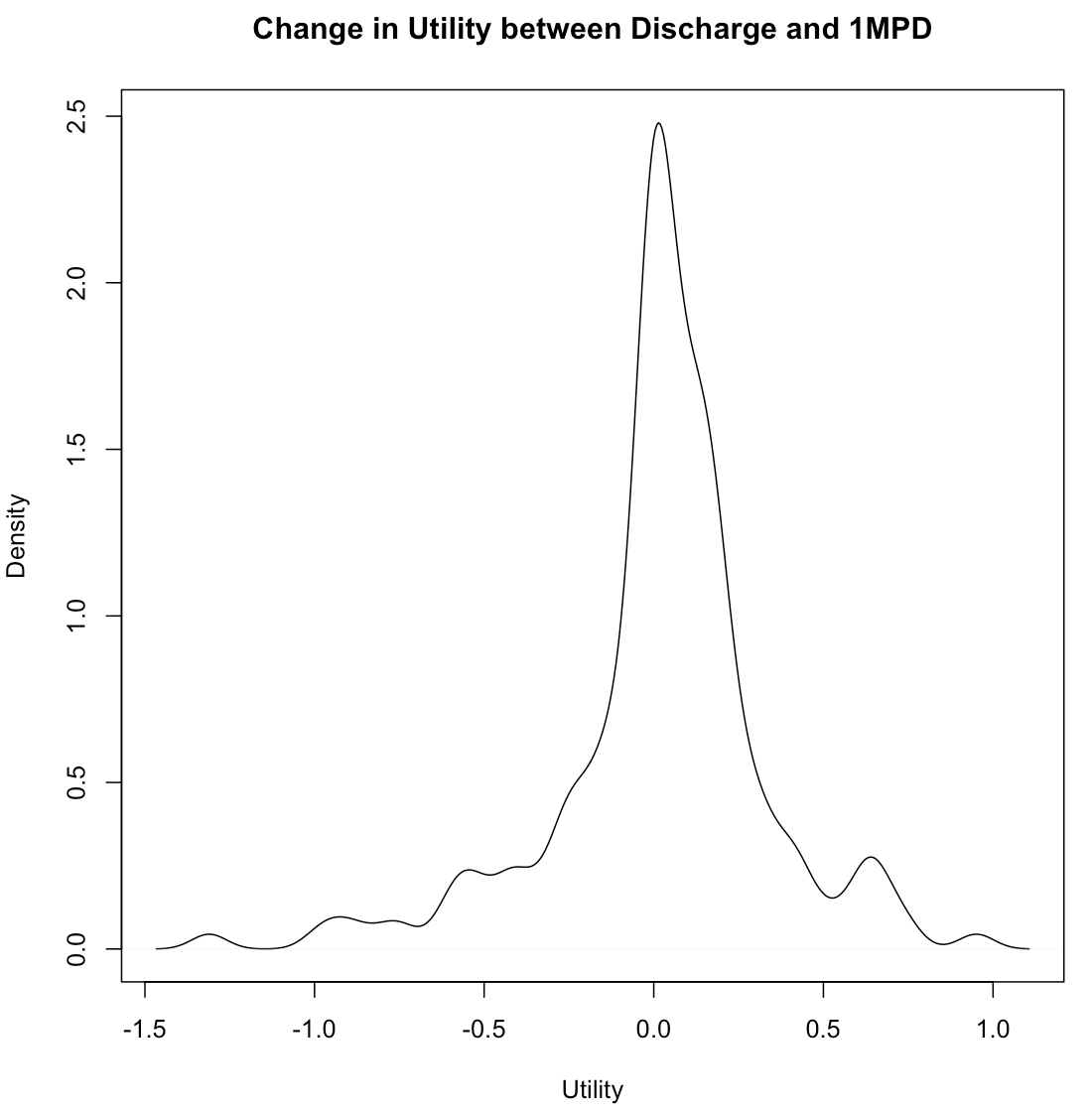

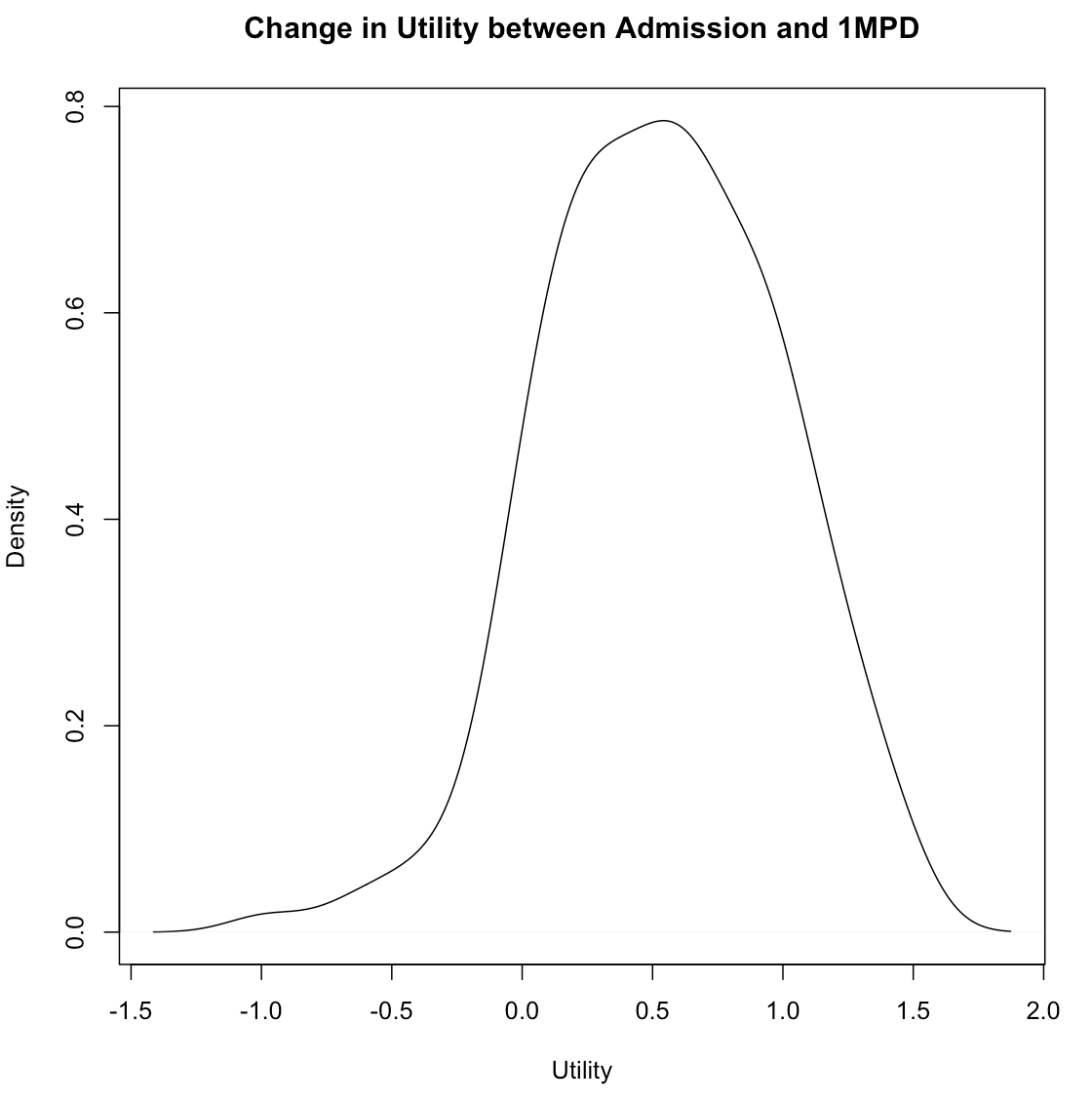


## (D) Unadjusted mean change in VAS scores


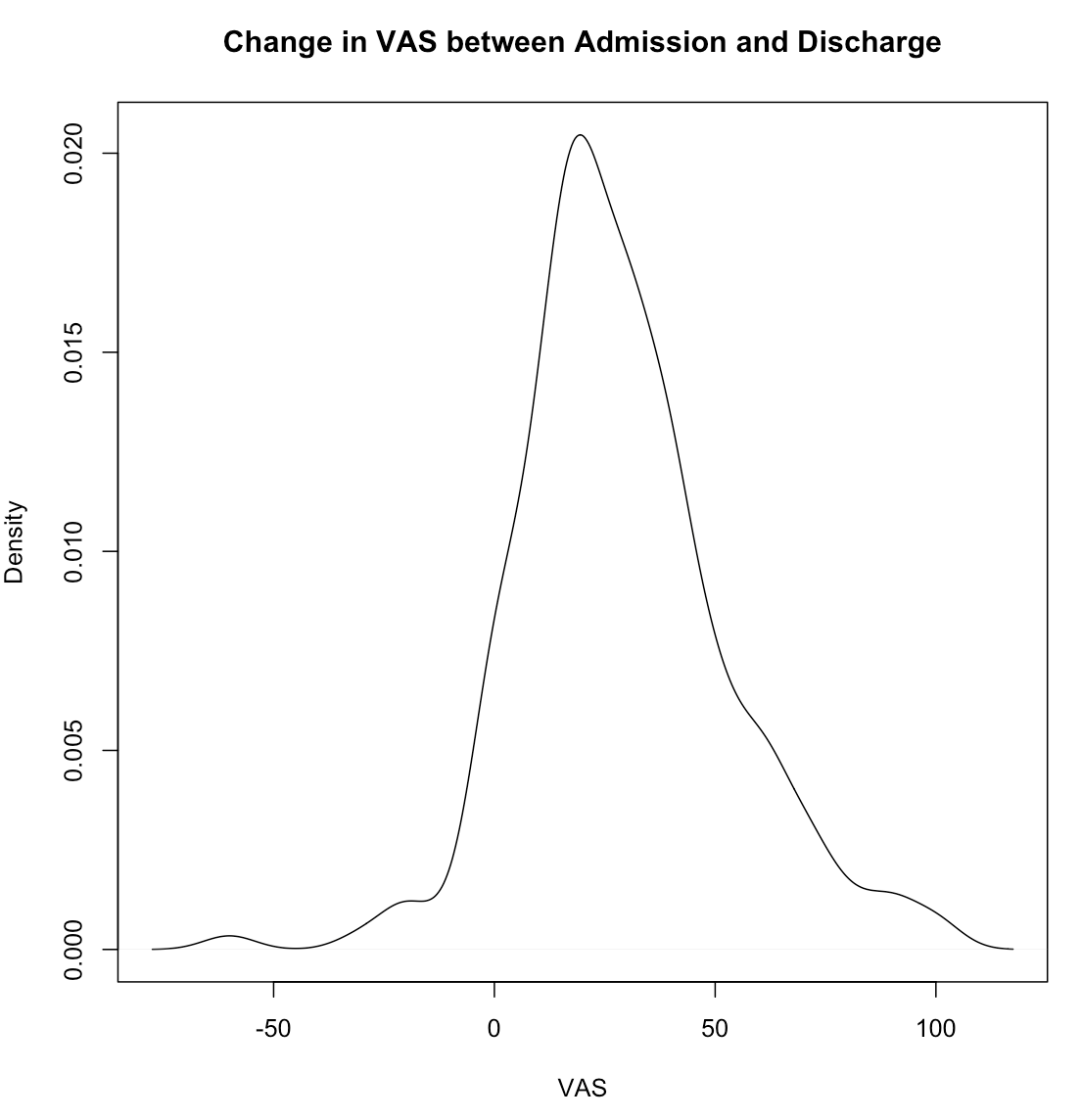

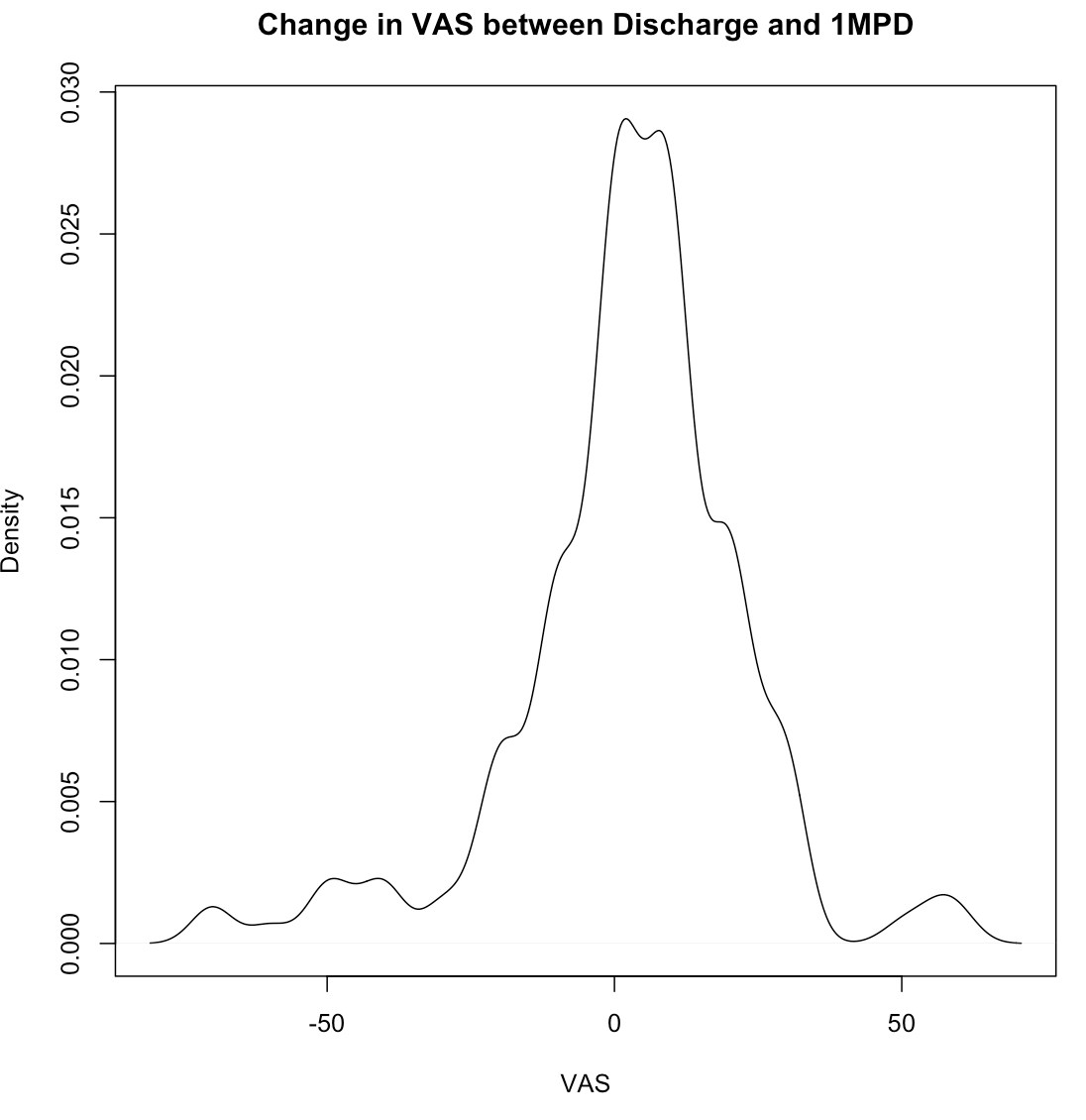

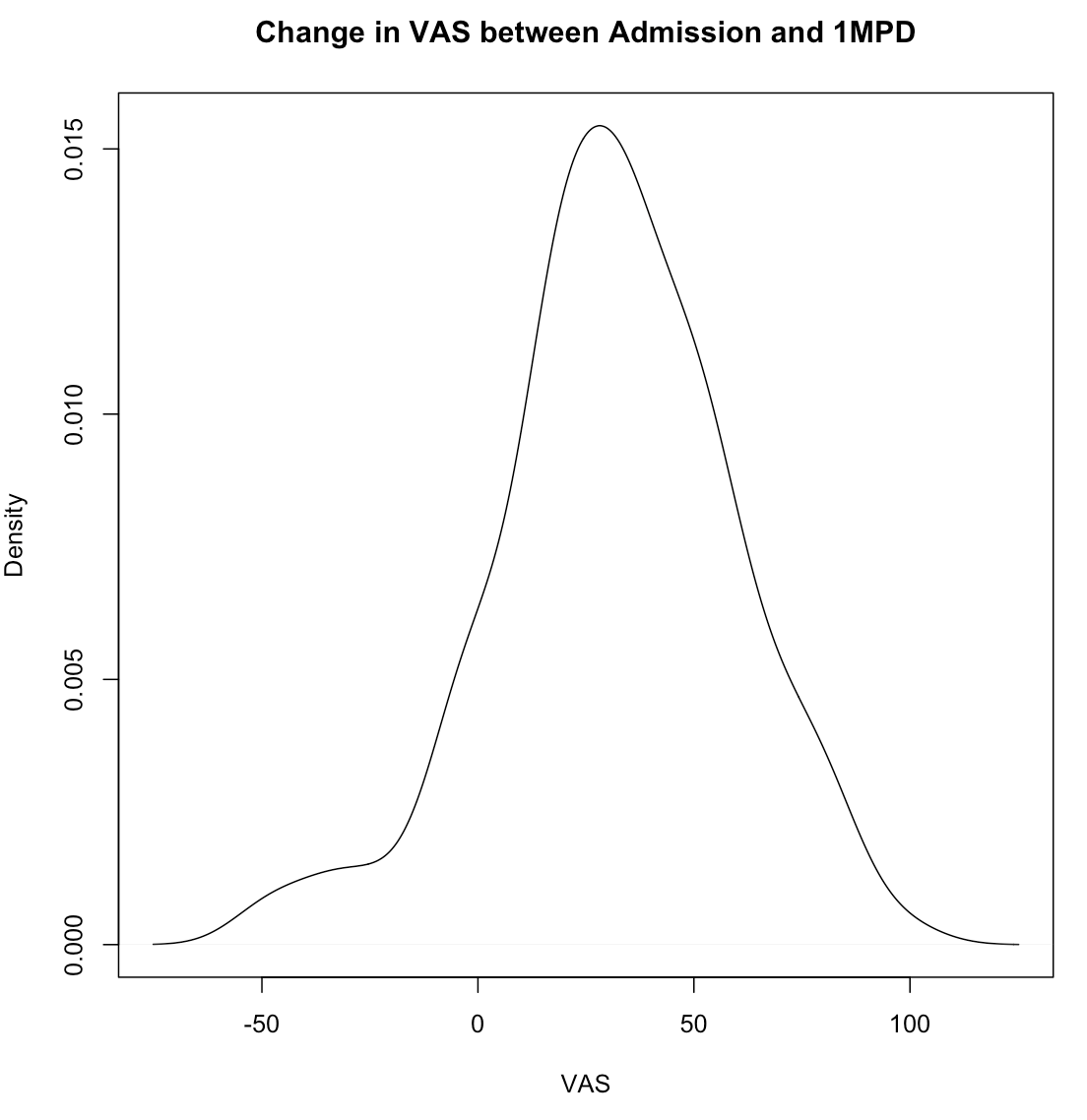


# References

1. Wei, D., Sun, Y., Chen, R., Meng, Y., & Wu, M. (2023) The Charlson comorbidity index and short-term readmission in patients with heart failure: A retrospective cohort study. *Medicine (Baltimore), 102*, e32953. <https://doi.org/10.1097/MD.0000000000032953>

2. Rösel, I., Serna-Higuita, L., Al Sayah, F., Buchholz, M., Buchholz, I., Kohlmann, T., P, M., & Feng, Y. (2022) What difference does multiple imputation make in longitudinal modeling of EQ-5D-5L data? Empirical analyses of simulated and observed missing data patterns. *Quality of Life Research, 31*(5), 1521-1532. <https://doi.org/10.1007/s11136-021-03037-3>

3. Simons, C. L., Rivero-Arias, O., Yu, L.-M., & Simon, J. (2014) Multiple imputation to deal with missing EQ-5D-3L data: Should we impute individual domains or the actual index? *Quality of Life Research, 24*(4), 805-815. <https://doi.org/doi:10.1007/s11136-014-0837-y>

4. Sepehrvand, N. S., Anamaria; Spertus, JA; Dyck, JRB; Anderson,T; Howlett,Jonathan (2020) Change of Health-Related Quality of Life Over Time and Its Association With Patient Outcomes in Patients With Heart Failure. *Journal of the American Heart Association, 9*(17), e017278. <https://doi.org/10.1161/JAHA.120.017278>

5. Mukherjee, K., Gunsoy, N. B., Kristy, R. M., Cappelleri, J. C., Roydhouse, J., Stephenson, J. J., Vanness, D. J., Ramachandran, S., NC, O., SR, P., H, K., & GL, D. T. (2023) Handling Missing Data in Health Economics and Outcomes Research (HEOR): A Systematic Review and Practical Recommendations. *Pharmacoeconomics, 41*(12). <https://doi.org/10.1007/s40273-023-01297-0>

6. White, I. R., Royston, P., & Wood, A. M. (2011) Multiple imputation using chained equations: Issues and guidance for practice. *Stat Med, 30*(4), 377-399. <https://doi.org/10.1002/sim.4067>

7. Rösel, I., Serna-Higuita, L., Al Sayah, F., Buchholz, M., Buchholz, I., Kohlmann, T., P, M., & Feng, Y. (2022) What difference does multiple imputation make in longitudinal modeling of EQ-5D-5L data? Empirical analyses of simulated and observed missing data patterns. *Quality of life research : an international journal of quality of life aspects of treatment, care and rehabilitation, 31*(5), 1521-1532. <https://doi.org/10.1007/s11136-021-03037-3>

8. Boczor, S., Eisele, M., Rakebrandt, A., Menzel, A., Blozik, E., Träder, J.-M., Störk, S., Herrmann-Lingen, C., & Scherer, M. (2021) Prognostic factors associated with quality of life in heart failure patients considering the use of the generic EQ-5D-5L™ in primary care: new follow-up results of the observational RECODE-HF study. *BMC Family Practice, 22*(1), 1-11. <https://doi.org/doi:10.1186/s12875-021-01554-1>

9. Berg, J., Lindgren, P., Mejhert, M., Edner, M., Dahlström, U., & Kahan, T. (2015) Determinants of Utility Based on the EuroQol Five-Dimensional Questionnaire in Patients with Chronic Heart Failure and Their Change Over Time: Results from the Swedish Heart Failure Registry. *Value in Health, 18*(4), 439-448. <https://doi.org/10.1016/j.jval.2015.02.003>

10. Moore, J. F., & Weatherford, L. R. *Decision Modeling with Microsoft Excel* (6th ed.). Pearson.

11. Hay, J. W., Gong, C. L., Jiao, X., Zawadzki, N. K., Zawadzki, R. S., Pickard, A. S., Xie, F., Crawford, S. A., & Gu, N. Y. (2021) A US Population Health Survey on the Impact of COVID-19 Using the EQ-5D-5L. *Journal of General Internal Medicine, 36*(5), 1292-1301. <https://doi.org/doi:10.1007/s11606-021-06674-z>

12. Kuan, W. C., Chee, K. H., Kasim, S., Lim, K. K., Dujaili, J. A., Lee, K. K.-C., & Teoh, S. L. (2024) Validity and measurement equivalence of EQ-5D-5L questionnaire among heart failure patients in Malaysia: a cohort study. *Journal of Medical Economics*, 1-16. <https://doi.org/10.1080/13696998.2024.2337563>

13. Cheng, L. J., Pan, T., Chen, L. A., Cheng, J. Y., Mulhern, B., Devlin, N., & Luo, N. (2024) The Ceiling Effects of EQ-5D-3L and 5L in General Population Health Surveys: A Systematic Review and Meta-analysis. *Value in Health*. <https://doi.org/10.1016/j.jval.2024.02.018>

14. Martín-Fernández, J., Morey-Montalvo, M., Tomás-García, N., Martín-Ramos, E., Muñoz-García, J. C., Polentinos-Castro, E., Rodríguez-Martínez, G., Arenaza, J. C., García-Pérez, L., Magdalena-Armas, L., & Bilbao, A. (2020) Mapping analysis to predict EQ-5D-5 L utility values based on the Oxford Hip Score (OHS) and Oxford Knee Score (OKS) questionnaires in the Spanish population suffering from lower limb osteoarthritis. *Health Qual Life Outcomes, 18*(1), 184. <https://doi.org/10.1186/s12955-020-01435-8>

15. Pullenayegum, E., Tarride, J., Xie, F., Goeree, R., Gerstein, H., & O'Reilly, D. (2010) Analysis of health utility data when some subjects attain the upper bound of 1: are Tobit and CLAD models appropriate? *Value in health : the journal of the International Society for Pharmacoeconomics and Outcomes Research, 13*(4), 487-494. <https://doi.org/10.1111/j.1524-4733.2010.00695.x>

16. Wolowacz, S. E., Briggs, A., Belozeroff, V., Clarke, P., Doward, L., Goeree, R., Lloyd, A., & Norman, R. (2016) Estimating Health-State Utility for Economic Models in Clinical Studies: An ISPOR Good Research Practices Task Force Report. *Value in Health, 19*(6), 704-719. <https://doi.org/https://doi.org/10.1016/j.jval.2016.06.001>

17. Alva, M., Gray, A., Mihaylova, B., & Clarke, P. (2014) The effect of diabetes complications on health-related quality of life: the importance of longitudinal data to address patient heterogeneity. *Health economics, 23*(4). <https://doi.org/10.1002/hec.2930>

18. Tan, Y. J., Ong, S. C., Yong, V. S., Khor, W. W., Pang, L. J., Choong, Y. Y., Zameram, A. M., Tan, L. Y., Voo, J. Y. H., Lam, K. K., Yen, C. H., Wahab, M. J. A., & Abdulla, Z. B. (2024) Examining health-related quality of life in ambulatory adult patients with chronic heart failure: insights from Malaysia using EQ-5D-5L. *Quality of Life Research*. <https://doi.org/10.1007/s11136-024-03674-4>

19. Lawson, C. A., Benson, L., Squire, I., Zaccardi, F., & Ali, M. (2023) Changing health related quality of life and outcomes in heart failure by age, sex and subtype *eClinical Medicine, 64*, 102217. <https://doi.org/doi:10.1016/j.eclinm.2023.102217>

20. Wan Ahmad, W. A., Abdul Ghapar, A. K., Zainal Abidin, H. A., Karthikesan, D., Ross, N. T., S.K. Abdul Kader, M. A., Loch, A., Mahendran, K., Ramli, A. W., Ong, T. K., Mohd Amin, N. H., Lee, C. Y., Che Hassan, H. H., Zainal Abidin, S. K., Liew, H. B., Ho, W. S., & Mohd Ghazi, A. (2024) Characteristics of patients admitted with heart failure: Insights from the first Malaysian Heart Failure Registry. *ESC Heart Failure, 11*(2), 727-736. <https://doi.org/https://doi.org/10.1002/ehf2.14608>
